# Trait-based analysis of the human skin microbiome

**DOI:** 10.1186/s40168-019-0698-2

**Published:** 2019-07-05

**Authors:** Sharon Bewick, Eliezer Gurarie, JL Weissman, Jess Beattie, Cyrus Davati, Rachel Flint, Peter Thielen, Florian Breitwieser, David Karig, William F. Fagan

**Affiliations:** 1 Department of Biological Sciences, Clemson University, Clemson, SC 29631 USA; 2 Department of Biology, University of Maryland, College Park, MD 20742 USA; 3 Research and Exploratory Development Department, Johns Hopkins Applied Physics Laboratory, Laurel, MD 20723 USA; 4 Center for Computational Biology, McKusick-Nathans Institute of Genetic Medicine, Johns Hopkins School of Medicine, Baltimore, MD 21205 USA; 5 Department of Bioengineering, Clemson University, Clemson, SC 29631 USA

**Keywords:** Skin microbiome, Trait-based analysis, Enzyme activity, Substrate use, Temperature, NaCl and pH range, *Bergey*’*s Manual of Systematic Bacteriology*

## Abstract

**Background:**

The past decade of microbiome research has concentrated on cataloging the diversity of taxa in different environments. The next decade is poised to focus on microbial traits and function. Most existing methods for doing this perform pathway analysis using reference databases. This has both benefits and drawbacks. Function can go undetected if reference databases are coarse-grained or incomplete. Likewise, detection of a pathway does not guarantee expression of the associated function. Finally, function cannot be connected to specific microbial constituents, making it difficult to ascertain the types of organisms exhibiting particular traits—something that is important for understanding microbial success in specific environments. A complementary approach to pathway analysis is to use the wealth of microbial trait information collected over years of lab-based, culture experiments.

**Methods:**

Here, we use journal articles and *Bergey*’*s Manual of Systematic Bacteriology* to develop a trait-based database for 971 human skin bacterial taxa. We then use this database to examine functional traits that are over/underrepresented among skin taxa. Specifically, we focus on three trait classes—binary, categorical, and quantitative—and compare trait values among skin taxa and microbial taxa more broadly. We compare binary traits using a Chi-square test, categorical traits using randomization trials, and quantitative traits using a nonparametric relative effects test based on global rankings using Tukey contrasts.

**Results:**

We find a number of traits that are over/underrepresented within the human skin microbiome. For example, spore formation, acid phosphatase, alkaline phosphatase, pigment production, catalase, and oxidase are all less common among skin taxa. As well, skin bacteria are less likely to be aerobic, favoring, instead, a facultative strategy. They are also less likely to exhibit gliding motility, less likely to be spirillum or rod-shaped, and less likely to grow in chains. Finally, skin bacteria have more difficulty at high pH, prefer warmer temperatures, and are much less resilient to hypotonic conditions.

**Conclusions:**

Our analysis shows how an approach that relies on information from culture experiments can both support findings from pathway analysis, and also generate new insights into the structuring principles of microbial communities.

**Electronic supplementary material:**

The online version of this article (10.1186/s40168-019-0698-2) contains supplementary material, which is available to authorized users.

## Background

The development of rapid, cost-effective sequencing technology has resulted in an explosion of microbiome research over the past decade. Microbial communities are now being sampled in almost every environment imaginable, ranging from the depths of the ocean [1, 2] to outer space [3, 4]. Reflecting the tremendous scope and magnitude of microbiome research are recent initiatives such as the Human Microbiome Project (HMP) [5–9] and the Earth Microbiome Project (EMP) [10–12]. The former aims to characterize all microbes on and in the human body, and the latter seeks to describe microbiomes across the entire globe. Already, discoveries from these and other, similar efforts are proving invaluable for understanding human disease [13–16], developing novel therapeutics [17, 18], and improving agricultural yields [19–21].

Existing microbiome research tends to focus on cataloging taxonomic diversity. Microbial function, by contrast, is less well studied [22, 23]. Unfortunately, without an understanding of microbial traits and, in particular, how traits differ among different environments, it is virtually impossible to answer key biological questions, like why certain microbes live where they do [24]. Trait-based analyses, which have a long history in macroscopic ecology [25–27], allow researchers to connect ecological traits to environmental associations, helping to explain the mechanisms underlying observed microbial distributions. The sheer diversity of typical microbiomes, however, makes trait-based analysis daunting.

Several strategies have been developed to circumvent challenges associated with trait-based microbial ecology. Shot-gun sequencing studies, for example, have been queried against reference databases, including COG/KOG, KEGG, eggNOG, Pfam, and TIGRFAM, to determine overrepresented genes, proteins, operons, and higher-order cellular processes [28–35] that reflect microbial function. Meanwhile, similar efforts have been extended to amplicon sequencing using PICRUSt (Phylogenetic Investigation of Communities by Reconstruction of Unobserved States) [36] and Tax4Fun [37]—bioinformatics tools that infer microbial function based on reference databases, along with various assumptions about phylogenetic conservation. Although amplicon and shot-gun sequencing approaches appear comparable [37, 38], neither performs particularly well [38]—likely because of problems with the underlying reference databases, which are coarse-grained [38], represent only a minute fraction of microbial diversity, and are heavily biased toward a few organisms and environments [39]. More recently, machine learning techniques have been applied in an attempt correct for some of these problems and improve accuracy of trait prediction [40, 41].

Despite ongoing improvements in functional reference databases, the gold standard for defining microbial traits remains culture experiments. Decades of lab-based analyses have led to an impressive understanding of the functions of diverse microbial taxa, including many of those prevalent in microbiome studies. This information, however, is largely available through journal articles and *Bergey*’*s Manual of Systematic Bacteriology* [42–45], neither of which is methodical in its presentation of data. Recently, there has been an effort to catalog trait information in more manageable and centrally organized databases, including StrainInfo [46], which collects trait data from biological resource centers and the JGI GOLD database, which allows users to enter known information on a handful of traits, including oxygen use, motility, and Gram stain. In addition, a recent text-parsing tool was developed that collects microbial descriptions from six separate sources, and then uses this information to predict microbial traits, including confidence scores [47]. The alternate, more precise but also more work-intensive approach is to link traits determined from lab- and culture-based experiments to output from microbiome sequencing studies directly, by manually curating every organism identified in a particular metagenomics sample. Although the effort involved is immense, if curation is done in a systematic fashion, then the resulting database has added, long-term value.

Here, we introduce such a trait database for human skin microbial communities, and then use it to characterize the bacterial residents of human skin in trait space. Bacterial traits are further compared to characteristics of bacteria more broadly using a similar database generated without any bias toward a particular habitat [48]. Finally, we compare traits across different skin environments to determine whether dry, moist, and sebaceous skin sites have functionally different microbial constituents. Many of the traits that we observe in skin microbiomes are consistent with expectations. For example, skin bacteria prefer warmer habitats and have higher salt requirements, in keeping with abiotic conditions on the skin surface. Several findings, however, suggest novel biological insight. Cocci, for example, are overrepresented on skin. Bacteria that form spores and possess phosphatases, by contrast, are underrepresented. Finally, relative to bacteria as a whole, skin bacteria are more likely to be anaerobic—a feature that is reflected not only in patterns of oxygen use, but also in distributions of oxidase and catalase activity, both of which are primarily beneficial in oxygen-rich environments.

## Results

### Trait composition of the human skin microbiome

**Fig. 1:**
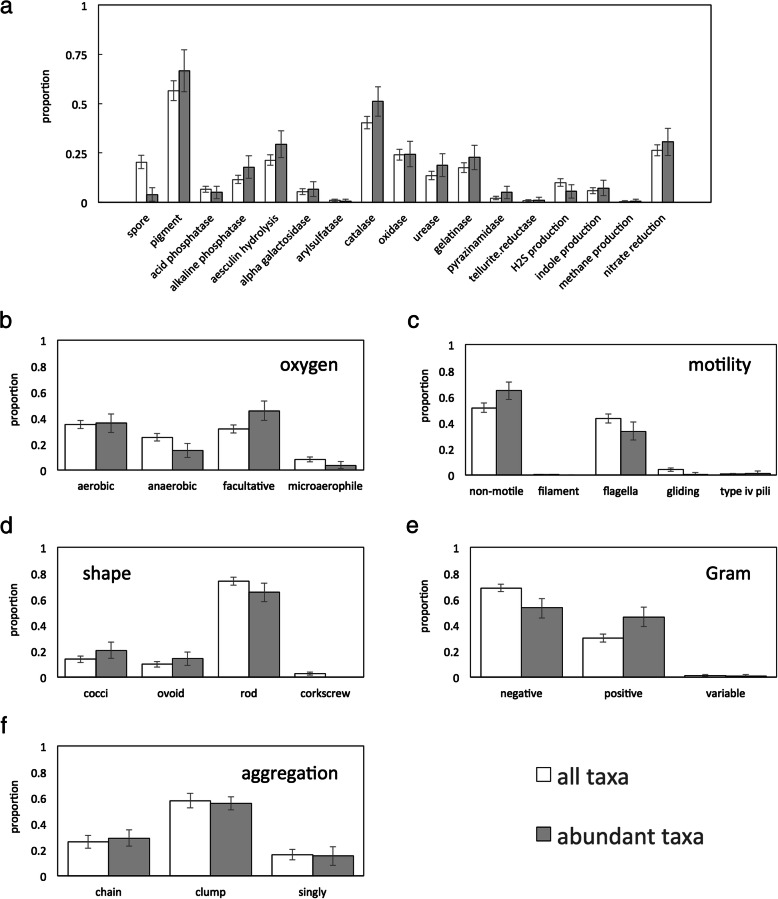
Proportion of all taxa (> 0.001% of reads in at least one sample; white) and abundant taxa (> 0.1% of reads in at least one sample; gray) in the human skin microbiome that exhibit (**a**) a range of different binary traits, (**b**) different types of oxygen use, (**c**) different types of motility, (**d**) different shapes, (**e**) different Gram stains and (**f**) different patterns of aggregation

Figure 1a presents binary traits for skin microbes. Spore formation is uncommon, particularly among abundant species, which are five times less likely to sporulate than skin microbes in general. By contrast, over half of skin taxa produce at least one pigment. Enzyme activities are varied. Whereas catalase is present in just under half of skin bacteria, oxidase, urease, alkaline phosphatase, gelatinase, and aesculin hydrolysis are less common, while acid phosphatase, α-galactosidase, arylsulfatase, pyrazinamidase, and tellurite reductase are rare. Catalase is the only enzyme more prevalent in abundant taxa. Gas production by skin bacteria is limited: almost no microbes generate methane, although a small fraction produces hydrogen sulfide and indole. Nitrate reduction is relatively common. This is in keeping with previous findings that skin commensals frequently reduce the nitrate in sweat [49, 50].

Figure 1b–f presents categorical traits for skin microbes. The majority of skin microbes are facultatively anaerobic, although there are sizeable fractions of strictly aerobic and strictly anaerobic organisms as well. Most skin microbes are also non-motile, and this is particularly true of abundant taxa. Still, an unexpectedly large proportion—approximately 40%—have flagella. No other forms of motility are strongly represented. Most skin bacteria are rod-shaped and occur in clumps. Overall, skin microbes are predominantly Gram-negative, although abundant bacteria are split equally between Gram-negative and Gram-positive taxa.

Quantitative microbial traits are given in Table 1. Optimal temperature for growth is between 33.2 and 35.0 °C, which is close to the range of mean skin surface temperature, at 32.5–35.5 °C [51]. Optimal pH is near to neutral, even for abundant bacterial species. This is surprising, because the skin is an acidic environment, with pH values ranging from 4.0 to 7.0, but generally concentrated around pH ~ 5.0 [52–54]. In fact, low pH is thought to benefit commensal skin microbes, which adhere better to the skin surface under acidic conditions [54]. Optimal salt concentrations and salt concentration ranges are, likewise, well above salt concentrations measured in sweat [55]. We hypothesize that this may be explained by sweat evaporation at the skin surface, which can concentrate the salt from sweat. Mean GC content is approximately 50%.

**Table 1:** Mean quantitative trait data for all skin bacteria (>0.001% of reads in at least one sample) and abundant skin bacteria (0.1% of reads in at least one sample)

	mean for all taxa (mean for abundant taxa)			
GC content (%)	50.6 (51.7) %			
	minimum	maximum	optimum	range
temperature (°C)	20.3 (18.8)	42.7 (42.3)	35.0 (33.2)	23.5 (25.3)
pH	5.60 (5.72)	8.30 (7.97)	7.04 (7.06)	2.74 (2.41)
NaCl concentration (%)	0.35 (1.09)	1.52 (1.68)	0.89 (1.23)	1.40 (1.14)

**Fig. 2:**
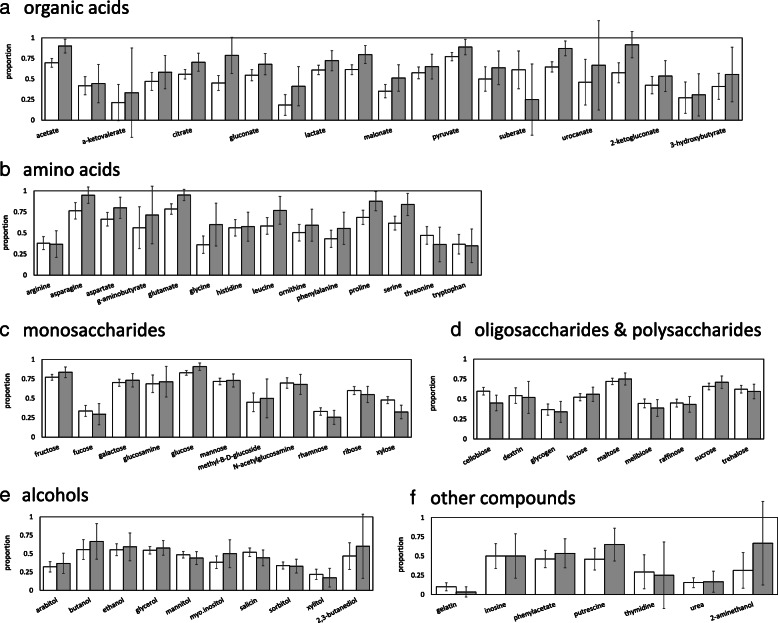
Proportion of all taxa (> 0.001% of reads in at least one sample; white) and abundant taxa (> 0.1% of reads in at least one sample; gray) in the human skin microbiome that utilize particular (**a**) organic acids, (**b**) amino acids, (**c**) monosaccharides, (**d**) oligosaccharides and polysaccharides, (**e**) alcohols and (**f**) other compounds

Figure 2 shows use of carbon substrates by skin bacteria. Here, we include all forms of use, including hydrolysis and fermentation. A wide range of carbon substrates are consumed by multiple skin taxa. This is particularly true of amino acids, with > 50% of the amino acids in our database used by > 70% of abundant skin taxa. Rates of use of monosaccharides and organic acids are lower, but still appreciable, with ~ 40% used by > 70% of abundant skin taxa. Use of alcohols and oligosaccharides/polysaccharides is less widely distributed, with 22% of oligosaccharides and no (0%) alcohols used by > 70% of abundant taxa. Of the carbon compounds considered, the substrates used most often by abundant taxa are glutamate (95%), asparagine (95%), valerate (92%), and glucose (91%).^1^ The substrates used least are gelatin (3%), urea (17%), and xylitol (17%).

Comparing abundant versus rare skin bacteria, abundant taxa are more likely to use amino and organic acids. Eight amino acids (alanine, asparagine, aspartate, glutamate, glycine, leucine, proline, and serine; see Additional file 1: Supplemental Information II Table S2.3) are used more by abundant microbes than by the skin community as whole. Similarly, nine organic acids (acetate, citrate, formate, gluconate, malate, malonate, pyruvate, succinate, and valerate; see Additional file 1: Supplemental Information II Table S2.3) are used more by abundant microbes. For both amino acids and organic acids, all significant differences indicate that abundant skin taxa use these compounds more than skin taxa as a whole. Differences in consumption of other compounds, including alcohols and saccharides, are less biased toward overuse by abundant species. Indeed, two complex sugars (xylose and cellobiose) are used less by abundant taxa. Glucose, a simple sugar, on the other hand, is used more by abundant taxa (see Additional file 1: Supplemental Information II Table S2.3).

It is well known that certain taxonomic groups, for example Actinobacteria, are overrepresented among skin microbes and, in particular, among abundant skin microbes. While these groups are likely overrepresented because they have traits that make them uniquely adapted to the skin environment, it is possible that the traits that are important for living on skin are not those that we measured. Instead, the skin relevant traits may be other traits and the differences that we observe in the traits that we did measure may merely exist as a result of phylogenetic conservation. For this reason, we performed an additional analysis regressing the probability of a taxon being abundant versus rare against each trait individually, both for a naïve logistic regression and for a regression where phylogenetic relatedness was accounted for using the phylolm package in R [56]. To test the overall significance of a fitted regression, we compared it to a null model using a likelihood ratio test. In general, we found that many of the differences between abundant and rare taxa were preserved when phylogeny was accounted for. For instance, oxygen use, spore formation, Gram stain, type of motility, H_2_S production, the presence of catalase, aesculin hydrolysis and urease, and use of succinate, acetate, gluconate (organic acids), serine, proline, and glutamate (amino acids) were significantly different among abundant and rare taxa, whether or not phylogeny was considered. A few traits were not significant once phylogeny was included, for example cell shape, the presence of alkaline phosphatase, pyrazinamidase and gelatinase, and use of xylose, glucose, cellobiose (saccharides), malonate, formate, valerate, pyruvate, citrate, aspartate (organic acids), asparagine, alanine, leucine, and glycine (amino acids). Finally, use of 2-ketogluconate (organic acid) and the ability to perform nitrate reduction were only significant when accounting for phylogeny (see Additional file 1: Supplemental Information II, Table S2.1–S2.3).

### Trait overrepresentation on human skin

**Fig. 3:**
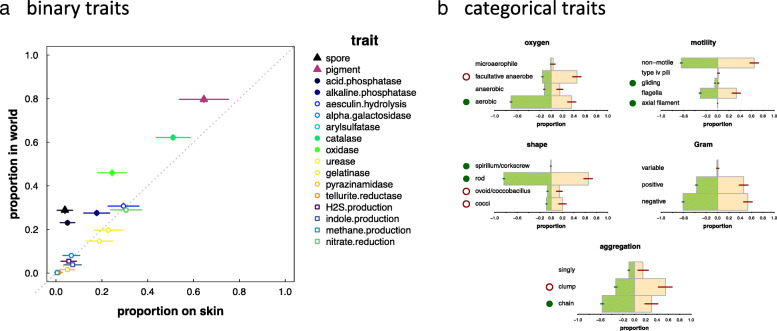
Qualitative trait comparison for abundant taxa (> 0.1% of reads in at least one sample; see also Supplementary Information I). **a** Proportion of taxa with a specific, qualitative trait in skin microbial communities (*x*-axis) versus the world as a whole (*y*-axis). Filled symbols represent traits that are significantly different in skin environments; open circles represent traits that are not significantly different; marker size reflects significance. **b** Plots of trait proportions among skin bacteria (pink) and world bacteria (green). Open red circles denote traits that are overrepresented on skin; filled green circles denote traits that are overrepresented in the world (underrepresented on skin)

Without comparison to prevalence in the world as a whole, it is impossible to know which traits are generally common versus preferentially selected for in skin environments. Figure 3a presents a comparison of binary traits among abundant skin bacteria versus bacteria more broadly (see “Materials and methods” section; see also Additional file 1: Supplemental Information III Fig. S3.1). Although there is a correlation between prevalence of a trait on skin and in the world as a whole, several traits are underrepresented among abundant skin taxa. Spore formation, for example is 7.5 times less likely among skin taxa as compared to general bacteria. Meanwhile, there is a 4.5-fold reduction in the likelihood of a skin taxon possessing acid phosphatase and a 1.5-fold reduction in the likelihood of a skin taxon possessing alkaline phosphatase as compared to bacteria more broadly. General bacteria are also 23% more likely to produce a pigment, 21% more likely to possess catalase, and 87% more likely to possess oxidase. For categorical traits, we again see significant differences between skin taxa and taxa from the world more broadly. Abundant skin bacteria (see Fig. 3b) are approximately half as likely to be aerobic, favoring, instead, a more flexible, facultative strategy. Likewise, abundant skin bacteria are 8-fold less likely to exhibit gliding motility, and none possess axial filaments, whereas these occur in ~ 0.1% of bacteria overall. Abundant skin taxa are also less likely to be spirillum or rod-shaped, whereas the fraction of cocci and coccibacilli on skin is inflated more than 2-fold. Finally, abundant skin bacteria are half as likely to grow in chains, preferring to aggregate as clumps instead.

**Fig. 4:**
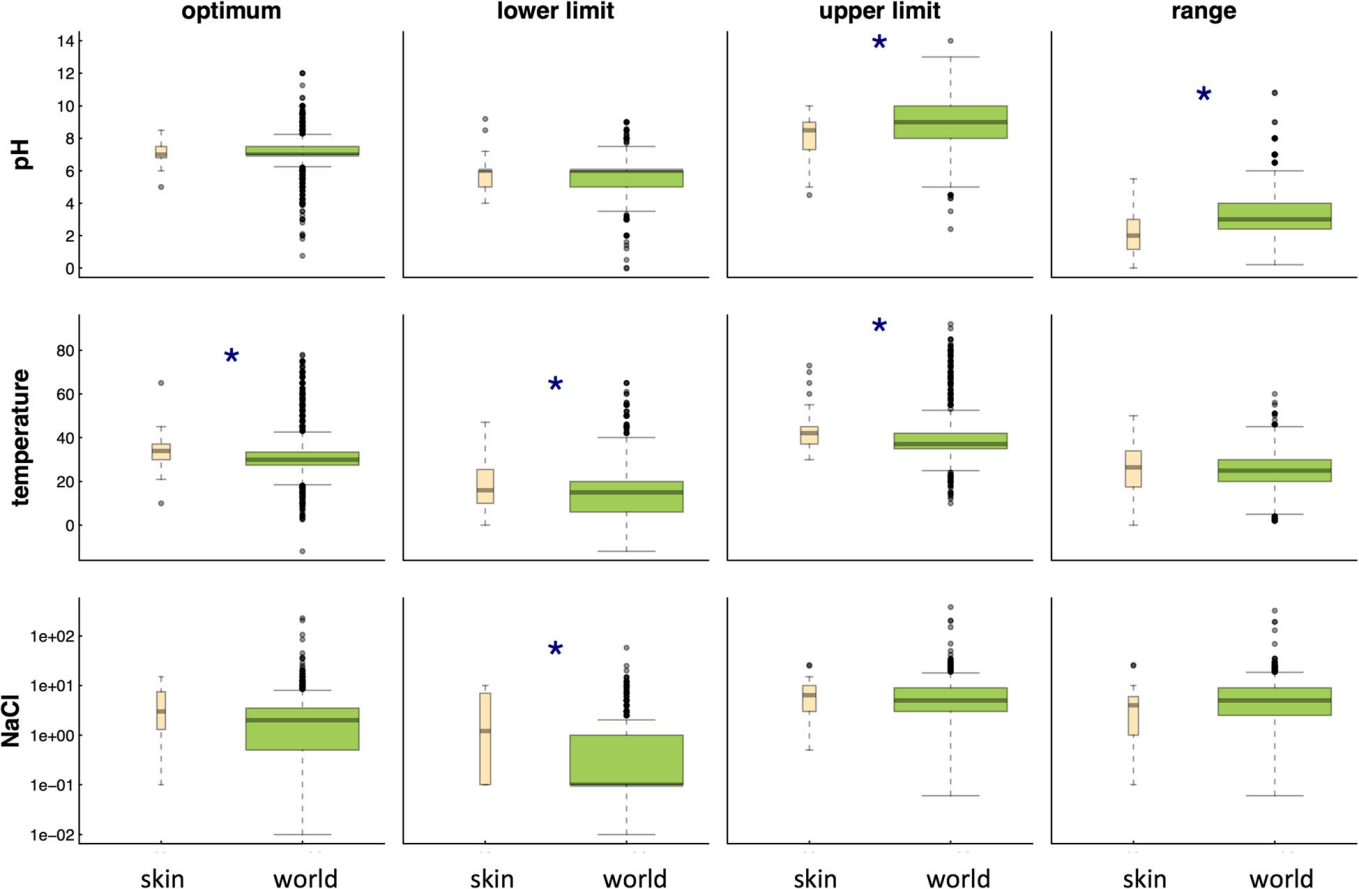
Boxplots comparing quantitative traits among skin bacteria (pink) and bacteria from the world in general (green) for abundant skin microbes (> 0.1% of reads in at least one sample; see also Supplemental Information I). Blue stars are used to denote significant differences between a trait value in the world versus on skin. Box width indicates the relative number of microbes used for the comparison

Figure 4 compares quantitative traits among world and skin bacteria (see also Additional file 1: Supplemental Information III, Figure S3.2). Abundant skin bacteria have more difficulty at high pH, tolerating, on average, a pH maximum of 7.97 versus 9.03 for the world in general. Abundant skin taxa also have a smaller range of pH values (2.41 versus 3.38) over which growth occurs. We speculate that this is because skin is a largely acidic environment with a relatively stable pH. Interestingly, however, optimal pH values for skin microbes do not reflect pH ranges measured on skin. Abundant skin bacteria also prefer warmer temperatures, can tolerate warmer temperatures, and have more difficulty at cold temperatures (with all three skin metrics being ~ + 2 °C) as compared to bacteria more broadly. Again, we hypothesize that this is because the skin is, at least relatively speaking, a warmer environment [48]. With respect to salt requirements, abundant skin bacteria are much less resilient to hypotonic conditions, requiring on average 1.1% NaCl, whereas average requirements in the world as a whole are closer to 0.02%. We speculate that this is because the skin is subject to constant excretion of salts through sweating. Finally, skin bacteria have a lower GC content (see also Additional file 1: Supplemental Information I, Figure S2), consistent with previous findings that host-associated organisms are AT-rich [57, 58].

We do not consider differences in carbon substrate usage between skin and the world because this information was collected differently in the skin database relative to the world database, making comparison impossible (see “Materials and methods” section).

### Phylum level differences

As suggested above, one explanation for observed trends in functional traits on human skin is that these result from certain phyla (*Actinobacteria*, *Bacteroidetes*, *Firmicutes*, and *Proteobacteria*) being the predominant constituents of the skin microbiome. To address this possibility, we used two separate approaches. First, we determined whether differences in functional traits between skin microbes and microbes more broadly persist when considering each phylum separately (see Tables 2, 3, and 4 and Additional file 1: Supplemental Information IV). For many traits—specifically, spore formation, pigment production, acid phosphatase, catalase (except for *Actinobacteria*), oxidase (see Table 2, Additional file 1: Table S4.1–S4.3), oxygen requirements, cell aggregation (see Table 3, Additional file 1: Table S4.4–S4.6), GC content, pH, and temperature requirements (see Table 4, Additional file 1: Table S4.7–S4.9)—biases that were apparent at the kingdom level are also apparent across multiple phyla. For other traits—for example, alkaline phosphatase, aeculin hydrolysis, and α-galactosidase (see Table 2, Additional file 1: Table S4.1–S4.3)—biases from the global composition appear driven by a single phylum, usually *Proteobacteria*, which is the most diverse phylum (see Additional file 1: Table S1.2) and thus most likely to impact overall results. Finally, for a few traits—most notably H_2_S and indole production (see Table 2, Additional file 1: Table S4.1–S4.3), motility, Gram stain, and cell shape (see Table 3, Additional file 1: Table S4.4–S4.6)—trends vary among phyla. Second, similar to our comparison of abundant versus rare taxa, we regressed the probability of a taxon being on the skin versus in the world more broadly against each trait individually using both a naïve logistic regression and a regression where phylogenetic relatedness was accounted for [56]. We then tested the overall significance of a fitted regression based on a null model using a likelihood ratio test. This analysis showed that all traits significantly over/underrepresented on skin relative to the world remained significant when accounting for phylogeny, while three traits (urease, pyrazinamidase, and nitrate reduction) were only significant under phylogenetic correction (see Additional file 1: Supplemental Information IV, Figure S4.10 and S4.11).

**Table 2:**
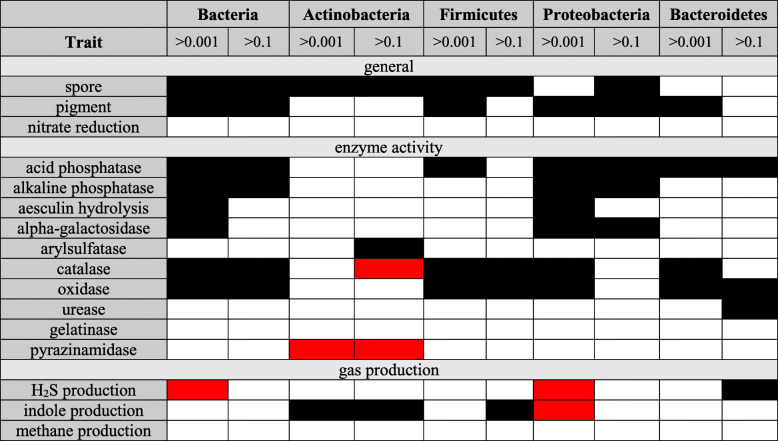
Summary of binary trait results across dominant phyla from the human skin microbiome. Black is used for traits that are over-represented in the world; red is used for traits that are over-represented in the human skin microbiome. (See Table S3.1 for more detail)

**Table 3:**
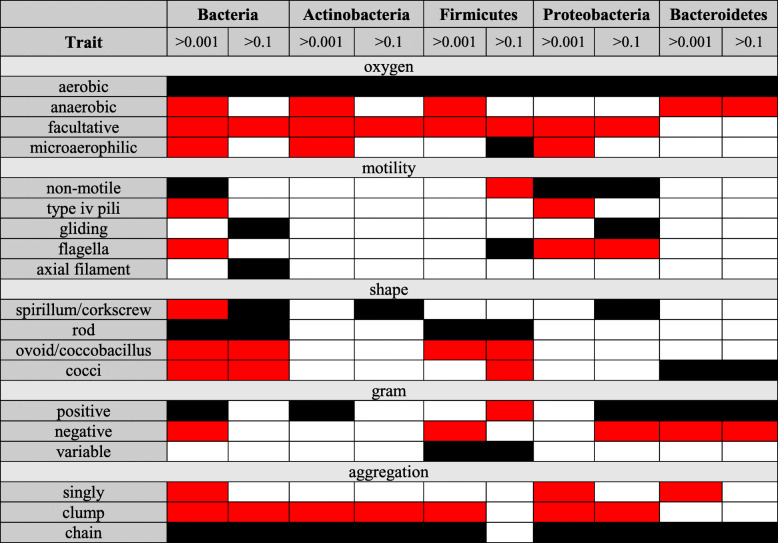
Summary of categorical trait results across dominant phyla from the human skin microbiome. Black is used for traits that are over-represented in the world; red is used for traits that are over-represented in the human skin microbiome. (See Table S3.2 for more detail)

**Table 4:**
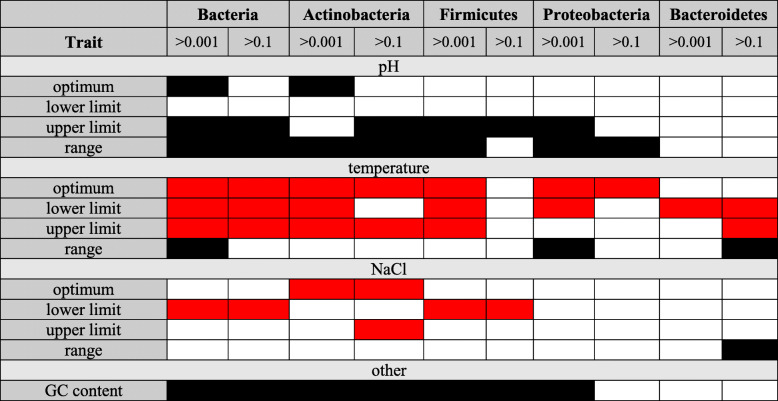
Summary of quantitative trait results across dominant phyla from the human skin microbiome. Black is used for traits that take on higher values in the world; red is used for traits that take on higher values in the human skin microbiome. (See Table S3.3 for more detail)

### Trait differences among skin sites

Human skin microbiomes generally structure according to skin environment, with three environments—dry, moist, and sebaceous—represented (see Additional file 1: Supplemental Information I, Table S1.1). Because taxonomic composition differs among these three environments, functional diversity may vary as well. To test this hypothesis, we performed pairwise comparisons (dry vs. moist, dry vs. sebaceous, and moist vs. sebaceous) for all traits and substrate utilizations in our database (see Supplemental Information V). Surprisingly, not one difference emerged among skin environments for enzyme activities, gas production, spore formation, pigment production, nitrate reduction, Gram stain, cell aggregation, or pH, temperature, and NaCl requirements (see Additional file 1: Figure S5.1i, iii, S5.2i, iii, S5.3i, iii). Abundant bacteria at sebaceous sites are less likely to be rods as compared to abundant taxa at moist sites (49% versus 68%, see Additional file 1: Figure S5.3iv). As well, anaerobes are slightly underrepresented at dry sites as compared to sebaceous sites (see Additional file 1: Figure S5.2ii), and GC content is slightly lower at dry sites as compared to moist sites (see Additional file 1: Figure S5.5), although these latter two trends only emerge when considering the full skin microbiome, not just abundant taxa. Unfortunately, when accounting for phylogeny, the model for cell shape was degenerate for abundant taxa. However, variation in oxygen use between dry and sebaceous sites was observed even with phylogenetic correction. We did not attempt to control for phylogeny for GC content, since this was a quantitative trait.

Substrate usage (see Additional file 1: Supplementary Information V, Figure S5.6–S5.11) is similarly constant among skin environments, and what few differences do exist only occur between moist and sebaceous sites. Specifically, bacterial use of three organic acids—quinate, malonate, and caprate—as well as glucosamine (a monosaccharide) is overrepresented at sebaceous sites. By contrast, bacterial use of three saccharides—rhamnose, xylose, and cellobiose—as well as glycine (an amino acid) and urea are overrepresented at moist sites.

Our finding of high similarity among skin sites is in keeping with previous studies [6], but contrasts with a KEGG analysis performed in Oh et al. [59]. The discrepancy between our trait database analysis and the KEGG analysis may be because we considered a different set of functions. Alternatively, it may be because of differences in our definition of function prevalence. In particular, Oh et al. [59] quantified commonness of pathways across samples, whereas we quantify commonness of functions across taxa. Defining prevalence across species is not possible using pathway analysis, highlighting a distinction and benefit of our trait-based approach.

## Discussion

We have undertaken a comprehensive trait-based analysis of the microbial constituents of human skin. In doing so, we have built an extensive trait-based database that will benefit future endeavors to characterize the functional properties of the skin microbiome. Below, we discuss some of our findings in terms of biological insight and interpretations.

### Catalase, oxidase, and oxygen tolerance

Catalase is the most broadly distributed enzyme across the entire skin microbiome, and the only enzyme present in a significantly higher fraction of abundant skin taxa as compared to skin taxa as a whole. This suggests that catalase may be particularly beneficial for survival on skin, which should not be surprising. The majority of human skin is exposed to oxygen, while the role of catalase is to protect cells against hydrogen peroxide (H_2_O_2_)—an oxidant primarily generated as a result of reaction between oxygen and growth substrates [60]. Interestingly, however, catalase is still less common in skin bacteria as compared to bacteria as a whole. We speculate that this is because of the existence of one or more diverse, low-oxygen niches on human skin. Further evidence for such niches comes from the markedly lower prevalence of oxidase and the increased fraction of facultative and strict anaerobes and microaerophiles found on skin (see Additional file 1: Figure S3.1). One potential low oxygen niche is sebaceous follicles. These house the classic skin anaerobe, *Propionibacterium acnes* [61], and have been previously shown to be dominated by anaerobic taxa [62]. Sequencing studies, however, have pointed to low microbial diversity within follicles [63], which is not consistent with our finding that ~ 1/3 of culturable bacterial diversity on skin is either anaerobic or microaerophilic. Thus, we hypothesize that there are additional, low-oxygen environments hosting anaerobic taxa. One potential candidate is mixed-species biofilms [64]. Another is lower dermal layers, which may have been collected through scraping of the skin [59].

Several previous studies have considered the anaerobic portion of the skin microbiome, which is of interest because of its role in wound infections [65, 66]. These studies have found that counts of aerobes outnumber counts of anaerobes [67]. Although this may seem to contradict our conclusions, our analysis is based on diversity, rather than absolute counts. Based on our work, we theorize that, though anaerobes and microaerophiles may be less abundant, they must still be quite diverse. Consistent with previous findings, we observe evidence of increased anaerobicity among microbes at sebaceous sites (see Additional file 1: Figure S5.2) [67]. Similarly, our conclusion that anaerobes are less common at dry sites (see Additional file 1: Figure S5.2) accords with the KEGG analysis performed in [59], which found that dry sites harbored an abundance of citrate cycle modules.

### Acid and alkaline phosphatases

Phosphatases enable bacteria to utilize certain components of soluble organic phosphorus [68], and thus are prevalent in environments where inorganic phosphorus is limiting. Almost 50% of microorganisms in soil and plant roots possess phosphatases [69–71]. By contrast, we find acid phosphatase in 7–8% of skin bacteria, and alkaline phosphatase in 12–13%; thus, we conjecture that phosphorus limitation is not significant in skin environments. This is surprising, because an experiment designed to measure loss of inorganic elements through healthy skin did not detect any phosphorus [72], nor is phosphorus abundant in human sweat [73, 74]. One explanation could be that skin bacteria rely on host-produced phosphatases [75, 76] to meet their needs. This would circumvent the metabolic cost of producing phosphatases, highlighting potentially unique aspects of microbial strategies in human-associated environments.

### Spore formation

In a recent review article, Lennon and Jones [77] outlined factors promoting bacterial dormancy, with spore formation being an extreme case. Unlike the human gut, where few microbial genomes (~ 15%) show evidence of sporulation [77], human skin satisfies many of the conditions for dormancy. Skin, for example, is a highly inhospitable, exposed environment, lacking in resource availability [78]. By contrast, the gut is well-fed and generally protected. Furthermore, residence times on skin are long as compared to in the gut. Despite these differences, we find that the prevalence of sporulation is similar on skin and in the gut, both of which are significantly lower than rates among bacteria more broadly (see Fig. 3). Only ~ 20% of skin taxa produce spores, and this number is drastically lower (3%) when considering abundant taxa. Clearly, then, human microbiomes favor species without sporulation. We surmise that this is a result of the constant environment provided by host homeostasis.

### Cell shape and aggregation

Relative to the broader world, skin microbiomes are enriched for cocci and coccobacilli (see Fig. 3). There are several hypotheses for why this might occur. First, rods allow for increased surface-to-volume ratios, improving nutrient uptake by passive diffusion [79] or when nutrients are directly acquired from a surface [80]. The fact that relatively fewer skin bacteria are elongated may thus indicate that nutrients on skin are readily available or, at the very least, are not acquired by passive diffusion (but see [81]). Second, although rods and filamentous cells are predicted to perform better under shear stress [82], cocci may be better able to fit into small pockets and pores of the stratum corneum. This is an alternate strategy for protection [82] that may be particularly advantageous on skin. Third, rod-shaped cells are more hydrodynamic, and thus can propel through liquid more efficiently [83]. This, however, may be of minimal importance in skin environments (although it is worth noting that rods appear to be enriched in moist regions). By contrast, cocci move much faster under conditions of Brownian motion [84]. Because skin bacteria frequently spread from one person to another through airborne release [85], a coccoid shape could facilitate interpersonal dispersal. Interestingly, coccoid cells can acquire some of the advantages of a rod shape (e.g., increased surface attachment) by growing in chains [82]. Despite this, chains, like rods, are underrepresented on human skin, further supporting our conclusion that skin selects for a spherical, rather than elongated shape.

### Substrate utilization

Although many different substrates are consumed by skin bacteria, several stand out as being particularly important for success. Bacterial use of organic and amino acids, for example, shows enrichment in abundant skin bacteria. Interestingly, all eight of the amino acids that we find used significantly more by successful skin species have been positively identified in fingerprint samples [86]. This is consistent with our conclusion that these are important skin nutrients. Similar to amino acids, many of the organic acids that are used by a greater fraction of abundant skin taxa also appear commonly on human skin. This includes lactate, pyruvate [73], formate [87], caprate, and valerate [88]. In other cases, nutrients whose use is overrepresented among abundant taxa may not be produced by human skin, but rather, by dominant skin constituents. Succinate, for example, is a skin fermentation product of *Staphylococcus epidermidis*, meaning that it is likely widely available on the skin surface [89]. Further analysis of the chemical composition of skin secretions, not only by the human host but also by the entire skin microbiome, will help elucidate our findings regarding preferential substrate use.

Substrates that are less used by abundant skin taxa tend to be plant sugars, for example cellobiose [90], rhamnose [91], and xylose [92]. It is not difficult to understand why the ability to consume plant compounds provides little advantage on skin. Surprisingly, however, consumption of these sugars seems to be preferentially concentrated at moist sites, at least relative to sebaceous sites (see Additional file 1: Supplemental Information V, Figure S5.8 and S5.9). It is not obvious why there would be any benefit of plant sugar consumption in these regions. Urea use is also more common at moist sites (see Additional file 1: Supplemental Information IV, Figure S5.11), again for reasons that are unclear. In fact, urea use in general is surprising. Despite being prevalent on human skin [93], urea is one of the least commonly used substrates in our study (see Figs. 1 and 2). Why urea is not used by more skin bacteria, and why it seems to be used most at moist sites, highlights how trait-based analyses can uncover new, and unexpected trends, opening novel lines of inquiry that will ultimately help to elucidate factors governing skin microbiome composition.

### Comparison to ProTrait

Both our database and the ProTrait database [47] draw from a vast literature of culture-based experiments. Whereas we manually curate our data, the ProTrait database uses a text-mining algorithm. Not surprisingly, our database contains information on fewer bacterial species (971 vs. 3046, with 25 unique to our database). Coverage of traits, however, is similar. We include several enzymes and carbon sources (for example arylsulfatase, pyrazinamidase, tellurite reductase, caprate, itaconate, suberate, succinate, urocanate, valerate, 3-hydroxybutyric acid, 3-hydroxybenzoate, asparagine, ornithine, phenylalanine, proline, threonine, tryptophan, glucosamine, methyl-B-d-glucoside, butanol, xylitol, 2,3-butanediol, carnitine, phenethylamine, putrescine, thymidine, uridine, and 2-aminethanol) that are not in ProTrait; however, the ProTrait database contains other enzymes and substrates that are not in our database. Interestingly, there do not appear to be significant differences in error rates between the two databases, at least for traits whose values are specified. The databases do, however, substantially differ in trait coverage. In particular, our database specifies the values of traits for a greater number of organisms, whereas the ProTrait database is more likely to report traits as unknown, at least using a precision of ≥ 0.9 (see Supplemental Information VI for several example comparisons).

### Potential limitations

Our curated trait-based approach has many benefits, but also some draw-backs. First, we only consider well-defined taxa, ignoring detected taxa that have not been fully characterized, as well as all “dark matter” [59]. This could bias some of our predictions. While functional database methods are not as restricted in this way, they still rely on detection of orthologous genes. Consequently, both approaches are likely to miss at least some traits, particularly when these arise from poorly characterized taxonomic groups. Another complication of our approach is that it relies on conservation of functional traits within a species. Though our assumptions are likely less severe than tools like PICRUSt, functional traits are not always conserved. In compiling our database, we recorded evidence of strain variation, which suggested that interstrain differences in carbon source utilization are most common (14% of taxa), followed by differences in enzyme activity (11% of taxa). Although such variability complicates our analyses, it is more likely to obscure patterns than create them. Thus, when a pattern is detected, it likely reflects true biology.

## Conclusions

Many opportunities exist for increased trait-based analysis of microbiome communities. Future studies considering additional human and non-human environments will help elucidate the structuring principles and biological mechanisms driving patterns in worldwide microbial distributions. Meanwhile, extended analyses of skin microbiomes will further highlight the principles governing community assembly. Analyses that quantitatively account for microbial abundance, for example, could clarify differences among dry, moist, and sebaceous sites, while further gradation by body location is also possible. Another extension would be to consider functional trait differences between different people—something that would be particularly informative when comparing individuals with skin disease to healthy controls.

Trait-based analyses and functional comparisons are the next step in microbiome research. Although most studies attempting to do this have taken a functional database/pathway analysis approach, culture and lab-based studies afford unique benefits. Our analysis of the skin microbiome has elucidated some of these benefits, detecting different patterns than were observed using KEGG [59]. This, in turn, has opened up a range of questions about why specific microbes exist in certain skin environments, and what they are doing to survive.

## Materials and methods

### Species list for the human skin microbiome

We defined a list of skin bacterial species using a recent study [59] that employed shotgun sequencing (see Additional file 1: Supplemental Information I, Table S1.1). Specifically, whole genome shotgun data from the NCBI Sequence Read Archive (SRA) project SRP002480 was obtained from the SRA FTP site and converted to paired-end FASTQ format using the splitsra script in our Git repository hosted at: https://bitbucket.org/skinmicrobiome/metagenomics-scripts. FASTQ data originating from the same BioSample were consolidated into the same file using a custom shell script and the SRA RunInfo table found here: http://www.ncbi.nlm.nih.gov/Traces/study/?acc=SRP002480.

A reference database was constructed for the Kraken classifier [94] using the complete genomes in RefSeq for the bacterial (2199 taxonomic IDs), archaeal (165 taxonomic IDs), and viral (4011 taxonomic IDs) domains, as well as eight representative fungal taxonomic IDs, the *Plasmodium falciparum* 3D7 genome, the human genome, and the UniVec Core database (ftp://ftp.ncbi.nlm.nih.gov/pub/UniVec). Low complexity regions of the microbial reference sequences were masked using the dustmasker program with a DUST level of 20 [http://www.ncbi.nlm.nih.gov/pubmed/16796549]. After masking, every 31-mer nucleotide sequence present in the collection of reference FASTA sequences was stored at the taxonomic ID of the lowest common ancestor among the leaf nodes that share that 31-mer (see [94] for details). The total size of the database plus index was 110 GB.

Each input read from SRA project SRP002480 was assigned a taxonomic ID using Kraken by finding exact matches between every 31-mer nucleotide sequence present in that read and the database of 31-mers constructed above. Because of the hierarchical storage of k-mers in the database, reads can be classified at more general taxonomic levels than the specific strain sequences that were used to build the database. Output from the Kraken classification was summarized by taxonomic ID along with the number of unique k-mers detected in the data using the kraken-report-modif script (present in the metagenomics-scripts repository linked above). The total number of unique k-mers for each taxonomic ID in the database was obtained using the count_kmers.pl script, and full taxonomic strings were generated using the taxid2taxstring script, both included in the metagenomics-scripts git repository linked above.

Two separate lists were constructed from the above output (see Additional file 1: Supplemental Information I, Table S3.1). The first list, representing all human skin taxa, was determined by recording any species that occurred in at least one sample with a relative abundance > 0.001% of reads. We set a lower bound on the percentage of reads because taxa with only a handful of reads may be spurious and/or may represent incorrect taxonomic assignments. The second list, representing abundant skin taxa, was determined by recording any species that occurred in at least one sample with a relative abundance of 0.1% of reads. We chose to consider abundance classes (all taxa vs. abundant taxa), rather than specifically accounting for abundance because abundance estimation from shotgun sequencing data is notoriously difficult.

### Skin database compilation

Using the lists of taxa generated above, we compiled a database of microbial traits. For this, we relied on *Bergey*’*s Manual of Systematic Bacteriology* [42–45] and the initial journal articles describing each species. We only considered validly described species and did not include *Candidatus* taxa, since little information was available for these. Our database contains information for 971 species.

### World database compilation

We used a database compiled from species descriptions in the *International Journal of Systematic and Evolutionary Microbiology.* A full description of this database, including its availability, can be found at [48] (see also, Additional file 1: Supplemental Information I, Table S1.2).

### Statistical analyses

Depending on the variable, we performed three types of comparisons: binary, categorical, and quantitative, across two sets of contrasts: skin vs. world and within skin bacteria, among the three skin environments: dry, moist, and sebaceous. These comparisons were conducted across all *Bacteria* and the four major phyla, separately considering abundant (> 0.1% of reads) and all taxa (> 0.001% of reads) respectively.

Binary comparisons were performed on variables that had two outcomes (e.g., positive and negative). When making two-way binary comparisons, we estimated proportion of occurrence with standard errors using a standard binomial model. For an overall test of difference in proportion, we used a Chi-square test. Pairwise comparisons were made using the standard errors of the binomial proportion. We visualized the comparisons with scatter plots of point estimates and error bars, using the 45° equality line as a guide for relative prevalence of the variables.

Categorical comparisons were performed on variables with multiple discrete, unordered outcomes (e.g., chain, clump, or singly). We compared the relative frequencies of the different outcomes in skin vs. world (or pairwise across skin environments) using a randomization test in which we resampled the data 10^5^ times and computed a *p* value for the null hypothesis of equality of proportions by computing the number of randomized samples that were less extreme than the observed proportion.

Quantitative outcomes (e.g., volume, pH tolerance) were compared using a nonparametric relative effects test based on global rankings using Tukey contrasts [95]. We chose this test because it is robust to highly non-normal distributions and non-uniform variances and controls appropriately for multiple comparisons. We used box-and-whisker plots of each variable for visualization of the medians and deviations in the data.

Finally, to explore the role of phylogenetic conservation as an explanation for observed trends, for all binary and qualitative traits, we regressed the probability of a taxon being abundant versus rare or being from skin versus the world against each trait individually, both for a naïve logistic regression and for a regression where phylogenetic relatedness was accounted for. For the latter, we used the phylolm package in R [56] and the phylogenetic tree from Yarza et al. [96]. A handful of taxa were missing from the tree, and these were ignored in subsequent analysis. To test the overall significance of a fitted regression, we compared the regression to a null model using a likelihood ratio test. We then compared *p*-values for the naïve logistic regression and the regression with phylogenetic correction.

All statistical analysis was performed using the R programming language (R Code Team 2016), with the quantitative analysis performed using the nparcomp package [95].

## Additional file

Additional file 1: Supplementary Information I through VI, containing information on the dataset and database, phylogenetically corrected analyses, additional analyses of full microbiomes, including rare members, body-site comparisons, and a comparison to ProTrait. (DOCX 43176 kb)

## Footnotes

1 Note that we have ignored several compounds (e.g., carnitine, phenylethylamine, methyl-pyruvate) where results were only reported for a handful (< 20) of species.
