# Supplementary material for: Trait-based analysis of the human skin microbiome
Source: Microbiome. 2019 Jul 5;7:101. doi: 10.1186/s40168-019-0698-2 (PMC6612184; doi:10.1186/s40168-019-0698-2)
Supplement: Supplementary file 1 — Supplementary Information I through VI, containing information on the dataset and database, phylogenetically corrected analyses, additional analyses of full microbiomes, including rare members, body-site comparisons, and a comparison to ProTrait. (DOCX 43176 kb) [file 40168_2019_698_MOESM1_ESM.docx]

Supplementary Information

*Supplementary Information I: Summary of Datasets and Databases*

Table S1.1 identifies the various body sites classified as dry, moist and sebaceous respectively, along with the number of samples from each site (56). Table S1.2 shows the number of taxa in our world database, and in our lists of abundant and all skin bacteria for the entire Bacterial kingdom, as well as for the four dominant phyla found on human skin.

**Table S1.1** Dry, moist and sebaceous skin sites

used in this study.

| **dry** | **no. samples** |
| --- | --- |
| hypothenar palm | 16 |
| volar forearm | 13 |
| **moist** | **no. samples** |
| antecubital fossa | 15 |
| axilla | 2 |
| inguinal crease | 15 |
| interdigital web space | 11 |
| nares | 11 |
| popliteal crease | 15 |
| plantar heel | 17 |
| toe web | 16 |
| **sebaceous** | **no. samples** |
| alar crease | 17 |
| back | 13 |
| cheek | 16 |
| external auditory canal | 15 |
| glabella | 14 |
| manubrium | 14 |
| occiput | 15 |
| retroauricular crease | 16 |

.

**Table S1.2** Numbers of bacterial taxa overall and in each of the dominant four phyla in our world database, and amongst abundant (0.1% of reads in at least one sample) and all (0.001% of reads in at least one sample) bacteria on human skin.

| **Category** | **Bacteria** | **Actinobacteria** | **Firmicutes** | **Proteobacteria** | **Bacteroidetes** |
| --- | --- | --- | --- | --- | --- |
| world | 4702 | 915 | 699 | 1485 | 621 |
| skin (abundant) | 971 | 139 | 211 | 406 | 64 |
| skin (all) | 180 | 37 | 53 | 73 | 12 |

**Figure S1.1** Species diversity within each genus for (A) abundant skin taxa, (B) all skin taxa and (C) the world database

**Figure S1.2** Species diversity within each family for (A) abundant skin taxa, (B) all skin taxa and (C) the world database

**Figure S1.3** Species diversity within each order for (A) abundant skin taxa, (B) all skin taxa and (C) the world database

**Table S1.3** List of abundant skin taxa (present at >0.1% of reads in at least one sample)

| **Phylum** | **Class** | **Order** | **Family** | **Genus/Species** |
| --- | --- | --- | --- | --- |
| Actinobacteria | Actinobacteria | Actinomycetales | Actinomycetaceae | Mobiluncus curtisii |
| Actinobacteria | Actinobacteria | Actinomycetales | Corynebacteriaceae | Corynebacterium argentoratense |
| Actinobacteria | Actinobacteria | Actinomycetales | Corynebacteriaceae | Corynebacterium aurimucosum |
| Actinobacteria | Actinobacteria | Actinomycetales | Corynebacteriaceae | Corynebacterium callunae |
| Actinobacteria | Actinobacteria | Actinomycetales | Corynebacteriaceae | Corynebacterium diphtheriae |
| Actinobacteria | Actinobacteria | Actinomycetales | Corynebacteriaceae | Corynebacterium efficiens |
| Actinobacteria | Actinobacteria | Actinomycetales | Corynebacteriaceae | Corynebacterium glutamicum |
| Actinobacteria | Actinobacteria | Actinomycetales | Corynebacteriaceae | Corynebacterium halotolerans |
| Actinobacteria | Actinobacteria | Actinomycetales | Corynebacteriaceae | Corynebacterium jeikeium |
| Actinobacteria | Actinobacteria | Actinomycetales | Corynebacteriaceae | Corynebacterium kroppenstedtii |
| Actinobacteria | Actinobacteria | Actinomycetales | Corynebacteriaceae | Corynebacterium maris |
| Actinobacteria | Actinobacteria | Actinomycetales | Corynebacteriaceae | Corynebacterium resistens |
| Actinobacteria | Actinobacteria | Actinomycetales | Corynebacteriaceae | Corynebacterium terpenotabidum |
| Actinobacteria | Actinobacteria | Actinomycetales | Corynebacteriaceae | Corynebacterium urealyticum |
| Actinobacteria | Actinobacteria | Actinomycetales | Corynebacteriaceae | Corynebacterium variabile |
| Actinobacteria | Actinobacteria | Actinomycetales | Gordoniaceae | Gordonia bronchialis |
| Actinobacteria | Actinobacteria | Actinomycetales | Gordoniaceae | Gordonia polyisoprenivorans |
| Actinobacteria | Actinobacteria | Actinomycetales | Nocardiaceae | Rhodococcus erythropolis |
| Actinobacteria | Actinobacteria | Actinomycetales | Geodermatophilaceae | Modestobacter marinus |
| Actinobacteria | Actinobacteria | Actinomycetales | Dermabacteraceae | Brachybacterium faecium |
| Actinobacteria | Actinobacteria | Actinomycetales | Dermacoccaceae | Kytococcus sedentarius |
| Actinobacteria | Actinobacteria | Actinomycetales | Microbacteriaceae | Clavibacter michiganensis |
| Actinobacteria | Actinobacteria | Actinomycetales | Microbacteriaceae | Microbacterium testaceum |
| Actinobacteria | Actinobacteria | Actinomycetales | Micrococcaceae | Arthrobacter arilaitensis |
| Actinobacteria | Actinobacteria | Actinomycetales | Micrococcaceae | Arthrobacter chlorophenolicus |
| Actinobacteria | Actinobacteria | Actinomycetales | Micrococcaceae | Arthrobacter phenanthrenivorans |
| Actinobacteria | Actinobacteria | Actinomycetales | Micrococcaceae | Kocuria rhizophila |
| Actinobacteria | Actinobacteria | Actinomycetales | Micrococcaceae | Micrococcus luteus |
| Actinobacteria | Actinobacteria | Actinomycetales | Micrococcaceae | Rothia dentocariosa |
| Actinobacteria | Actinobacteria | Actinomycetales | Micrococcaceae | Rothia mucilaginosa |
| Actinobacteria | Actinobacteria | Actinomycetales | Propionibacteriaceae | Propionibacterium acidipropionici |
| Actinobacteria | Actinobacteria | Actinomycetales | Propionibacteriaceae | Propionibacterium acnes |
| Actinobacteria | Actinobacteria | Actinomycetales | Propionibacteriaceae | Propionibacterium avidum |
| Actinobacteria | Actinobacteria | Actinomycetales | Propionibacteriaceae | Propionibacterium propionicum |
| Actinobacteria | Actinobacteria | Bifidobacteriales | Bifidobacteriaceae | Bifidobacterium adolescentis |
| Actinobacteria | Actinobacteria | Bifidobacteriales | Bifidobacteriaceae | Bifidobacterium longum |
| Actinobacteria | Actinobacteria | Bifidobacteriales | Bifidobacteriaceae | Gardnerella vaginalis |
| Bacteroidetes | Bacteroidia | Bacteroidales | Bacteroidaceae | Bacteroides fragilis |
| Bacteroidetes | Bacteroidia | Bacteroidales | Bacteroidaceae | Bacteroides vulgatus |
| Bacteroidetes | Bacteroidia | Bacteroidales | Bacteroidaceae | Bacteroides xylanisolvens |
| Bacteroidetes | Bacteroidia | Bacteroidales | Porphyromonadaceae | Parabacteroides distasonis |
| Bacteroidetes | Bacteroidia | Bacteroidales | Porphyromonadaceae | Porphyromonas asaccharolytica |
| Bacteroidetes | Bacteroidia | Bacteroidales | Porphyromonadaceae | Porphyromonas gingivalis |
| Bacteroidetes | Bacteroidia | Bacteroidales | Prevotellaceae | Prevotella dentalis |
| Bacteroidetes | Bacteroidia | Bacteroidales | Prevotellaceae | Prevotella denticola |
| Bacteroidetes | Bacteroidia | Bacteroidales | Prevotellaceae | Prevotella intermedia |
| Bacteroidetes | Bacteroidia | Bacteroidales | Prevotellaceae | Prevotella melaninogenica |
| Bacteroidetes | Flavobacteriia | Flavobacteriales | Flavobacteriaceae | Capnocytophaga ochracea |
| Bacteroidetes | Flavobacteriia | Flavobacteriales | Flavobacteriaceae | Weeksella virosa |
| Deinococcus-Thermus | Deinococci | Deinococcales | Deinococcaceae | Deinococcus geothermalis |
| Deinococcus-Thermus | Deinococci | Deinococcales | Deinococcaceae | Deinococcus proteolyticus |
| Deinococcus-Thermus | Deinococci | Thermales | Thermaceae | Thermus scotoductus |
| Firmicutes | Bacilli | Bacillales | Bacillaceae | Bacillus cereus |
| Firmicutes | Bacilli | Bacillales | Bacillaceae | Bacillus licheniformis |
| Firmicutes | Bacilli | Bacillales | Bacillaceae | Bacillus subtilis |
| Firmicutes | Bacilli | Bacillales | Staphylococcaceae | Staphylococcus aureus |
| Firmicutes | Bacilli | Bacillales | Staphylococcaceae | Staphylococcus carnosus |
| Firmicutes | Bacilli | Bacillales | Staphylococcaceae | Staphylococcus epidermidis |
| Firmicutes | Bacilli | Bacillales | Staphylococcaceae | Staphylococcus haemolyticus |
| Firmicutes | Bacilli | Bacillales | Staphylococcaceae | Staphylococcus lugdunensis |
| Firmicutes | Bacilli | Bacillales | Staphylococcaceae | Staphylococcus pasteuri |
| Firmicutes | Bacilli | Bacillales | Staphylococcaceae | Staphylococcus pseudintermedius |
| Firmicutes | Bacilli | Bacillales | Staphylococcaceae | Staphylococcus saprophyticus |
| Firmicutes | Bacilli | Bacillales | Staphylococcaceae | Staphylococcus warneri |
| Firmicutes | Bacilli | Lactobacillales | Carnobacteriaceae | Carnobacterium maltaromaticum |
| Firmicutes | Bacilli | Lactobacillales | Enterococcaceae | Enterococcus faecalis |
| Firmicutes | Bacilli | Lactobacillales | Enterococcaceae | Tetragenococcus halophilus |
| Firmicutes | Bacilli | Lactobacillales | Lactobacillaceae | Lactobacillus acidophilus |
| Firmicutes | Bacilli | Lactobacillales | Lactobacillaceae | Lactobacillus amylovorus |
| Firmicutes | Bacilli | Lactobacillales | Lactobacillaceae | Lactobacillus buchneri |
| Firmicutes | Bacilli | Lactobacillales | Lactobacillaceae | Lactobacillus crispatus |
| Firmicutes | Bacilli | Lactobacillales | Lactobacillaceae | Lactobacillus gasseri |
| Firmicutes | Bacilli | Lactobacillales | Lactobacillaceae | Lactobacillus helveticus |
| Firmicutes | Bacilli | Lactobacillales | Lactobacillaceae | Lactobacillus johnsonii |
| Firmicutes | Bacilli | Lactobacillales | Lactobacillaceae | Lactobacillus kefiranofaciens |
| Firmicutes | Bacilli | Lactobacillales | Lactobacillaceae | Lactobacillus reuteri |
| Firmicutes | Bacilli | Lactobacillales | Lactobacillaceae | Lactobacillus salivarius |
| Firmicutes | Bacilli | Lactobacillales | Leuconostocaceae | Leuconostoc mesenteroides |
| Firmicutes | Bacilli | Lactobacillales | Streptococcaceae | Lactococcus lactis |
| Firmicutes | Bacilli | Lactobacillales | Streptococcaceae | Streptococcus agalactiae |
| Firmicutes | Bacilli | Lactobacillales | Streptococcaceae | Streptococcus anginosus |
| Firmicutes | Bacilli | Lactobacillales | Streptococcaceae | Streptococcus intermedius |
| Firmicutes | Bacilli | Lactobacillales | Streptococcaceae | Streptococcus dysgalactiae |
| Firmicutes | Bacilli | Lactobacillales | Streptococcaceae | Streptococcus gordonii |
| Firmicutes | Bacilli | Lactobacillales | Streptococcaceae | Streptococcus mitis |
| Firmicutes | Bacilli | Lactobacillales | Streptococcaceae | Streptococcus mutans |
| Firmicutes | Bacilli | Lactobacillales | Streptococcaceae | Streptococcus oligofermentans |
| Firmicutes | Bacilli | Lactobacillales | Streptococcaceae | Streptococcus oralis |
| Firmicutes | Bacilli | Lactobacillales | Streptococcaceae | Streptococcus parasanguinis |
| Firmicutes | Bacilli | Lactobacillales | Streptococcaceae | Streptococcus pneumoniae |
| Firmicutes | Bacilli | Lactobacillales | Streptococcaceae | Streptococcus pseudopneumoniae |
| Firmicutes | Bacilli | Lactobacillales | Streptococcaceae | Streptococcus pyogenes |
| Firmicutes | Bacilli | Lactobacillales | Streptococcaceae | Streptococcus salivarius |
| Firmicutes | Bacilli | Lactobacillales | Streptococcaceae | Streptococcus sanguinis |
| Firmicutes | Bacilli | Lactobacillales | Streptococcaceae | Streptococcus thermophilus |
| Firmicutes | Clostridia | Clostridiales | Clostridiaceae | Clostridium beijerinckii |
| Firmicutes | Clostridia | Clostridiales | Eubacteriaceae | Eubacterium rectale |
| Firmicutes | Clostridia | Clostridiales | Lachnospiraceae | Ruminococcus obeum |
| Firmicutes | Clostridia | Clostridiales | Lachnospiraceae | Ruminococcus torques |
| Firmicutes | Clostridia | Clostridiales | Peptoniphilaceae | Anaerococcus prevotii |
| Firmicutes | Clostridia | Clostridiales | Peptoniphilaceae | Finegoldia magna |
| Firmicutes | Clostridia | Clostridiales | Peptostreptococcaceae | Filifactor alocis |
| Firmicutes | Clostridia | Clostridiales | Ruminococcaceae | Faecalibacterium prausnitzii |
| Firmicutes | Clostridia | Clostridiales | Ruminococcaceae | Ruminococcus bromii |
| Firmicutes | Negativicutes | Selenomonadales | Veillonellaceae | Veillonella parvula |
| Fusobacteria | Fusobacteriia | Fusobacteriales | Fusobacteriaceae | Fusobacterium nucleatum |
| Fusobacteria | Fusobacteriia | Fusobacteriales | Leptotrichiaceae | Leptotrichia buccalis |
| Proteobacteria | Alphaproteobacteria | Caulobacterales | Caulobacteraceae | Brevundimonas subvibrioides |
| Proteobacteria | Alphaproteobacteria | Caulobacterales | Caulobacteraceae | Caulobacter segnis |
| Proteobacteria | Alphaproteobacteria | Caulobacterales | Caulobacteraceae | Caulobacter vibrioides |
| Proteobacteria | Alphaproteobacteria | Caulobacterales | Caulobacteraceae | Phenylobacterium zucineum |
| Proteobacteria | Alphaproteobacteria | Rhizobiales | Brucellaceae | Ochrobactrum anthropi |
| Proteobacteria | Alphaproteobacteria | Rhizobiales | Methylobacteriaceae | Methylobacterium populi |
| Proteobacteria | Alphaproteobacteria | Rhizobiales | Methylobacteriaceae | Methylobacterium radiotolerans |
| Proteobacteria | Alphaproteobacteria | Rhodobacterales | Rhodobacteraceae | Dinoroseobacter shibae |
| Proteobacteria | Alphaproteobacteria | Rhodobacterales | Rhodobacteraceae | Paracoccus aminophilus |
| Proteobacteria | Alphaproteobacteria | Rhodobacterales | Rhodobacteraceae | Paracoccus denitrificans |
| Proteobacteria | Alphaproteobacteria | Rhodobacterales | Rhodobacteraceae | Rhodobacter capsulatus |
| Proteobacteria | Alphaproteobacteria | Rhodobacterales | Rhodobacteraceae | Rhodobacter sphaeroides |
| Proteobacteria | Alphaproteobacteria | Rhodobacterales | Rhodobacteraceae | Ruegeria pomeroyi |
| Proteobacteria | Alphaproteobacteria | Sphingomonadales | Sphingomonadaceae | Novosphingobium aromaticivorans |
| Proteobacteria | Alphaproteobacteria | Sphingomonadales | Sphingomonadaceae | Sphingobium japonicum |
| Proteobacteria | Alphaproteobacteria | Sphingomonadales | Sphingomonadaceae | Sphingomonas wittichii |
| Proteobacteria | Alphaproteobacteria | Sphingomonadales | Sphingomonadaceae | Sphingopyxis alaskensis |
| Proteobacteria | Betaproteobacteria | Burkholderiales | Alcaligenaceae | Achromobacter xylosoxidans |
| Proteobacteria | Betaproteobacteria | Burkholderiales | Comamonadaceae | Alicycliphilus denitrificans |
| Proteobacteria | Betaproteobacteria | Burkholderiales | Comamonadaceae | Comamonas testosteroni |
| Proteobacteria | Betaproteobacteria | Burkholderiales | Comamonadaceae | Delftia acidovorans |
| Proteobacteria | Betaproteobacteria | Burkholderiales | Comamonadaceae | Variovorax paradoxus |
| Proteobacteria | Betaproteobacteria | Burkholderiales | Oxalobacteraceae | Herbaspirillum seropedicae |
| Proteobacteria | Betaproteobacteria | Burkholderiales |  | Methylibium petroleiphilum |
| Proteobacteria | Betaproteobacteria | Neisseriales | Neisseriaceae | Neisseria gonorrhoeae |
| Proteobacteria | Betaproteobacteria | Neisseriales | Neisseriaceae | Neisseria lactamica |
| Proteobacteria | Betaproteobacteria | Neisseriales | Neisseriaceae | Neisseria meningitidis |
| Proteobacteria | Betaproteobacteria | Rhodocyclales | Rhodocyclaceae | Azospira oryzae |
| Proteobacteria | Epsilonproteobacteria | Campylobacterales | Campylobacteraceae | Campylobacter concisus |
| Proteobacteria | Epsilonproteobacteria | Campylobacterales | Campylobacteraceae | Campylobacter hominis |
| Proteobacteria | Epsilonproteobacteria | Campylobacterales | Helicobacteraceae | Helicobacter pylori |
| Proteobacteria | Gammaproteobacteria | Aeromonadales | Aeromonadaceae | Aeromonas hydrophila |
| Proteobacteria | Gammaproteobacteria | Aeromonadales | Aeromonadaceae | Aeromonas salmonicida |
| Proteobacteria | Gammaproteobacteria | Aeromonadales | Aeromonadaceae | Aeromonas veronii |
| Proteobacteria | Gammaproteobacteria | Alteromonadales | Shewanellaceae | Shewanella baltica |
| Proteobacteria | Gammaproteobacteria | Alteromonadales | Shewanellaceae | Shewanella oneidensis |
| Proteobacteria | Gammaproteobacteria | Enterobacteriales | Enterobacteriaceae | Citrobacter koseri |
| Proteobacteria | Gammaproteobacteria | Enterobacteriales | Enterobacteriaceae | Cronobacter sakazakii |
| Proteobacteria | Gammaproteobacteria | Enterobacteriales | Enterobacteriaceae | Enterobacter asburiae |
| Proteobacteria | Gammaproteobacteria | Enterobacteriales | Enterobacteriaceae | Enterobacter cloacae |
| Proteobacteria | Gammaproteobacteria | Enterobacteriales | Enterobacteriaceae | Escherichia coli |
| Proteobacteria | Gammaproteobacteria | Enterobacteriales | Enterobacteriaceae | Klebsiella oxytoca |
| Proteobacteria | Gammaproteobacteria | Enterobacteriales | Enterobacteriaceae | Klebsiella pneumoniae |
| Proteobacteria | Gammaproteobacteria | Enterobacteriales | Enterobacteriaceae | Klebsiella variicola |
| Proteobacteria | Gammaproteobacteria | Enterobacteriales | Enterobacteriaceae | Pantoea ananatis |
| Proteobacteria | Gammaproteobacteria | Enterobacteriales | Enterobacteriaceae | Pantoea vagans |
| Proteobacteria | Gammaproteobacteria | Enterobacteriales | Enterobacteriaceae | Raoultella ornithinolytica |
| Proteobacteria | Gammaproteobacteria | Enterobacteriales | Enterobacteriaceae | Salmonella enterica |
| Proteobacteria | Gammaproteobacteria | Enterobacteriales | Enterobacteriaceae | Serratia liquefaciens |
| Proteobacteria | Gammaproteobacteria | Enterobacteriales | Enterobacteriaceae | Serratia marcescens |
| Proteobacteria | Gammaproteobacteria | Pasteurellales | Pasteurellaceae | Aggregatibacter aphrophilus |
| Proteobacteria | Gammaproteobacteria | Pasteurellales | Pasteurellaceae | Haemophilus influenzae |
| Proteobacteria | Gammaproteobacteria | Pasteurellales | Pasteurellaceae | Haemophilus parainfluenzae |
| Proteobacteria | Gammaproteobacteria | Pseudomonadales | Moraxellaceae | Acinetobacter baumannii |
| Proteobacteria | Gammaproteobacteria | Pseudomonadales | Moraxellaceae | Acinetobacter calcoaceticus |
| Proteobacteria | Gammaproteobacteria | Pseudomonadales | Moraxellaceae | Acinetobacter oleivorans |
| Proteobacteria | Gammaproteobacteria | Pseudomonadales | Pseudomonadaceae | Pseudomonas aeruginosa |
| Proteobacteria | Gammaproteobacteria | Pseudomonadales | Pseudomonadaceae | Pseudomonas mendocina |
| Proteobacteria | Gammaproteobacteria | Pseudomonadales | Pseudomonadaceae | Pseudomonas resinovorans |
| Proteobacteria | Gammaproteobacteria | Pseudomonadales | Pseudomonadaceae | Pseudomonas brassicacearum |
| Proteobacteria | Gammaproteobacteria | Pseudomonadales | Pseudomonadaceae | Pseudomonas entomophila |
| Proteobacteria | Gammaproteobacteria | Pseudomonadales | Pseudomonadaceae | Pseudomonas fluorescens |
| Proteobacteria | Gammaproteobacteria | Pseudomonadales | Pseudomonadaceae | Pseudomonas poae |
| Proteobacteria | Gammaproteobacteria | Pseudomonadales | Pseudomonadaceae | Pseudomonas protegens |
| Proteobacteria | Gammaproteobacteria | Pseudomonadales | Pseudomonadaceae | Pseudomonas fulva |
| Proteobacteria | Gammaproteobacteria | Pseudomonadales | Pseudomonadaceae | Pseudomonas putida |
| Proteobacteria | Gammaproteobacteria | Pseudomonadales | Pseudomonadaceae | Pseudomonas stutzeri |
| Proteobacteria | Gammaproteobacteria | Pseudomonadales | Pseudomonadaceae | Pseudomonas syringae |
| Proteobacteria | Gammaproteobacteria | Pseudomonadales | Pseudomonadaceae | Pseudomonas savastanoi |
| Proteobacteria | Gammaproteobacteria | Xanthomonadales | Xanthomonadaceae | Pseudoxanthomonas spadix |
| Proteobacteria | Gammaproteobacteria | Xanthomonadales | Xanthomonadaceae | Pseudoxanthomonas suwonensis |
| Proteobacteria | Gammaproteobacteria | Xanthomonadales | Xanthomonadaceae | Stenotrophomonas maltophilia |
| Proteobacteria | Gammaproteobacteria | Xanthomonadales | Xanthomonadaceae | Xanthomonas campestris |

**Table S1.4** List of all skin taxa (present at >0.001% of reads in at least one sample)

| **Phylum** | **Class** | **Order** | **Family** | **Genus/Species** |
| --- | --- | --- | --- | --- |
| Actinobacteria | Actinobacteria | Acidimicrobiales | Acidimicrobiaceae | Acidimicrobium ferrooxidans |
| Actinobacteria | Actinobacteria | Acidimicrobiales | Acidimicrobiaceae | Ilumatobacter coccineus |
| Actinobacteria | Actinobacteria | Actinomycetales | Actinomycetaceae | Arcanobacterium haemolyticum |
| Actinobacteria | Actinobacteria | Actinomycetales | Actinomycetaceae | Mobiluncus curtisii |
| Actinobacteria | Actinobacteria | Actinomycetales | Catenulisporaceae | Catenulispora acidiphila |
| Actinobacteria | Actinobacteria | Actinomycetales | Corynebacteriaceae | Corynebacterium argentoratense |
| Actinobacteria | Actinobacteria | Actinomycetales | Corynebacteriaceae | Corynebacterium aurimucosum |
| Actinobacteria | Actinobacteria | Actinomycetales | Corynebacteriaceae | Corynebacterium callunae |
| Actinobacteria | Actinobacteria | Actinomycetales | Corynebacteriaceae | Corynebacterium diphtheriae |
| Actinobacteria | Actinobacteria | Actinomycetales | Corynebacteriaceae | Corynebacterium efficiens |
| Actinobacteria | Actinobacteria | Actinomycetales | Corynebacteriaceae | Corynebacterium glutamicum |
| Actinobacteria | Actinobacteria | Actinomycetales | Corynebacteriaceae | Corynebacterium halotolerans |
| Actinobacteria | Actinobacteria | Actinomycetales | Corynebacteriaceae | Corynebacterium jeikeium |
| Actinobacteria | Actinobacteria | Actinomycetales | Corynebacteriaceae | Corynebacterium kroppenstedtii |
| Actinobacteria | Actinobacteria | Actinomycetales | Corynebacteriaceae | Corynebacterium maris |
| Actinobacteria | Actinobacteria | Actinomycetales | Corynebacteriaceae | Corynebacterium pseudotuberculosis |
| Actinobacteria | Actinobacteria | Actinomycetales | Corynebacteriaceae | Corynebacterium resistens |
| Actinobacteria | Actinobacteria | Actinomycetales | Corynebacteriaceae | Corynebacterium terpenotabidum |
| Actinobacteria | Actinobacteria | Actinomycetales | Corynebacteriaceae | Corynebacterium ulcerans |
| Actinobacteria | Actinobacteria | Actinomycetales | Corynebacteriaceae | Corynebacterium urealyticum |
| Actinobacteria | Actinobacteria | Actinomycetales | Corynebacteriaceae | Corynebacterium variabile |
| Actinobacteria | Actinobacteria | Actinomycetales | Gordoniaceae | Gordonia bronchialis |
| Actinobacteria | Actinobacteria | Actinomycetales | Gordoniaceae | Gordonia polyisoprenivorans |
| Actinobacteria | Actinobacteria | Actinomycetales | Mycobacteriaceae | Amycolicicoccus subflavus |
| Actinobacteria | Actinobacteria | Actinomycetales | Mycobacteriaceae | Mycobacterium avium |
| Actinobacteria | Actinobacteria | Actinomycetales | Mycobacteriaceae | Mycobacterium indicus pranii |
| Actinobacteria | Actinobacteria | Actinomycetales | Mycobacteriaceae | Mycobacterium intracellulare |
| Actinobacteria | Actinobacteria | Actinomycetales | Mycobacteriaceae | Mycobacterium yongonense |
| Actinobacteria | Actinobacteria | Actinomycetales | Mycobacteriaceae | Mycobacterium abscessus |
| Actinobacteria | Actinobacteria | Actinomycetales | Mycobacteriaceae | Mycobacterium chubuense |
| Actinobacteria | Actinobacteria | Actinomycetales | Mycobacteriaceae | Mycobacterium gilvum |
| Actinobacteria | Actinobacteria | Actinomycetales | Mycobacteriaceae | Mycobacterium kansasii |
| Actinobacteria | Actinobacteria | Actinomycetales | Mycobacteriaceae | Mycobacterium leprae |
| Actinobacteria | Actinobacteria | Actinomycetales | Mycobacteriaceae | Mycobacterium marinum |
| Actinobacteria | Actinobacteria | Actinomycetales | Mycobacteriaceae | Mycobacterium neoaurum |
| Actinobacteria | Actinobacteria | Actinomycetales | Mycobacteriaceae | Mycobacterium rhodesiae |
| Actinobacteria | Actinobacteria | Actinomycetales | Mycobacteriaceae | Mycobacterium smegmatis |
| Actinobacteria | Actinobacteria | Actinomycetales | Mycobacteriaceae | Mycobacterium tuberculosis |
| Actinobacteria | Actinobacteria | Actinomycetales | Mycobacteriaceae | Mycobacterium vanbaalenii |
| Actinobacteria | Actinobacteria | Actinomycetales | Nocardiaceae | Nocardia brasiliensis |
| Actinobacteria | Actinobacteria | Actinomycetales | Nocardiaceae | Nocardia cyriacigeorgica |
| Actinobacteria | Actinobacteria | Actinomycetales | Nocardiaceae | Nocardia farcinica |
| Actinobacteria | Actinobacteria | Actinomycetales | Nocardiaceae | Rhodococcus equi |
| Actinobacteria | Actinobacteria | Actinomycetales | Nocardiaceae | Rhodococcus erythropolis |
| Actinobacteria | Actinobacteria | Actinomycetales | Nocardiaceae | Rhodococcus jostii |
| Actinobacteria | Actinobacteria | Actinomycetales | Nocardiaceae | Rhodococcus opacus |
| Actinobacteria | Actinobacteria | Actinomycetales | Nocardiaceae | Rhodococcus pyridinivorans |
| Actinobacteria | Actinobacteria | Actinomycetales | Segniliparaceae | Segniliparus rotundus |
| Actinobacteria | Actinobacteria | Actinomycetales | Tsukamurellaceae | Tsukamurella paurometabola |
| Actinobacteria | Actinobacteria | Actinomycetales | Acidothermaceae | Acidothermus cellulolyticus |
| Actinobacteria | Actinobacteria | Actinomycetales | Frankiaceae | Frankia alni |
| Actinobacteria | Actinobacteria | Actinomycetales | Geodermatophilaceae | Blastococcus saxobsidens |
| Actinobacteria | Actinobacteria | Actinomycetales | Geodermatophilaceae | Geodermatophilus obscurus |
| Actinobacteria | Actinobacteria | Actinomycetales | Geodermatophilaceae | Modestobacter marinus |
| Actinobacteria | Actinobacteria | Actinomycetales | Nakamurellaceae | Nakamurella multipartita |
| Actinobacteria | Actinobacteria | Actinomycetales | Glycomycetaceae | Stackebrandtia nassauensis |
| Actinobacteria | Actinobacteria | Actinomycetales | Kineosporiaceae | Kineococcus radiotolerans |
| Actinobacteria | Actinobacteria | Actinomycetales | Beutenbergiaceae | Beutenbergia cavernae |
| Actinobacteria | Actinobacteria | Actinomycetales | Cellulomonadaceae | Cellulomonas fimi |
| Actinobacteria | Actinobacteria | Actinomycetales | Cellulomonadaceae | Cellulomonas flavigena |
| Actinobacteria | Actinobacteria | Actinomycetales | Cellulomonadaceae | Cellvibrio gilvus |
| Actinobacteria | Actinobacteria | Actinomycetales | Dermabacteraceae | Brachybacterium faecium |
| Actinobacteria | Actinobacteria | Actinomycetales | Dermacoccaceae | Kytococcus sedentarius |
| Actinobacteria | Actinobacteria | Actinomycetales | Intrasporangiaceae | Intrasporangium calvum |
| Actinobacteria | Actinobacteria | Actinomycetales | Jonesiaceae | Jonesia denitrificans |
| Actinobacteria | Actinobacteria | Actinomycetales | Microbacteriaceae | Clavibacter michiganensis |
| Actinobacteria | Actinobacteria | Actinomycetales | Microbacteriaceae | Leifsonia xyli |
| Actinobacteria | Actinobacteria | Actinomycetales | Microbacteriaceae | Microbacterium testaceum |
| Actinobacteria | Actinobacteria | Actinomycetales | Micrococcaceae | Arthrobacter arilaitensis |
| Actinobacteria | Actinobacteria | Actinomycetales | Micrococcaceae | Arthrobacter aurescens |
| Actinobacteria | Actinobacteria | Actinomycetales | Micrococcaceae | Arthrobacter chlorophenolicus |
| Actinobacteria | Actinobacteria | Actinomycetales | Micrococcaceae | Arthrobacter phenanthrenivorans |
| Actinobacteria | Actinobacteria | Actinomycetales | Micrococcaceae | Kocuria rhizophila |
| Actinobacteria | Actinobacteria | Actinomycetales | Micrococcaceae | Micrococcus luteus |
| Actinobacteria | Actinobacteria | Actinomycetales | Micrococcaceae | Renibacterium salmoninarum |
| Actinobacteria | Actinobacteria | Actinomycetales | Micrococcaceae | Rothia dentocariosa |
| Actinobacteria | Actinobacteria | Actinomycetales | Micrococcaceae | Rothia mucilaginosa |
| Actinobacteria | Actinobacteria | Actinomycetales | Promicromonosporaceae | Isoptericola variabilis |
| Actinobacteria | Actinobacteria | Actinomycetales | Promicromonosporaceae | Xylanimonas cellulosilytica |
| Actinobacteria | Actinobacteria | Actinomycetales | Sanguibacteraceae | Sanguibacter keddieii |
| Actinobacteria | Actinobacteria | Actinomycetales |  | Tropheryma whipplei |
| Actinobacteria | Actinobacteria | Actinomycetales | Micromonosporaceae | Actinoplanes friuliensis |
| Actinobacteria | Actinobacteria | Actinomycetales | Micromonosporaceae | Actinoplanes missouriensis |
| Actinobacteria | Actinobacteria | Actinomycetales | Micromonosporaceae | Micromonospora aurantiaca |
| Actinobacteria | Actinobacteria | Actinomycetales | Micromonosporaceae | Salinispora arenicola |
| Actinobacteria | Actinobacteria | Actinomycetales | Micromonosporaceae | Salinispora tropica |
| Actinobacteria | Actinobacteria | Actinomycetales | Micromonosporaceae | Verrucosispora maris |
| Actinobacteria | Actinobacteria | Actinomycetales | Nocardioidaceae | Kribbella flavida |
| Actinobacteria | Actinobacteria | Actinomycetales | Propionibacteriaceae | Microlunatus phosphovorus |
| Actinobacteria | Actinobacteria | Actinomycetales | Propionibacteriaceae | Propionibacterium acidipropionici |
| Actinobacteria | Actinobacteria | Actinomycetales | Propionibacteriaceae | Propionibacterium acnes |
| Actinobacteria | Actinobacteria | Actinomycetales | Propionibacteriaceae | Propionibacterium avidum |
| Actinobacteria | Actinobacteria | Actinomycetales | Propionibacteriaceae | Propionibacterium freudenreichii |
| Actinobacteria | Actinobacteria | Actinomycetales | Propionibacteriaceae | Propionibacterium propionicum |
| Actinobacteria | Actinobacteria | Actinomycetales | Pseudonocardiaceae | Actinosynnema mirum |
| Actinobacteria | Actinobacteria | Actinomycetales | Pseudonocardiaceae | Amycolatopsis mediterranei |
| Actinobacteria | Actinobacteria | Actinomycetales | Pseudonocardiaceae | Amycolatopsis orientalis |
| Actinobacteria | Actinobacteria | Actinomycetales | Pseudonocardiaceae | Pseudonocardia dioxanivorans |
| Actinobacteria | Actinobacteria | Actinomycetales | Pseudonocardiaceae | Saccharomonospora viridis |
| Actinobacteria | Actinobacteria | Actinomycetales | Pseudonocardiaceae | Saccharopolyspora erythraea |
| Actinobacteria | Actinobacteria | Actinomycetales | Pseudonocardiaceae | Saccharothrix espanaensis |
| Actinobacteria | Actinobacteria | Actinomycetales | Pseudonocardiaceae | Thermobispora bispora |
| Actinobacteria | Actinobacteria | Actinomycetales | Streptomycetaceae | Kitasatospora setae |
| Actinobacteria | Actinobacteria | Actinomycetales | Streptomycetaceae | Streptomyces coelicolor |
| Actinobacteria | Actinobacteria | Actinomycetales | Streptomycetaceae | Streptomyces albus |
| Actinobacteria | Actinobacteria | Actinomycetales | Streptomycetaceae | Streptomyces avermitilis |
| Actinobacteria | Actinobacteria | Actinomycetales | Streptomycetaceae | Streptomyces collinus |
| Actinobacteria | Actinobacteria | Actinomycetales | Streptomycetaceae | Streptomyces fulvissimus |
| Actinobacteria | Actinobacteria | Actinomycetales | Streptomycetaceae | Streptomyces griseus |
| Actinobacteria | Actinobacteria | Actinomycetales | Streptomycetaceae | Streptomyces hygroscopicus |
| Actinobacteria | Actinobacteria | Actinomycetales | Streptomycetaceae | Streptomyces pratensis |
| Actinobacteria | Actinobacteria | Actinomycetales | Streptomycetaceae | Streptomyces rapamycinicus |
| Actinobacteria | Actinobacteria | Actinomycetales | Streptomycetaceae | Streptomyces scabiei |
| Actinobacteria | Actinobacteria | Actinomycetales | Streptomycetaceae | Streptomyces venezuelae |
| Actinobacteria | Actinobacteria | Actinomycetales | Streptomycetaceae | Streptomyces violaceusniger |
| Actinobacteria | Actinobacteria | Actinomycetales | Nocardiopsaceae | Nocardiopsis alba |
| Actinobacteria | Actinobacteria | Actinomycetales | Nocardiopsaceae | Nocardiopsis dassonvillei |
| Actinobacteria | Actinobacteria | Actinomycetales | Nocardiopsaceae | Thermobifida fusca |
| Actinobacteria | Actinobacteria | Actinomycetales | Streptosporangiaceae | Streptosporangium roseum |
| Actinobacteria | Actinobacteria | Actinomycetales | Thermomonosporaceae | Thermomonospora curvata |
| Actinobacteria | Actinobacteria | Bifidobacteriales | Bifidobacteriaceae | Bifidobacterium adolescentis |
| Actinobacteria | Actinobacteria | Bifidobacteriales | Bifidobacteriaceae | Bifidobacterium animalis |
| Actinobacteria | Actinobacteria | Bifidobacteriales | Bifidobacteriaceae | Bifidobacterium asteroides |
| Actinobacteria | Actinobacteria | Bifidobacteriales | Bifidobacteriaceae | Bifidobacterium bifidum |
| Actinobacteria | Actinobacteria | Bifidobacteriales | Bifidobacteriaceae | Bifidobacterium breve |
| Actinobacteria | Actinobacteria | Bifidobacteriales | Bifidobacteriaceae | Bifidobacterium dentium |
| Actinobacteria | Actinobacteria | Bifidobacteriales | Bifidobacteriaceae | Bifidobacterium longum |
| Actinobacteria | Actinobacteria | Bifidobacteriales | Bifidobacteriaceae | Bifidobacterium thermophilum |
| Actinobacteria | Actinobacteria | Bifidobacteriales | Bifidobacteriaceae | Gardnerella vaginalis |
| Actinobacteria | Actinobacteria | Coriobacteriales | Coriobacteriaceae | Adlercreutzia equolifaciens |
| Actinobacteria | Actinobacteria | Coriobacteriales | Coriobacteriaceae | Atopobium parvulum |
| Actinobacteria | Actinobacteria | Coriobacteriales | Coriobacteriaceae | Coriobacterium glomerans |
| Actinobacteria | Actinobacteria | Coriobacteriales | Coriobacteriaceae | Cryptobacterium curtum |
| Actinobacteria | Actinobacteria | Coriobacteriales | Coriobacteriaceae | Eggerthella lenta |
| Actinobacteria | Actinobacteria | Coriobacteriales | Coriobacteriaceae | Gordonibacter pamelaeae |
| Actinobacteria | Actinobacteria | Coriobacteriales | Coriobacteriaceae | Olsenella uli |
| Actinobacteria | Actinobacteria | Coriobacteriales | Coriobacteriaceae | Slackia heliotrinireducens |
| Actinobacteria | Actinobacteria | Rubrobacterales | Rubrobacteraceae | Rubrobacter xylanophilus |
| Actinobacteria | Actinobacteria | Solirubrobacterales | Conexibacteraceae | Conexibacter woesei |
| Aquificae | Aquificae | Aquificales | Aquificaceae | Thermocrinis albus |
| Aquificae | Aquificae | Aquificales | Hydrogenothermaceae | Sulfurihydrogenibium azorense |
| Aquificae | Aquificae | Desulfurobacteriales | Desulfurobacteriaceae | Desulfurobacterium thermolithotrophum |
| Armatimonadetes | Chthonomonadetes | Chthonomonadales | Chthonomonadaceae | Chthonomonas calidirosea |
| Bacteroidetes |  | Bacteroidetes Order II. Incertae sedis | Rhodothermaceae | Rhodothermus marinus |
| Bacteroidetes |  | Bacteroidetes Order II. Incertae sedis | Rhodothermaceae | Salinibacter ruber |
| Bacteroidetes | Bacteroidia | Bacteroidales | Bacteroidaceae | Bacteroides fragilis |
| Bacteroidetes | Bacteroidia | Bacteroidales | Bacteroidaceae | Bacteroides helcogenes |
| Bacteroidetes | Bacteroidia | Bacteroidales | Bacteroidaceae | Bacteroides salanitronis |
| Bacteroidetes | Bacteroidia | Bacteroidales | Bacteroidaceae | Bacteroides thetaiotaomicron |
| Bacteroidetes | Bacteroidia | Bacteroidales | Bacteroidaceae | Bacteroides vulgatus |
| Bacteroidetes | Bacteroidia | Bacteroidales | Bacteroidaceae | Bacteroides xylanisolvens |
| Bacteroidetes | Bacteroidia | Bacteroidales | Porphyromonadaceae | Odoribacter splanchnicus |
| Bacteroidetes | Bacteroidia | Bacteroidales | Porphyromonadaceae | Paludibacter propionicigenes |
| Bacteroidetes | Bacteroidia | Bacteroidales | Porphyromonadaceae | Parabacteroides distasonis |
| Bacteroidetes | Bacteroidia | Bacteroidales | Porphyromonadaceae | Porphyromonas asaccharolytica |
| Bacteroidetes | Bacteroidia | Bacteroidales | Porphyromonadaceae | Porphyromonas gingivalis |
| Bacteroidetes | Bacteroidia | Bacteroidales | Porphyromonadaceae | Tannerella forsythia |
| Bacteroidetes | Bacteroidia | Bacteroidales | Prevotellaceae | Prevotella dentalis |
| Bacteroidetes | Bacteroidia | Bacteroidales | Prevotellaceae | Prevotella denticola |
| Bacteroidetes | Bacteroidia | Bacteroidales | Prevotellaceae | Prevotella intermedia |
| Bacteroidetes | Bacteroidia | Bacteroidales | Prevotellaceae | Prevotella melaninogenica |
| Bacteroidetes | Bacteroidia | Bacteroidales | Prevotellaceae | Prevotella ruminicola |
| Bacteroidetes | Bacteroidia | Bacteroidales | Rikenellaceae | Alistipes finegoldii |
| Bacteroidetes | Bacteroidia | Bacteroidales | Rikenellaceae | Alistipes shahii |
| Bacteroidetes | Cytophagia | Cytophagales | Cyclobacteriaceae | Belliella baltica |
| Bacteroidetes | Cytophagia | Cytophagales | Cyclobacteriaceae | Cyclobacterium marinum |
| Bacteroidetes | Cytophagia | Cytophagales | Cyclobacteriaceae | Echinicola vietnamensis |
| Bacteroidetes | Cytophagia | Cytophagales | Cytophagaceae | Cytophaga hutchinsonii |
| Bacteroidetes | Cytophagia | Cytophagales | Cytophagaceae | Dyadobacter fermentans |
| Bacteroidetes | Cytophagia | Cytophagales | Cytophagaceae | Emticicia oligotrophica |
| Bacteroidetes | Cytophagia | Cytophagales | Cytophagaceae | Fibrella aestuarina |
| Bacteroidetes | Cytophagia | Cytophagales | Cytophagaceae | Flexibacter litoralis |
| Bacteroidetes | Cytophagia | Cytophagales | Cytophagaceae | Leadbetterella byssophila |
| Bacteroidetes | Cytophagia | Cytophagales | Cytophagaceae | Runella slithyformis |
| Bacteroidetes | Cytophagia | Cytophagales | Cytophagaceae | Spirosoma linguale |
| Bacteroidetes | Cytophagia | Cytophagales | Flammeovirgaceae | Marivirga tractuosa |
| Bacteroidetes | Flavobacteriia | Flavobacteriales | Blattabacteriaceae | Blattabacterium punctulatus |
| Bacteroidetes | Flavobacteriia | Flavobacteriales | Cryomorphaceae | Fluviicola taffensis |
| Bacteroidetes | Flavobacteriia | Flavobacteriales | Cryomorphaceae | Owenweeksia hongkongensis |
| Bacteroidetes | Flavobacteriia | Flavobacteriales | Flavobacteriaceae | Aequorivita sublithincola |
| Bacteroidetes | Flavobacteriia | Flavobacteriales | Flavobacteriaceae | Capnocytophaga canimorsus |
| Bacteroidetes | Flavobacteriia | Flavobacteriales | Flavobacteriaceae | Capnocytophaga ochracea |
| Bacteroidetes | Flavobacteriia | Flavobacteriales | Flavobacteriaceae | Cellulophaga algicola |
| Bacteroidetes | Flavobacteriia | Flavobacteriales | Flavobacteriaceae | Cellulophaga lytica |
| Bacteroidetes | Flavobacteriia | Flavobacteriales | Flavobacteriaceae | Croceibacter atlanticus |
| Bacteroidetes | Flavobacteriia | Flavobacteriales | Flavobacteriaceae | Flavobacterium branchiophilum |
| Bacteroidetes | Flavobacteriia | Flavobacteriales | Flavobacteriaceae | Flavobacterium columnare |
| Bacteroidetes | Flavobacteriia | Flavobacteriales | Flavobacteriaceae | Flavobacterium indicum |
| Bacteroidetes | Flavobacteriia | Flavobacteriales | Flavobacteriaceae | Flavobacterium johnsoniae |
| Bacteroidetes | Flavobacteriia | Flavobacteriales | Flavobacteriaceae | Flavobacterium psychrophilum |
| Bacteroidetes | Flavobacteriia | Flavobacteriales | Flavobacteriaceae | Gramella forsetii |
| Bacteroidetes | Flavobacteriia | Flavobacteriales | Flavobacteriaceae | Muricauda ruestringensis |
| Bacteroidetes | Flavobacteriia | Flavobacteriales | Flavobacteriaceae | Nonlabens dokdonensis |
| Bacteroidetes | Flavobacteriia | Flavobacteriales | Flavobacteriaceae | Ornithobacterium rhinotracheale |
| Bacteroidetes | Flavobacteriia | Flavobacteriales | Flavobacteriaceae | Psychroflexus torquis |
| Bacteroidetes | Flavobacteriia | Flavobacteriales | Flavobacteriaceae | Riemerella anatipestifer |
| Bacteroidetes | Flavobacteriia | Flavobacteriales | Flavobacteriaceae | Robiginitalea biformata |
| Bacteroidetes | Flavobacteriia | Flavobacteriales | Flavobacteriaceae | Weeksella virosa |
| Bacteroidetes | Flavobacteriia | Flavobacteriales | Flavobacteriaceae | Zobellia galactanivorans |
| Bacteroidetes | Flavobacteriia | Flavobacteriales | Flavobacteriaceae | Zunongwangia profunda |
| Bacteroidetes | Sphingobacteriia | Sphingobacteriales | Chitinophagaceae | Chitinophaga pinensis |
| Bacteroidetes | Sphingobacteriia | Sphingobacteriales | Chitinophagaceae | Niastella koreensis |
| Bacteroidetes | Sphingobacteriia | Sphingobacteriales | Saprospiraceae | Haliscomenobacter hydrossis |
| Bacteroidetes | Sphingobacteriia | Sphingobacteriales | Saprospiraceae | Saprospira grandis |
| Bacteroidetes | Sphingobacteriia | Sphingobacteriales | Sphingobacteriaceae | Pedobacter heparinus |
| Bacteroidetes | Sphingobacteriia | Sphingobacteriales | Sphingobacteriaceae | Pedobacter saltans |
| Bacteroidetes | Sphingobacteriia | Sphingobacteriales | Sphingobacteriaceae | Solitalea canadensis |
| Chlorobi | Chlorobia | Chlorobiales | Chlorobiaceae | Chlorobaculum parvum |
| Chlorobi | Chlorobia | Chlorobiales | Chlorobiaceae | Chlorobaculum tepidum |
| Chlorobi | Chlorobia | Chlorobiales | Chlorobiaceae | Chlorobium limicola |
| Chlorobi | Chlorobia | Chlorobiales | Chlorobiaceae | Chlorobium phaeobacteroides |
| Chlorobi | Chlorobia | Chlorobiales | Chlorobiaceae | Pelodictyon luteolum |
| Chlorobi | Chlorobia | Chlorobiales | Chlorobiaceae | Pelodictyon phaeoclathratiforme |
| Chlorobi | Chlorobia | Chlorobiales | Chlorobiaceae | Chloroherpeton thalassium |
| Chlorobi | Chlorobia | Chlorobiales | Chlorobiaceae | Prosthecochloris aestuarii |
| Ignavibacteriae | Ignavibacteria | Ignavibacteriales | Ignavibacteriaceae | Ignavibacterium album |
| Chlamydiae | Chlamydiia | Chlamydiales | Chlamydiaceae | Chlamydia pneumoniae |
| Chlamydiae | Chlamydiia | Chlamydiales | Chlamydiaceae | Chlamydia psittaci |
| Chlamydiae | Chlamydiia | Chlamydiales | Chlamydiaceae | Chlamydophila caviae |
| Chlamydiae | Chlamydiia | Chlamydiales | Parachlamydiaceae | Parachlamydia acanthamoebae |
| Verrucomicrobia | Opitutae | Opitutales | Opitutaceae | Opitutus terrae |
| Verrucomicrobia | Opitutae | Puniceicoccales | Puniceicoccaceae | Coraliomargarita akajimensis |
| Verrucomicrobia |  | Methylacidiphilales | Methylacidiphilaceae | Methylacidiphilum infernorum |
| Verrucomicrobia | Verrucomicrobiae | Verrucomicrobiales | Verrucomicrobiaceae | Akkermansia muciniphila |
| Chloroflexi | Anaerolineae | Anaerolineales | Anaerolineaceae | Anaerolinea thermophila |
| Chloroflexi | Caldilineae | Caldilineales | Caldilineaceae | Caldilinea aerophila |
| Chloroflexi | Chloroflexia | Chloroflexales | Chloroflexaceae | Chloroflexus aggregans |
| Chloroflexi | Chloroflexia | Chloroflexales | Roseiflexaceae | Roseiflexus castenholzii |
| Chloroflexi | Chloroflexia | Herpetosiphonales | Herpetosiphonaceae | Herpetosiphon aurantiacus |
| Chloroflexi | Dehalococcoidia | Dehalococcoidales | Dehalococcoidaceae | Dehalococcoides mccartyi |
| Chloroflexi | Thermomicrobia | Sphaerobacterales | Sphaerobacteraceae | Sphaerobacter thermophilus |
| Chloroflexi | Thermomicrobia | Thermomicrobiales | Thermomicrobiaceae | Thermomicrobium roseum |
| Chrysiogenetes | Chrysiogenetes | Chrysiogenales | Chrysiogenaceae | Desulfurispirillum indicum |
| Cyanobacteria | Gloeobacteria | Gloeobacterales |  | Gloeobacter kilaueensis |
| Cyanobacteria | Gloeobacteria | Gloeobacterales |  | Gloeobacter violaceus |
| Cyanobacteria |  | Nostocales | Nostocaceae | Anabaena cylindrica |
| Cyanobacteria |  | Nostocales | Nostocaceae | Anabaena variabilis |
| Cyanobacteria |  | Nostocales | Nostocaceae | Cylindrospermum stagnale |
| Cyanobacteria |  | Nostocales | Nostocaceae | Nostoc punctiforme |
| Cyanobacteria |  | Nostocales | Rivulariaceae | Calothrix parietina |
| Cyanobacteria | Oscillatoriophycideae(subclass) | Chroococcales |  | Acaryochloris marina |
| Cyanobacteria | Oscillatoriophycideae(subclass) | Chroococcales |  | Chamaesiphon minutus |
| Cyanobacteria | Oscillatoriophycideae(subclass) | Chroococcales |  | Cyanobacterium aponinum |
| Cyanobacteria | Oscillatoriophycideae(subclass) | Chroococcales |  | Cyanobacterium stanieri |
| Cyanobacteria | Oscillatoriophycideae(subclass) | Chroococcales |  | Cyanobium gracile |
| Cyanobacteria | Oscillatoriophycideae(subclass) | Chroococcales |  | Dactylococcopsis salina |
| Cyanobacteria | Oscillatoriophycideae(subclass) | Chroococcales |  | Microcystis aeruginosa |
| Cyanobacteria | Oscillatoriophycideae(subclass) | Chroococcales |  | Synechococcus elongatus |
| Cyanobacteria | Oscillatoriophycideae(subclass) | Oscillatoriales |  | Arthrospira platensis |
| Cyanobacteria | Oscillatoriophycideae(subclass) | Oscillatoriales |  | Crinalium epipsammum |
| Cyanobacteria | Oscillatoriophycideae(subclass) | Oscillatoriales |  | Oscillatoria acuminata |
| Cyanobacteria | Oscillatoriophycideae(subclass) | Oscillatoriales |  | Oscillatoria nigro-viridis |
| Cyanobacteria | Oscillatoriophycideae(subclass) | Oscillatoriales |  | Trichodesmium erythraeum |
| Cyanobacteria |  | Pleurocapsales |  | Chroococcidiopsis thermalis |
| Cyanobacteria |  | Pleurocapsales |  | Stanieria cyanosphaera |
| Deferribacteres | Deferribacteres | Deferribacterales | Deferribacteraceae | Calditerrivibrio nitroreducens |
| Deferribacteres | Deferribacteres | Deferribacterales | Deferribacteraceae | Deferribacter desulfuricans |
| Deferribacteres | Deferribacteres | Deferribacterales | Deferribacteraceae | Denitrovibrio acetiphilus |
| Deferribacteres | Deferribacteres | Deferribacterales | Deferribacteraceae | Flexistipes sinusarabici |
| Deinococcus-Thermus | Deinococci | Deinococcales | Deinococcaceae | Deinococcus deserti |
| Deinococcus-Thermus | Deinococci | Deinococcales | Deinococcaceae | Deinococcus geothermalis |
| Deinococcus-Thermus | Deinococci | Deinococcales | Deinococcaceae | Deinococcus gobiensis |
| Deinococcus-Thermus | Deinococci | Deinococcales | Deinococcaceae | Deinococcus maricopensis |
| Deinococcus-Thermus | Deinococci | Deinococcales | Deinococcaceae | Deinococcus peraridilitoris |
| Deinococcus-Thermus | Deinococci | Deinococcales | Deinococcaceae | Deinococcus proteolyticus |
| Deinococcus-Thermus | Deinococci | Deinococcales | Deinococcaceae | Deinococcus radiodurans |
| Deinococcus-Thermus | Deinococci | Deinococcales | Trueperaceae | Truepera radiovictrix |
| Deinococcus-Thermus | Deinococci | Thermales | Thermaceae | Marinithermus hydrothermalis |
| Deinococcus-Thermus | Deinococci | Thermales | Thermaceae | Meiothermus ruber |
| Deinococcus-Thermus | Deinococci | Thermales | Thermaceae | Meiothermus silvanus |
| Deinococcus-Thermus | Deinococci | Thermales | Thermaceae | Oceanithermus profundus |
| Deinococcus-Thermus | Deinococci | Thermales | Thermaceae | Thermus oshimai |
| Deinococcus-Thermus | Deinococci | Thermales | Thermaceae | Thermus scotoductus |
| Deinococcus-Thermus | Deinococci | Thermales | Thermaceae | Thermus thermophilus |
| Dictyoglomi | Dictyoglomia | Dictyoglomales | Dictyoglomaceae | Dictyoglomus thermophilum |
| Elusimicrobia | Elusimicrobia | Elusimicrobiales | Elusimicrobiaceae | Elusimicrobium minutum |
| Acidobacteria | Acidobacteriia | Acidobacteriales | Acidobacteriaceae | Acidobacterium capsulatum |
| Acidobacteria | Acidobacteriia | Acidobacteriales | Acidobacteriaceae | Granulicella mallensis |
| Acidobacteria | Acidobacteriia | Acidobacteriales | Acidobacteriaceae | Granulicella tundricola |
| Acidobacteria | Acidobacteriia | Acidobacteriales | Acidobacteriaceae | Terriglobus roseus |
| Acidobacteria | Acidobacteriia | Acidobacteriales | Acidobacteriaceae | Terriglobus saanensis |
| Fibrobacteres | Fibrobacteria | Fibrobacterales | Fibrobacteraceae | Fibrobacter succinogenes |
| Firmicutes | Bacilli | Bacillales | Alicyclobacillaceae | Alicyclobacillus acidocaldarius |
| Firmicutes | Bacilli | Bacillales | Alicyclobacillaceae | Kyrpidia tusciae |
| Firmicutes | Bacilli | Bacillales | Bacillaceae | Amphibacillus xylanus |
| Firmicutes | Bacilli | Bacillales | Bacillaceae | Anoxybacillus flavithermus |
| Firmicutes | Bacilli | Bacillales | Bacillaceae | Bacillus cellulosilyticus |
| Firmicutes | Bacilli | Bacillales | Bacillaceae | Bacillus anthracis |
| Firmicutes | Bacilli | Bacillales | Bacillaceae | Bacillus cereus |
| Firmicutes | Bacilli | Bacillales | Bacillaceae | Bacillus cytotoxicus |
| Firmicutes | Bacilli | Bacillales | Bacillaceae | Bacillus thuringiensis |
| Firmicutes | Bacilli | Bacillales | Bacillaceae | Bacillus toyonensis |
| Firmicutes | Bacilli | Bacillales | Bacillaceae | Bacillus weihenstephanensis |
| Firmicutes | Bacilli | Bacillales | Bacillaceae | Bacillus clausii |
| Firmicutes | Bacilli | Bacillales | Bacillaceae | Bacillus coagulans |
| Firmicutes | Bacilli | Bacillales | Bacillaceae | Bacillus halodurans |
| Firmicutes | Bacilli | Bacillales | Bacillaceae | Bacillus infantis |
| Firmicutes | Bacilli | Bacillales | Bacillaceae | Bacillus megaterium |
| Firmicutes | Bacilli | Bacillales | Bacillaceae | Bacillus pseudofirmus |
| Firmicutes | Bacilli | Bacillales | Bacillaceae | Bacillus pumilus |
| Firmicutes | Bacilli | Bacillales | Bacillaceae | Bacillus amyloliquefaciens |
| Firmicutes | Bacilli | Bacillales | Bacillaceae | Bacillus atrophaeus |
| Firmicutes | Bacilli | Bacillales | Bacillaceae | Bacillus licheniformis |
| Firmicutes | Bacilli | Bacillales | Bacillaceae | Bacillus subtilis |
| Firmicutes | Bacilli | Bacillales | Bacillaceae | Geobacillus thermodenitrificans |
| Firmicutes | Bacilli | Bacillales | Bacillaceae | Halobacillus halophilus |
| Firmicutes | Bacilli | Bacillales | Bacillaceae | Lysinibacillus sphaericus |
| Firmicutes | Bacilli | Bacillales | Bacillaceae | Oceanobacillus iheyensis |
| Firmicutes | Bacilli | Bacillales |  | Exiguobacterium antarcticum |
| Firmicutes | Bacilli | Bacillales |  | Exiguobacterium sibiricum |
| Firmicutes | Bacilli | Bacillales | Listeriaceae | Listeria innocua |
| Firmicutes | Bacilli | Bacillales | Listeriaceae | Listeria ivanovii |
| Firmicutes | Bacilli | Bacillales | Listeriaceae | Listeria monocytogenes |
| Firmicutes | Bacilli | Bacillales | Listeriaceae | Listeria seeligeri |
| Firmicutes | Bacilli | Bacillales | Listeriaceae | Listeria welshimeri |
| Firmicutes | Bacilli | Bacillales | Paenibacillaceae | Brevibacillus brevis |
| Firmicutes | Bacilli | Bacillales | Paenibacillaceae | Paenibacillus larvae |
| Firmicutes | Bacilli | Bacillales | Paenibacillaceae | Paenibacillus mucilaginosus |
| Firmicutes | Bacilli | Bacillales | Paenibacillaceae | Paenibacillus terrae |
| Firmicutes | Bacilli | Bacillales | Paenibacillaceae | Thermobacillus composti |
| Firmicutes | Bacilli | Bacillales | Planococcaceae | Solibacillus silvestris |
| Firmicutes | Bacilli | Bacillales | Sporolactobacillaceae | Bacillus selenitireducens |
| Firmicutes | Bacilli | Bacillales | Staphylococcaceae | Macrococcus caseolyticus |
| Firmicutes | Bacilli | Bacillales | Staphylococcaceae | Staphylococcus aureus |
| Firmicutes | Bacilli | Bacillales | Staphylococcaceae | Staphylococcus carnosus |
| Firmicutes | Bacilli | Bacillales | Staphylococcaceae | Staphylococcus epidermidis |
| Firmicutes | Bacilli | Bacillales | Staphylococcaceae | Staphylococcus haemolyticus |
| Firmicutes | Bacilli | Bacillales | Staphylococcaceae | Staphylococcus lugdunensis |
| Firmicutes | Bacilli | Bacillales | Staphylococcaceae | Staphylococcus pasteuri |
| Firmicutes | Bacilli | Bacillales | Staphylococcaceae | Staphylococcus pseudintermedius |
| Firmicutes | Bacilli | Bacillales | Staphylococcaceae | Staphylococcus saprophyticus |
| Firmicutes | Bacilli | Bacillales | Staphylococcaceae | Staphylococcus warneri |
| Firmicutes | Bacilli | Lactobacillales | Aerococcaceae | Aerococcus urinae |
| Firmicutes | Bacilli | Lactobacillales | Carnobacteriaceae | Carnobacterium maltaromaticum |
| Firmicutes | Bacilli | Lactobacillales | Enterococcaceae | Enterococcus casseliflavus |
| Firmicutes | Bacilli | Lactobacillales | Enterococcaceae | Enterococcus faecalis |
| Firmicutes | Bacilli | Lactobacillales | Enterococcaceae | Enterococcus faecium |
| Firmicutes | Bacilli | Lactobacillales | Enterococcaceae | Enterococcus hirae |
| Firmicutes | Bacilli | Lactobacillales | Enterococcaceae | Enterococcus mundtii |
| Firmicutes | Bacilli | Lactobacillales | Enterococcaceae | Melissococcus plutonius |
| Firmicutes | Bacilli | Lactobacillales | Enterococcaceae | Tetragenococcus halophilus |
| Firmicutes | Bacilli | Lactobacillales | Lactobacillaceae | Lactobacillus acidophilus |
| Firmicutes | Bacilli | Lactobacillales | Lactobacillaceae | Lactobacillus amylovorus |
| Firmicutes | Bacilli | Lactobacillales | Lactobacillaceae | Lactobacillus brevis |
| Firmicutes | Bacilli | Lactobacillales | Lactobacillaceae | Lactobacillus buchneri |
| Firmicutes | Bacilli | Lactobacillales | Lactobacillaceae | Lactobacillus casei |
| Firmicutes | Bacilli | Lactobacillales | Lactobacillaceae | Lactobacillus crispatus |
| Firmicutes | Bacilli | Lactobacillales | Lactobacillaceae | Lactobacillus delbrueckii |
| Firmicutes | Bacilli | Lactobacillales | Lactobacillaceae | Lactobacillus fermentum |
| Firmicutes | Bacilli | Lactobacillales | Lactobacillaceae | Lactobacillus gasseri |
| Firmicutes | Bacilli | Lactobacillales | Lactobacillaceae | Lactobacillus helveticus |
| Firmicutes | Bacilli | Lactobacillales | Lactobacillaceae | Lactobacillus johnsonii |
| Firmicutes | Bacilli | Lactobacillales | Lactobacillaceae | Lactobacillus kefiranofaciens |
| Firmicutes | Bacilli | Lactobacillales | Lactobacillaceae | Lactobacillus plantarum |
| Firmicutes | Bacilli | Lactobacillales | Lactobacillaceae | Lactobacillus reuteri |
| Firmicutes | Bacilli | Lactobacillales | Lactobacillaceae | Lactobacillus rhamnosus |
| Firmicutes | Bacilli | Lactobacillales | Lactobacillaceae | Lactobacillus ruminis |
| Firmicutes | Bacilli | Lactobacillales | Lactobacillaceae | Lactobacillus sakei |
| Firmicutes | Bacilli | Lactobacillales | Lactobacillaceae | Lactobacillus salivarius |
| Firmicutes | Bacilli | Lactobacillales | Lactobacillaceae | Lactobacillus sanfranciscensis |
| Firmicutes | Bacilli | Lactobacillales | Lactobacillaceae | Pediococcus claussenii |
| Firmicutes | Bacilli | Lactobacillales | Lactobacillaceae | Pediococcus pentosaceus |
| Firmicutes | Bacilli | Lactobacillales | Leuconostocaceae | Leuconostoc carnosum |
| Firmicutes | Bacilli | Lactobacillales | Leuconostocaceae | Leuconostoc citreum |
| Firmicutes | Bacilli | Lactobacillales | Leuconostocaceae | Leuconostoc gelidum |
| Firmicutes | Bacilli | Lactobacillales | Leuconostocaceae | Leuconostoc kimchii |
| Firmicutes | Bacilli | Lactobacillales | Leuconostocaceae | Leuconostoc mesenteroides |
| Firmicutes | Bacilli | Lactobacillales | Leuconostocaceae | Oenococcus oeni |
| Firmicutes | Bacilli | Lactobacillales | Leuconostocaceae | Weissella koreensis |
| Firmicutes | Bacilli | Lactobacillales | Streptococcaceae | Lactococcus garvieae |
| Firmicutes | Bacilli | Lactobacillales | Streptococcaceae | Lactococcus lactis |
| Firmicutes | Bacilli | Lactobacillales | Streptococcaceae | Streptococcus agalactiae |
| Firmicutes | Bacilli | Lactobacillales | Streptococcaceae | Streptococcus anginosus |
| Firmicutes | Bacilli | Lactobacillales | Streptococcaceae | Streptococcus constellatus |
| Firmicutes | Bacilli | Lactobacillales | Streptococcaceae | Streptococcus intermedius |
| Firmicutes | Bacilli | Lactobacillales | Streptococcaceae | Streptococcus dysgalactiae |
| Firmicutes | Bacilli | Lactobacillales | Streptococcaceae | Streptococcus equi |
| Firmicutes | Bacilli | Lactobacillales | Streptococcaceae | Streptococcus gallolyticus |
| Firmicutes | Bacilli | Lactobacillales | Streptococcaceae | Streptococcus gordonii |
| Firmicutes | Bacilli | Lactobacillales | Streptococcaceae | Streptococcus infantarius |
| Firmicutes | Bacilli | Lactobacillales | Streptococcaceae | Streptococcus iniae |
| Firmicutes | Bacilli | Lactobacillales | Streptococcaceae | Streptococcus lutetiensis |
| Firmicutes | Bacilli | Lactobacillales | Streptococcaceae | Streptococcus mitis |
| Firmicutes | Bacilli | Lactobacillales | Streptococcaceae | Streptococcus mutans |
| Firmicutes | Bacilli | Lactobacillales | Streptococcaceae | Streptococcus oligofermentans |
| Firmicutes | Bacilli | Lactobacillales | Streptococcaceae | Streptococcus oralis |
| Firmicutes | Bacilli | Lactobacillales | Streptococcaceae | Streptococcus parasanguinis |
| Firmicutes | Bacilli | Lactobacillales | Streptococcaceae | Streptococcus parauberis |
| Firmicutes | Bacilli | Lactobacillales | Streptococcaceae | Streptococcus pasteurianus |
| Firmicutes | Bacilli | Lactobacillales | Streptococcaceae | Streptococcus pneumoniae |
| Firmicutes | Bacilli | Lactobacillales | Streptococcaceae | Streptococcus pseudopneumoniae |
| Firmicutes | Bacilli | Lactobacillales | Streptococcaceae | Streptococcus pyogenes |
| Firmicutes | Bacilli | Lactobacillales | Streptococcaceae | Streptococcus salivarius |
| Firmicutes | Bacilli | Lactobacillales | Streptococcaceae | Streptococcus sanguinis |
| Firmicutes | Bacilli | Lactobacillales | Streptococcaceae | Streptococcus suis |
| Firmicutes | Bacilli | Lactobacillales | Streptococcaceae | Streptococcus thermophilus |
| Firmicutes | Bacilli | Lactobacillales | Streptococcaceae | Streptococcus uberis |
| Firmicutes | Clostridia | Clostridiales | Clostridiaceae | Alkaliphilus metalliredigens |
| Firmicutes | Clostridia | Clostridiales | Clostridiaceae | Alkaliphilus oremlandii |
| Firmicutes | Clostridia | Clostridiales | Clostridiaceae | Clostridium acetobutylicum |
| Firmicutes | Clostridia | Clostridiales | Clostridiaceae | Clostridium beijerinckii |
| Firmicutes | Clostridia | Clostridiales | Clostridiaceae | Clostridium botulinum |
| Firmicutes | Clostridia | Clostridiales | Clostridiaceae | Clostridium cellulolyticum |
| Firmicutes | Clostridia | Clostridiales | Clostridiaceae | Clostridium cellulovorans |
| Firmicutes | Clostridia | Clostridiales | Clostridiaceae | Clostridium kluyveri |
| Firmicutes | Clostridia | Clostridiales | Clostridiaceae | Clostridium ljungdahlii |
| Firmicutes | Clostridia | Clostridiales | Clostridiaceae | Clostridium novyi |
| Firmicutes | Clostridia | Clostridiales | Clostridiaceae | Clostridium pasteurianum |
| Firmicutes | Clostridia | Clostridiales | Clostridiaceae | Clostridium perfringens |
| Firmicutes | Clostridia | Clostridiales | Clostridiaceae | Clostridium saccharobutylicum |
| Firmicutes | Clostridia | Clostridiales | Clostridiaceae | Clostridium saccharoperbutylacetonicum |
| Firmicutes | Clostridia | Clostridiales | Clostridiaceae | Clostridium tetani |
| Firmicutes | Clostridia | Clostridiales | Clostridiales Family XVII. Incertae Sedis | Sulfobacillus acidophilus |
| Firmicutes | Clostridia | Clostridiales | Clostridiales Family XVII. Incertae Sedis | Thermaerobacter marianensis |
| Firmicutes | Clostridia | Clostridiales | Eubacteriaceae | Acetobacterium woodii |
| Firmicutes | Clostridia | Clostridiales | Eubacteriaceae | Eubacterium eligens |
| Firmicutes | Clostridia | Clostridiales | Eubacteriaceae | Eubacterium limosum |
| Firmicutes | Clostridia | Clostridiales | Eubacteriaceae | Eubacterium rectale |
| Firmicutes | Clostridia | Clostridiales | Heliobacteriaceae | Heliobacterium modesticaldum |
| Firmicutes | Clostridia | Clostridiales | Lachnospiraceae | Ruminococcus obeum |
| Firmicutes | Clostridia | Clostridiales | Lachnospiraceae | Ruminococcus torques |
| Firmicutes | Clostridia | Clostridiales | Lachnospiraceae | Butyrivibrio fibrisolvens |
| Firmicutes | Clostridia | Clostridiales | Lachnospiraceae | Butyrivibrio proteoclasticus |
| Firmicutes | Clostridia | Clostridiales | Lachnospiraceae | Cellulosilyticum lentocellum |
| Firmicutes | Clostridia | Clostridiales | Lachnospiraceae | Coprococcus catus |
| Firmicutes | Clostridia | Clostridiales | Lachnospiraceae | Clostridium saccharolyticum |
| Firmicutes | Clostridia | Clostridiales | Lachnospiraceae | Lachnoclostridium phytofermentans |
| Firmicutes | Clostridia | Clostridiales | Lachnospiraceae | Roseburia hominis |
| Firmicutes | Clostridia | Clostridiales | Lachnospiraceae | Roseburia intestinalis |
| Firmicutes | Clostridia | Clostridiales | Oscillospiraceae | Oscillibacter valericigenes |
| Firmicutes | Clostridia | Clostridiales | Peptococcaceae | Desulfitobacterium dehalogenans |
| Firmicutes | Clostridia | Clostridiales | Peptococcaceae | Desulfitobacterium dichloroeliminans |
| Firmicutes | Clostridia | Clostridiales | Peptococcaceae | Desulfitobacterium hafniense |
| Firmicutes | Clostridia | Clostridiales | Peptococcaceae | Desulfosporosinus acidiphilus |
| Firmicutes | Clostridia | Clostridiales | Peptococcaceae | Desulfosporosinus meridiei |
| Firmicutes | Clostridia | Clostridiales | Peptococcaceae | Desulfosporosinus orientis |
| Firmicutes | Clostridia | Clostridiales | Peptococcaceae | Desulfotomaculum acetoxidans |
| Firmicutes | Clostridia | Clostridiales | Peptococcaceae | Desulfotomaculum carboxydivorans |
| Firmicutes | Clostridia | Clostridiales | Peptococcaceae | Desulfotomaculum gibsoniae |
| Firmicutes | Clostridia | Clostridiales | Peptococcaceae | Desulfotomaculum kuznetsovii |
| Firmicutes | Clostridia | Clostridiales | Peptococcaceae | Desulfotomaculum reducens |
| Firmicutes | Clostridia | Clostridiales | Peptococcaceae | Desulfotomaculum ruminis |
| Firmicutes | Clostridia | Clostridiales | Peptococcaceae | Pelotomaculum thermopropionicum |
| Firmicutes | Clostridia | Clostridiales | Peptococcaceae | Syntrophobotulus glycolicus |
| Firmicutes | Clostridia | Clostridiales | Peptococcaceae | Thermincola potens |
| Firmicutes | Clostridia | Clostridiales | Peptoniphilaceae | Anaerococcus prevotii |
| Firmicutes | Clostridia | Clostridiales | Peptoniphilaceae | Finegoldia magna |
| Firmicutes | Clostridia | Clostridiales | Peptostreptococcaceae | Filifactor alocis |
| Firmicutes | Clostridia | Clostridiales | Peptostreptococcaceae | Clostridium sticklandii |
| Firmicutes | Clostridia | Clostridiales | Peptostreptococcaceae | Peptoclostridium difficile |
| Firmicutes | Clostridia | Clostridiales | Ruminococcaceae | Ethanoligenens harbinense |
| Firmicutes | Clostridia | Clostridiales | Ruminococcaceae | Faecalibacterium prausnitzii |
| Firmicutes | Clostridia | Clostridiales | Ruminococcaceae | Clostridium clariflavum |
| Firmicutes | Clostridia | Clostridiales | Ruminococcaceae | Clostridium stercorarium |
| Firmicutes | Clostridia | Clostridiales | Ruminococcaceae | Eubacterium siraeum |
| Firmicutes | Clostridia | Clostridiales | Ruminococcaceae | Ruminococcus albus |
| Firmicutes | Clostridia | Clostridiales | Ruminococcaceae | Ruminococcus bromii |
| Firmicutes | Clostridia | Clostridiales | Ruminococcaceae | Ruminococcus champanellensis |
| Firmicutes | Clostridia | Clostridiales | Symbiobacteriaceae | Symbiobacterium thermophilum |
| Firmicutes | Clostridia | Clostridiales | Syntrophomonadaceae | Syntrophomonas wolfei |
| Firmicutes | Clostridia | Clostridiales |  | Gottschalkia acidurici |
| Firmicutes | Clostridia | Halanaerobiales | Halanaerobiaceae | Halanaerobium hydrogeniformans |
| Firmicutes | Clostridia | Halanaerobiales | Halanaerobiaceae | Halanaerobium praevalens |
| Firmicutes | Clostridia | Halanaerobiales | Halanaerobiaceae | Halothermothrix orenii |
| Firmicutes | Clostridia | Halanaerobiales | Halobacteroidaceae | Acetohalobium arabaticum |
| Firmicutes | Clostridia | Halanaerobiales | Halobacteroidaceae | Halobacteroides halobius |
| Firmicutes | Clostridia | Natranaerobiales | Natranaerobiaceae | Natranaerobius thermophilus |
| Firmicutes | Clostridia | Thermoanaerobacterales | Thermoanaerobacteraceae | Caldanaerobacter subterraneus |
| Firmicutes | Clostridia | Thermoanaerobacterales | Thermoanaerobacteraceae | Ammonifex degensii |
| Firmicutes | Clostridia | Thermoanaerobacterales | Thermoanaerobacteraceae | Moorella thermoacetica |
| Firmicutes | Clostridia | Thermoanaerobacterales | Thermoanaerobacteraceae | Tepidanaerobacter acetatoxydans |
| Firmicutes | Clostridia | Thermoanaerobacterales | Thermoanaerobacteraceae | Thermacetogenium phaeum |
| Firmicutes | Clostridia | Thermoanaerobacterales | Thermoanaerobacteraceae | Thermoanaerobacter italicus |
| Firmicutes | Clostridia | Thermoanaerobacterales | Thermoanaerobacteraceae | Thermoanaerobacter wiegelii |
| Firmicutes | Clostridia | Thermoanaerobacterales | Thermoanaerobacterales Family III. Incertae Sedis | Caldicellulosiruptor kristjanssonii |
| Firmicutes | Clostridia | Thermoanaerobacterales | Thermoanaerobacterales Family III. Incertae Sedis | Caldicellulosiruptor owensensis |
| Firmicutes | Clostridia | Thermoanaerobacterales | Thermoanaerobacterales Family III. Incertae Sedis | Caldicellulosiruptor saccharolyticus |
| Firmicutes | Clostridia | Thermoanaerobacterales | Thermoanaerobacterales Family III. Incertae Sedis | Thermoanaerobacterium saccharolyticum |
| Firmicutes | Clostridia | Thermoanaerobacterales | Thermoanaerobacterales Family III. Incertae Sedis | Thermoanaerobacterium thermosaccharolyticum |
| Firmicutes | Clostridia | Thermoanaerobacterales | Thermoanaerobacterales Family III. Incertae Sedis | Thermoanaerobacterium xylanolyticum |
| Firmicutes | Clostridia | Thermoanaerobacterales | Thermoanaerobacterales Family III. Incertae Sedis | Thermosediminibacter oceani |
| Firmicutes | Clostridia | Thermoanaerobacterales | Thermoanaerobacterales Family IV. Incertae Sedis | Mahella australiensis |
| Firmicutes | Clostridia | Thermoanaerobacterales | Thermodesulfobiaceae | Coprothermobacter proteolyticus |
| Firmicutes | Clostridia | Thermoanaerobacterales | Thermodesulfobiaceae | Thermodesulfobium narugense |
| Firmicutes | Erysipelotrichia | Erysipelotrichales | Erysipelotrichaceae | Erysipelothrix rhusiopathiae |
| Firmicutes | Negativicutes | Selenomonadales | Acidaminococcaceae | Acidaminococcus fermentans |
| Firmicutes | Negativicutes | Selenomonadales | Acidaminococcaceae | Acidaminococcus intestini |
| Firmicutes | Negativicutes | Selenomonadales | Veillonellaceae | Megamonas hypermegale |
| Firmicutes | Negativicutes | Selenomonadales | Veillonellaceae | Megasphaera elsdenii |
| Firmicutes | Negativicutes | Selenomonadales | Veillonellaceae | Selenomonas ruminantium |
| Firmicutes | Negativicutes | Selenomonadales | Veillonellaceae | Selenomonas sputigena |
| Firmicutes | Negativicutes | Selenomonadales | Veillonellaceae | Veillonella parvula |
| Fusobacteria | Fusobacteriia | Fusobacteriales | Fusobacteriaceae | Fusobacterium nucleatum |
| Fusobacteria | Fusobacteriia | Fusobacteriales | Fusobacteriaceae | Ilyobacter polytropus |
| Fusobacteria | Fusobacteriia | Fusobacteriales | Leptotrichiaceae | Leptotrichia buccalis |
| Fusobacteria | Fusobacteriia | Fusobacteriales | Leptotrichiaceae | Sebaldella termitidis |
| Fusobacteria | Fusobacteriia | Fusobacteriales | Leptotrichiaceae | Streptobacillus moniliformis |
| Gemmatimonadetes | Gemmatimonadetes | Gemmatimonadales | Gemmatimonadaceae | Gemmatimonas aurantiaca |
| Nitrospirae | Nitrospira | Nitrospirales | Nitrospiraceae | Leptospirillum ferrooxidans |
| Planctomycetes | Phycisphaerae | Phycisphaerales | Phycisphaeraceae | Phycisphaera mikurensis |
| Planctomycetes | Planctomycetia | Planctomycetales | Planctomycetaceae | Isosphaera pallida |
| Planctomycetes | Planctomycetia | Planctomycetales | Planctomycetaceae | Pirellula staleyi |
| Planctomycetes | Planctomycetia | Planctomycetales | Planctomycetaceae | Planctomyces brasiliensis |
| Planctomycetes | Planctomycetia | Planctomycetales | Planctomycetaceae | Planctomyces limnophilus |
| Planctomycetes | Planctomycetia | Planctomycetales | Planctomycetaceae | Rhodopirellula baltica |
| Planctomycetes | Planctomycetia | Planctomycetales | Planctomycetaceae | Singulisphaera acidiphila |
| Proteobacteria | Alphaproteobacteria | Caulobacterales | Caulobacteraceae | Asticcacaulis excentricus |
| Proteobacteria | Alphaproteobacteria | Caulobacterales | Caulobacteraceae | Brevundimonas subvibrioides |
| Proteobacteria | Alphaproteobacteria | Caulobacterales | Caulobacteraceae | Caulobacter segnis |
| Proteobacteria | Alphaproteobacteria | Caulobacterales | Caulobacteraceae | Caulobacter vibrioides |
| Proteobacteria | Alphaproteobacteria | Caulobacterales | Caulobacteraceae | Phenylobacterium zucineum |
| Proteobacteria | Alphaproteobacteria | Magnetococcales | Magnetococcaceae | Magnetococcus marinus |
| Proteobacteria | Alphaproteobacteria | Parvularculales | Parvularculaceae | Parvularcula bermudensis |
| Proteobacteria | Alphaproteobacteria | Rhizobiales | Bartonellaceae | Bartonella australis |
| Proteobacteria | Alphaproteobacteria | Rhizobiales | Bartonellaceae | Bartonella clarridgeiae |
| Proteobacteria | Alphaproteobacteria | Rhizobiales | Bartonellaceae | Bartonella grahamii |
| Proteobacteria | Alphaproteobacteria | Rhizobiales | Bartonellaceae | Bartonella henselae |
| Proteobacteria | Alphaproteobacteria | Rhizobiales | Bartonellaceae | Bartonella quintana |
| Proteobacteria | Alphaproteobacteria | Rhizobiales | Bartonellaceae | Bartonella tribocorum |
| Proteobacteria | Alphaproteobacteria | Rhizobiales | Bartonellaceae | Bartonella vinsonii |
| Proteobacteria | Alphaproteobacteria | Rhizobiales | Beijerinckiaceae | Beijerinckia indica |
| Proteobacteria | Alphaproteobacteria | Rhizobiales | Beijerinckiaceae | Methylocella silvestris |
| Proteobacteria | Alphaproteobacteria | Rhizobiales | Bradyrhizobiaceae | Bradyrhizobium diazoefficiens |
| Proteobacteria | Alphaproteobacteria | Rhizobiales | Bradyrhizobiaceae | Bradyrhizobium japonicum |
| Proteobacteria | Alphaproteobacteria | Rhizobiales | Bradyrhizobiaceae | Bradyrhizobium oligotrophicum |
| Proteobacteria | Alphaproteobacteria | Rhizobiales | Bradyrhizobiaceae | Nitrobacter hamburgensis |
| Proteobacteria | Alphaproteobacteria | Rhizobiales | Bradyrhizobiaceae | Nitrobacter winogradskyi |
| Proteobacteria | Alphaproteobacteria | Rhizobiales | Bradyrhizobiaceae | Oligotropha carboxidovorans |
| Proteobacteria | Alphaproteobacteria | Rhizobiales | Bradyrhizobiaceae | Rhodopseudomonas palustris |
| Proteobacteria | Alphaproteobacteria | Rhizobiales | Brucellaceae | Ochrobactrum anthropi |
| Proteobacteria | Alphaproteobacteria | Rhizobiales | Hyphomicrobiaceae | Hyphomicrobium denitrificans |
| Proteobacteria | Alphaproteobacteria | Rhizobiales | Hyphomicrobiaceae | Hyphomicrobium nitrativorans |
| Proteobacteria | Alphaproteobacteria | Rhizobiales | Hyphomicrobiaceae | Pelagibacterium halotolerans |
| Proteobacteria | Alphaproteobacteria | Rhizobiales | Hyphomicrobiaceae | Rhodomicrobium vannielii |
| Proteobacteria | Alphaproteobacteria | Rhizobiales | Methylobacteriaceae | Methylobacterium extorquens |
| Proteobacteria | Alphaproteobacteria | Rhizobiales | Methylobacteriaceae | Methylobacterium nodulans |
| Proteobacteria | Alphaproteobacteria | Rhizobiales | Methylobacteriaceae | Methylobacterium populi |
| Proteobacteria | Alphaproteobacteria | Rhizobiales | Methylobacteriaceae | Methylobacterium radiotolerans |
| Proteobacteria | Alphaproteobacteria | Rhizobiales | Phyllobacteriaceae | Mesorhizobium australicum |
| Proteobacteria | Alphaproteobacteria | Rhizobiales | Phyllobacteriaceae | Mesorhizobium ciceri |
| Proteobacteria | Alphaproteobacteria | Rhizobiales | Phyllobacteriaceae | Mesorhizobium loti |
| Proteobacteria | Alphaproteobacteria | Rhizobiales | Phyllobacteriaceae | Mesorhizobium opportunistum |
| Proteobacteria | Alphaproteobacteria | Rhizobiales | Rhizobiaceae | Liberibacter crescens |
| Proteobacteria | Alphaproteobacteria | Rhizobiales | Rhizobiaceae | Agrobacterium fabrum |
| Proteobacteria | Alphaproteobacteria | Rhizobiales | Rhizobiaceae | Agrobacterium tumefaciens |
| Proteobacteria | Alphaproteobacteria | Rhizobiales | Rhizobiaceae | Agrobacterium vitis |
| Proteobacteria | Alphaproteobacteria | Rhizobiales | Rhizobiaceae | Rhizobium etli |
| Proteobacteria | Alphaproteobacteria | Rhizobiales | Rhizobiaceae | Rhizobium leguminosarum |
| Proteobacteria | Alphaproteobacteria | Rhizobiales | Rhizobiaceae | Rhizobium tropici |
| Proteobacteria | Alphaproteobacteria | Rhizobiales | Rhizobiaceae | Sinorhizobium fredii |
| Proteobacteria | Alphaproteobacteria | Rhizobiales | Rhizobiaceae | Sinorhizobium medicae |
| Proteobacteria | Alphaproteobacteria | Rhizobiales | Rhizobiaceae | Sinorhizobium meliloti |
| Proteobacteria | Alphaproteobacteria | Rhizobiales | Rhodobiaceae | Parvibaculum lavamentivorans |
| Proteobacteria | Alphaproteobacteria | Rhizobiales | Xanthobacteraceae | Azorhizobium caulinodans |
| Proteobacteria | Alphaproteobacteria | Rhizobiales | Xanthobacteraceae | Starkeya novella |
| Proteobacteria | Alphaproteobacteria | Rhizobiales | Xanthobacteraceae | Xanthobacter autotrophicus |
| Proteobacteria | Alphaproteobacteria | Rhodobacterales | Hyphomonadaceae | Hirschia baltica |
| Proteobacteria | Alphaproteobacteria | Rhodobacterales | Hyphomonadaceae | Hyphomonas neptunium |
| Proteobacteria | Alphaproteobacteria | Rhodobacterales | Hyphomonadaceae | Maricaulis maris |
| Proteobacteria | Alphaproteobacteria | Rhodobacterales | Rhodobacteraceae | Dinoroseobacter shibae |
| Proteobacteria | Alphaproteobacteria | Rhodobacterales | Rhodobacteraceae | Ketogulonicigenium vulgare |
| Proteobacteria | Alphaproteobacteria | Rhodobacterales | Rhodobacteraceae | Leisingera methylohalidivorans |
| Proteobacteria | Alphaproteobacteria | Rhodobacterales | Rhodobacteraceae | Octadecabacter antarcticus |
| Proteobacteria | Alphaproteobacteria | Rhodobacterales | Rhodobacteraceae | Octadecabacter arcticus |
| Proteobacteria | Alphaproteobacteria | Rhodobacterales | Rhodobacteraceae | Paracoccus aminophilus |
| Proteobacteria | Alphaproteobacteria | Rhodobacterales | Rhodobacteraceae | Paracoccus denitrificans |
| Proteobacteria | Alphaproteobacteria | Rhodobacterales | Rhodobacteraceae | Phaeobacter gallaeciensis |
| Proteobacteria | Alphaproteobacteria | Rhodobacterales | Rhodobacteraceae | Phaeobacter inhibens |
| Proteobacteria | Alphaproteobacteria | Rhodobacterales | Rhodobacteraceae | Rhodobacter capsulatus |
| Proteobacteria | Alphaproteobacteria | Rhodobacterales | Rhodobacteraceae | Rhodobacter sphaeroides |
| Proteobacteria | Alphaproteobacteria | Rhodobacterales | Rhodobacteraceae | Roseobacter denitrificans |
| Proteobacteria | Alphaproteobacteria | Rhodobacterales | Rhodobacteraceae | Roseobacter litoralis |
| Proteobacteria | Alphaproteobacteria | Rhodobacterales | Rhodobacteraceae | Ruegeria pomeroyi |
| Proteobacteria | Alphaproteobacteria | Rhodospirillales | Acetobacteraceae | Acetobacter pasteurianus |
| Proteobacteria | Alphaproteobacteria | Rhodospirillales | Acetobacteraceae | Acidiphilium cryptum |
| Proteobacteria | Alphaproteobacteria | Rhodospirillales | Acetobacteraceae | Acidiphilium multivorum |
| Proteobacteria | Alphaproteobacteria | Rhodospirillales | Acetobacteraceae | Gluconacetobacter diazotrophicus |
| Proteobacteria | Alphaproteobacteria | Rhodospirillales | Acetobacteraceae | Gluconobacter oxydans |
| Proteobacteria | Alphaproteobacteria | Rhodospirillales | Acetobacteraceae | Granulibacter bethesdensis |
| Proteobacteria | Alphaproteobacteria | Rhodospirillales | Acetobacteraceae | Komagataeibacter medellinensis |
| Proteobacteria | Alphaproteobacteria | Rhodospirillales | Rhodospirillaceae | Azospirillum brasilense |
| Proteobacteria | Alphaproteobacteria | Rhodospirillales | Rhodospirillaceae | Azospirillum lipoferum |
| Proteobacteria | Alphaproteobacteria | Rhodospirillales | Rhodospirillaceae | Magnetospirillum gryphiswaldense |
| Proteobacteria | Alphaproteobacteria | Rhodospirillales | Rhodospirillaceae | Magnetospirillum magneticum |
| Proteobacteria | Alphaproteobacteria | Rhodospirillales | Rhodospirillaceae | Rhodospirillum photometricum |
| Proteobacteria | Alphaproteobacteria | Rhodospirillales | Rhodospirillaceae | Rhodospirillum rubrum |
| Proteobacteria | Alphaproteobacteria | Rhodospirillales | Rhodospirillaceae | Tistrella mobilis |
| Proteobacteria | Alphaproteobacteria | Rickettsiales | Anaplasmataceae | Anaplasma phagocytophilum |
| Proteobacteria | Alphaproteobacteria | Rickettsiales | Anaplasmataceae | Ehrlichia chaffeensis |
| Proteobacteria | Alphaproteobacteria | Rickettsiales | Anaplasmataceae | Neorickettsia risticii |
| Proteobacteria | Alphaproteobacteria | Rickettsiales | Rickettsiaceae | Orientia tsutsugamushi |
| Proteobacteria | Alphaproteobacteria | Rickettsiales | Rickettsiaceae | Rickettsia canadensis |
| Proteobacteria | Alphaproteobacteria | Rickettsiales | Rickettsiaceae | Rickettsia akari |
| Proteobacteria | Alphaproteobacteria | Rickettsiales | Rickettsiaceae | Rickettsia felis |
| Proteobacteria | Alphaproteobacteria | Rickettsiales | Rickettsiaceae | Rickettsia rickettsii |
| Proteobacteria | Alphaproteobacteria | Rickettsiales | Rickettsiaceae | Rickettsia prowazekii |
| Proteobacteria | Alphaproteobacteria | Rickettsiales | Rickettsiaceae | Rickettsia typhi |
| Proteobacteria | Alphaproteobacteria | Sphingomonadales | Erythrobacteraceae | Erythrobacter litoralis |
| Proteobacteria | Alphaproteobacteria | Sphingomonadales | Sphingomonadaceae | Novosphingobium aromaticivorans |
| Proteobacteria | Alphaproteobacteria | Sphingomonadales | Sphingomonadaceae | Sphingobium japonicum |
| Proteobacteria | Alphaproteobacteria | Sphingomonadales | Sphingomonadaceae | Sphingomonas wittichii |
| Proteobacteria | Alphaproteobacteria | Sphingomonadales | Sphingomonadaceae | Sphingopyxis alaskensis |
| Proteobacteria | Alphaproteobacteria | Sphingomonadales | Sphingomonadaceae | Zymomonas mobilis |
| Proteobacteria | Alphaproteobacteria |  | Micavibrio aeruginosavorus |  |
| Proteobacteria | Alphaproteobacteria |  | Polymorphum gilvum |  |
| Proteobacteria | Betaproteobacteria | Burkholderiales | Alcaligenaceae | Achromobacter xylosoxidans |
| Proteobacteria | Betaproteobacteria | Burkholderiales | Alcaligenaceae | Advenella kashmirensis |
| Proteobacteria | Betaproteobacteria | Burkholderiales | Alcaligenaceae | Bordetella avium |
| Proteobacteria | Betaproteobacteria | Burkholderiales | Alcaligenaceae | Bordetella bronchiseptica |
| Proteobacteria | Betaproteobacteria | Burkholderiales | Alcaligenaceae | Bordetella parapertussis |
| Proteobacteria | Betaproteobacteria | Burkholderiales | Alcaligenaceae | Bordetella petrii |
| Proteobacteria | Betaproteobacteria | Burkholderiales | Alcaligenaceae | Taylorella asinigenitalis |
| Proteobacteria | Betaproteobacteria | Burkholderiales | Alcaligenaceae | Taylorella equigenitalis |
| Proteobacteria | Betaproteobacteria | Burkholderiales | Burkholderiaceae | Burkholderia ambifaria |
| Proteobacteria | Betaproteobacteria | Burkholderiales | Burkholderiaceae | Burkholderia cenocepacia |
| Proteobacteria | Betaproteobacteria | Burkholderiales | Burkholderiaceae | Burkholderia cepacia |
| Proteobacteria | Betaproteobacteria | Burkholderiales | Burkholderiaceae | Burkholderia lata |
| Proteobacteria | Betaproteobacteria | Burkholderiales | Burkholderiaceae | Burkholderia multivorans |
| Proteobacteria | Betaproteobacteria | Burkholderiales | Burkholderiaceae | Burkholderia vietnamiensis |
| Proteobacteria | Betaproteobacteria | Burkholderiales | Burkholderiaceae | Burkholderia gladioli |
| Proteobacteria | Betaproteobacteria | Burkholderiales | Burkholderiaceae | Burkholderia glumae |
| Proteobacteria | Betaproteobacteria | Burkholderiales | Burkholderiaceae | Burkholderia phenoliruptrix |
| Proteobacteria | Betaproteobacteria | Burkholderiales | Burkholderiaceae | Burkholderia phymatum |
| Proteobacteria | Betaproteobacteria | Burkholderiales | Burkholderiaceae | Burkholderia phytofirmans |
| Proteobacteria | Betaproteobacteria | Burkholderiales | Burkholderiaceae | Burkholderia rhizoxinica |
| Proteobacteria | Betaproteobacteria | Burkholderiales | Burkholderiaceae | Burkholderia xenovorans |
| Proteobacteria | Betaproteobacteria | Burkholderiales | Burkholderiaceae | Burkholderia pseudomallei |
| Proteobacteria | Betaproteobacteria | Burkholderiales | Burkholderiaceae | Burkholderia thailandensis |
| Proteobacteria | Betaproteobacteria | Burkholderiales | Burkholderiaceae | Cupriavidus metallidurans |
| Proteobacteria | Betaproteobacteria | Burkholderiales | Burkholderiaceae | Cupriavidus necator |
| Proteobacteria | Betaproteobacteria | Burkholderiales | Burkholderiaceae | Cupriavidus pinatubonensis |
| Proteobacteria | Betaproteobacteria | Burkholderiales | Burkholderiaceae | Cupriavidus taiwanensis |
| Proteobacteria | Betaproteobacteria | Burkholderiales | Burkholderiaceae | Pandoraea pnomenusa |
| Proteobacteria | Betaproteobacteria | Burkholderiales | Burkholderiaceae | Polynucleobacter necessarius |
| Proteobacteria | Betaproteobacteria | Burkholderiales | Burkholderiaceae | Ralstonia pickettii |
| Proteobacteria | Betaproteobacteria | Burkholderiales | Burkholderiaceae | Ralstonia solanacearum |
| Proteobacteria | Betaproteobacteria | Burkholderiales | Comamonadaceae | Acidovorax avenae |
| Proteobacteria | Betaproteobacteria | Burkholderiales | Comamonadaceae | Acidovorax citrulli |
| Proteobacteria | Betaproteobacteria | Burkholderiales | Comamonadaceae | Acidovorax ebreus |
| Proteobacteria | Betaproteobacteria | Burkholderiales | Comamonadaceae | Alicycliphilus denitrificans |
| Proteobacteria | Betaproteobacteria | Burkholderiales | Comamonadaceae | Comamonas testosteroni |
| Proteobacteria | Betaproteobacteria | Burkholderiales | Comamonadaceae | Delftia acidovorans |
| Proteobacteria | Betaproteobacteria | Burkholderiales | Comamonadaceae | Polaromonas naphthalenivorans |
| Proteobacteria | Betaproteobacteria | Burkholderiales | Comamonadaceae | Ramlibacter tataouinensis |
| Proteobacteria | Betaproteobacteria | Burkholderiales | Comamonadaceae | Rhodoferax ferrireducens |
| Proteobacteria | Betaproteobacteria | Burkholderiales | Comamonadaceae | Variovorax paradoxus |
| Proteobacteria | Betaproteobacteria | Burkholderiales | Comamonadaceae | Verminephrobacter eiseniae |
| Proteobacteria | Betaproteobacteria | Burkholderiales | Oxalobacteraceae | Collimonas fungivorans |
| Proteobacteria | Betaproteobacteria | Burkholderiales | Oxalobacteraceae | Herbaspirillum seropedicae |
| Proteobacteria | Betaproteobacteria | Burkholderiales | Oxalobacteraceae | Herminiimonas arsenicoxydans |
| Proteobacteria | Betaproteobacteria | Burkholderiales |  | Leptothrix cholodnii |
| Proteobacteria | Betaproteobacteria | Burkholderiales |  | Methylibium petroleiphilum |
| Proteobacteria | Betaproteobacteria | Burkholderiales |  | Rubrivivax gelatinosus |
| Proteobacteria | Betaproteobacteria | Burkholderiales |  | Thiomonas arsenitoxydans |
| Proteobacteria | Betaproteobacteria | Burkholderiales |  | Thiomonas intermedia |
| Proteobacteria | Betaproteobacteria | Hydrogenophilales | Hydrogenophilaceae | Thiobacillus denitrificans |
| Proteobacteria | Betaproteobacteria | Methylophilales | Methylophilaceae | Methylobacillus flagellatus |
| Proteobacteria | Betaproteobacteria | Methylophilales | Methylophilaceae | Methylotenera mobilis |
| Proteobacteria | Betaproteobacteria | Methylophilales | Methylophilaceae | Methylotenera versatilis |
| Proteobacteria | Betaproteobacteria | Methylophilales | Methylophilaceae | Methylovorus glucosotrophus |
| Proteobacteria | Betaproteobacteria | Neisseriales | Chromobacteriaceae | Chromobacterium violaceum |
| Proteobacteria | Betaproteobacteria | Neisseriales | Chromobacteriaceae | Laribacter hongkongensis |
| Proteobacteria | Betaproteobacteria | Neisseriales | Neisseriaceae | Neisseria gonorrhoeae |
| Proteobacteria | Betaproteobacteria | Neisseriales | Neisseriaceae | Neisseria lactamica |
| Proteobacteria | Betaproteobacteria | Neisseriales | Neisseriaceae | Neisseria meningitidis |
| Proteobacteria | Betaproteobacteria | Nitrosomonadales | Nitrosomonadaceae | Nitrosomonas europaea |
| Proteobacteria | Betaproteobacteria | Nitrosomonadales | Nitrosomonadaceae | Nitrosomonas eutropha |
| Proteobacteria | Betaproteobacteria | Nitrosomonadales | Nitrosomonadaceae | Nitrosospira multiformis |
| Proteobacteria | Betaproteobacteria | Rhodocyclales | Rhodocyclaceae | Azospira oryzae |
| Proteobacteria | Betaproteobacteria | Sulfuricellales | Sulfuricellaceae | Sulfuricella denitrificans |
| Proteobacteria | Betaproteobacteria |  | Kinetoplastibacterium blastocrithidii |  |
| Proteobacteria | Deltaproteobacteria | Bdellovibrionales | Bacteriovoracaceae | Bacteriovorax marinus |
| Proteobacteria | Deltaproteobacteria | Bdellovibrionales | Bdellovibrionaceae | Bdellovibrio bacteriovorus |
| Proteobacteria | Deltaproteobacteria | Bdellovibrionales | Bdellovibrionaceae | Bdellovibrio exovorus |
| Proteobacteria | Deltaproteobacteria | Desulfarculales | Desulfarculaceae | Desulfarculus baarsii |
| Proteobacteria | Deltaproteobacteria | Desulfobacterales | Desulfobacteraceae | Desulfatibacillum alkenivorans |
| Proteobacteria | Deltaproteobacteria | Desulfobacterales | Desulfobacteraceae | Desulfobacterium autotrophicum |
| Proteobacteria | Deltaproteobacteria | Desulfobacterales | Desulfobacteraceae | Desulfobacula toluolica |
| Proteobacteria | Deltaproteobacteria | Desulfobacterales | Desulfobulbaceae | Desulfobulbus propionicus |
| Proteobacteria | Deltaproteobacteria | Desulfobacterales | Desulfobulbaceae | Desulfocapsa sulfexigens |
| Proteobacteria | Deltaproteobacteria | Desulfobacterales | Desulfobulbaceae | Desulfurivibrio alkaliphilus |
| Proteobacteria | Deltaproteobacteria | Desulfovibrionales | Desulfohalobiaceae | Desulfohalobium retbaense |
| Proteobacteria | Deltaproteobacteria | Desulfovibrionales | Desulfomicrobiaceae | Desulfomicrobium baculatum |
| Proteobacteria | Deltaproteobacteria | Desulfovibrionales | Desulfovibrionaceae | Desulfovibrio aespoeensis |
| Proteobacteria | Deltaproteobacteria | Desulfovibrionales | Desulfovibrionaceae | Desulfovibrio africanus |
| Proteobacteria | Deltaproteobacteria | Desulfovibrionales | Desulfovibrionaceae | Desulfovibrio alaskensis |
| Proteobacteria | Deltaproteobacteria | Desulfovibrionales | Desulfovibrionaceae | Desulfovibrio desulfuricans |
| Proteobacteria | Deltaproteobacteria | Desulfovibrionales | Desulfovibrionaceae | Desulfovibrio gigas |
| Proteobacteria | Deltaproteobacteria | Desulfovibrionales | Desulfovibrionaceae | Desulfovibrio hydrothermalis |
| Proteobacteria | Deltaproteobacteria | Desulfovibrionales | Desulfovibrionaceae | Desulfovibrio magneticus |
| Proteobacteria | Deltaproteobacteria | Desulfovibrionales | Desulfovibrionaceae | Desulfovibrio salexigens |
| Proteobacteria | Deltaproteobacteria | Desulfovibrionales | Desulfovibrionaceae | Desulfovibrio vulgaris |
| Proteobacteria | Deltaproteobacteria | Desulfovibrionales | Desulfovibrionaceae | Lawsonia intracellularis |
| Proteobacteria | Deltaproteobacteria | Desulfurellales | Desulfurellaceae | Hippea maritima |
| Proteobacteria | Deltaproteobacteria | Desulfuromonadales | Geobacteraceae | Geobacter bemidjiensis |
| Proteobacteria | Deltaproteobacteria | Desulfuromonadales | Geobacteraceae | Geobacter daltonii |
| Proteobacteria | Deltaproteobacteria | Desulfuromonadales | Geobacteraceae | Geobacter lovleyi |
| Proteobacteria | Deltaproteobacteria | Desulfuromonadales | Geobacteraceae | Geobacter metallireducens |
| Proteobacteria | Deltaproteobacteria | Desulfuromonadales | Geobacteraceae | Geobacter sulfurreducens |
| Proteobacteria | Deltaproteobacteria | Desulfuromonadales | Geobacteraceae | Geobacter uraniireducens |
| Proteobacteria | Deltaproteobacteria | Desulfuromonadales | Pelobacteraceae | Pelobacter carbinolicus |
| Proteobacteria | Deltaproteobacteria | Desulfuromonadales | Pelobacteraceae | Pelobacter propionicus |
| Proteobacteria | Deltaproteobacteria | Myxococcales | Anaeromyxobacteraceae | Anaeromyxobacter dehalogenans |
| Proteobacteria | Deltaproteobacteria | Myxococcales | Cystobacteraceae | Stigmatella aurantiaca |
| Proteobacteria | Deltaproteobacteria | Myxococcales | Myxococcaceae | Corallococcus coralloides |
| Proteobacteria | Deltaproteobacteria | Myxococcales | Myxococcaceae | Myxococcus fulvus |
| Proteobacteria | Deltaproteobacteria | Myxococcales | Myxococcaceae | Myxococcus stipitatus |
| Proteobacteria | Deltaproteobacteria | Myxococcales | Myxococcaceae | Myxococcus xanthus |
| Proteobacteria | Deltaproteobacteria | Myxococcales | Kofleriaceae | Haliangium ochraceum |
| Proteobacteria | Deltaproteobacteria | Myxococcales | Polyangiaceae | Sorangium cellulosum |
| Proteobacteria | Deltaproteobacteria | Syntrophobacterales | Syntrophaceae | Desulfobacca acetoxidans |
| Proteobacteria | Deltaproteobacteria | Syntrophobacterales | Syntrophaceae | Syntrophus aciditrophicus |
| Proteobacteria | Deltaproteobacteria | Syntrophobacterales | Syntrophobacteraceae | Syntrophobacter fumaroxidans |
| Proteobacteria | Epsilonproteobacteria | Campylobacterales | Campylobacteraceae | Arcobacter butzleri |
| Proteobacteria | Epsilonproteobacteria | Campylobacterales | Campylobacteraceae | Arcobacter nitrofigilis |
| Proteobacteria | Epsilonproteobacteria | Campylobacterales | Campylobacteraceae | Campylobacter coli |
| Proteobacteria | Epsilonproteobacteria | Campylobacterales | Campylobacteraceae | Campylobacter concisus |
| Proteobacteria | Epsilonproteobacteria | Campylobacterales | Campylobacteraceae | Campylobacter curvus |
| Proteobacteria | Epsilonproteobacteria | Campylobacterales | Campylobacteraceae | Campylobacter fetus |
| Proteobacteria | Epsilonproteobacteria | Campylobacterales | Campylobacteraceae | Campylobacter hominis |
| Proteobacteria | Epsilonproteobacteria | Campylobacterales | Campylobacteraceae | Campylobacter jejuni |
| Proteobacteria | Epsilonproteobacteria | Campylobacterales | Campylobacteraceae | Campylobacter lari |
| Proteobacteria | Epsilonproteobacteria | Campylobacterales | Campylobacteraceae | Sulfurospirillum barnesii |
| Proteobacteria | Epsilonproteobacteria | Campylobacterales | Campylobacteraceae | Sulfurospirillum deleyianum |
| Proteobacteria | Epsilonproteobacteria | Campylobacterales | Helicobacteraceae | Helicobacter cetorum |
| Proteobacteria | Epsilonproteobacteria | Campylobacterales | Helicobacteraceae | Helicobacter hepaticus |
| Proteobacteria | Epsilonproteobacteria | Campylobacterales | Helicobacteraceae | Helicobacter mustelae |
| Proteobacteria | Epsilonproteobacteria | Campylobacterales | Helicobacteraceae | Helicobacter pylori |
| Proteobacteria | Epsilonproteobacteria | Campylobacterales | Helicobacteraceae | Sulfuricurvum kujiense |
| Proteobacteria | Epsilonproteobacteria | Campylobacterales | Helicobacteraceae | Sulfurimonas autotrophica |
| Proteobacteria | Epsilonproteobacteria | Campylobacterales | Helicobacteraceae | Sulfurimonas denitrificans |
| Proteobacteria | Epsilonproteobacteria | Campylobacterales | Nitratifractor salsuginis |  |
| Proteobacteria | Epsilonproteobacteria | Nautiliales | Nautiliaceae | Nautilia profundicola |
| Proteobacteria | Gammaproteobacteria | Acidithiobacillales | Acidithiobacillaceae | Acidithiobacillus caldus |
| Proteobacteria | Gammaproteobacteria | Acidithiobacillales | Acidithiobacillaceae | Acidithiobacillus ferrivorans |
| Proteobacteria | Gammaproteobacteria | Acidithiobacillales | Acidithiobacillaceae | Acidithiobacillus ferrooxidans |
| Proteobacteria | Gammaproteobacteria | Aeromonadales | Aeromonadaceae | Aeromonas hydrophila |
| Proteobacteria | Gammaproteobacteria | Aeromonadales | Aeromonadaceae | Aeromonas salmonicida |
| Proteobacteria | Gammaproteobacteria | Aeromonadales | Aeromonadaceae | Aeromonas veronii |
| Proteobacteria | Gammaproteobacteria | Aeromonadales | Aeromonadaceae | Tolumonas auensis |
| Proteobacteria | Gammaproteobacteria | Alteromonadales | Alteromonadaceae | Alteromonas macleodii |
| Proteobacteria | Gammaproteobacteria | Alteromonadales | Alteromonadaceae | Glaciecola nitratireducens |
| Proteobacteria | Gammaproteobacteria | Alteromonadales | Alteromonadaceae | Glaciecola psychrophila |
| Proteobacteria | Gammaproteobacteria | Alteromonadales | Alteromonadaceae | Marinobacter adhaerens |
| Proteobacteria | Gammaproteobacteria | Alteromonadales | Alteromonadaceae | Marinobacter hydrocarbonoclasticus |
| Proteobacteria | Gammaproteobacteria | Alteromonadales | Alteromonadaceae | Saccharophagus degradans |
| Proteobacteria | Gammaproteobacteria | Alteromonadales |  | Teredinibacter turnerae |
| Proteobacteria | Gammaproteobacteria | Alteromonadales | Colwelliaceae | Colwellia psychrerythraea |
| Proteobacteria | Gammaproteobacteria | Alteromonadales | Ferrimonadaceae | Ferrimonas balearica |
| Proteobacteria | Gammaproteobacteria | Alteromonadales | Idiomarinaceae | Idiomarina loihiensis |
| Proteobacteria | Gammaproteobacteria | Alteromonadales | Pseudoalteromonadaceae | Pseudoalteromonas atlantica |
| Proteobacteria | Gammaproteobacteria | Alteromonadales | Pseudoalteromonadaceae | Pseudoalteromonas haloplanktis |
| Proteobacteria | Gammaproteobacteria | Alteromonadales | Psychromonadaceae | Psychromonas ingrahamii |
| Proteobacteria | Gammaproteobacteria | Alteromonadales | Shewanellaceae | Shewanella amazonensis |
| Proteobacteria | Gammaproteobacteria | Alteromonadales | Shewanellaceae | Shewanella baltica |
| Proteobacteria | Gammaproteobacteria | Alteromonadales | Shewanellaceae | Shewanella denitrificans |
| Proteobacteria | Gammaproteobacteria | Alteromonadales | Shewanellaceae | Shewanella frigidimarina |
| Proteobacteria | Gammaproteobacteria | Alteromonadales | Shewanellaceae | Shewanella halifaxensis |
| Proteobacteria | Gammaproteobacteria | Alteromonadales | Shewanellaceae | Shewanella loihica |
| Proteobacteria | Gammaproteobacteria | Alteromonadales | Shewanellaceae | Shewanella oneidensis |
| Proteobacteria | Gammaproteobacteria | Alteromonadales | Shewanellaceae | Shewanella pealeana |
| Proteobacteria | Gammaproteobacteria | Alteromonadales | Shewanellaceae | Shewanella piezotolerans |
| Proteobacteria | Gammaproteobacteria | Alteromonadales | Shewanellaceae | Shewanella putrefaciens |
| Proteobacteria | Gammaproteobacteria | Alteromonadales | Shewanellaceae | Shewanella sediminis |
| Proteobacteria | Gammaproteobacteria | Alteromonadales | Shewanellaceae | Shewanella violacea |
| Proteobacteria | Gammaproteobacteria | Alteromonadales | Shewanellaceae | Shewanella woodyi |
| Proteobacteria | Gammaproteobacteria | Cardiobacteriales | Cardiobacteriaceae | Dichelobacter nodosus |
| Proteobacteria | Gammaproteobacteria | Chromatiales | Chromatiaceae | Allochromatium vinosum |
| Proteobacteria | Gammaproteobacteria | Chromatiales | Chromatiaceae | Nitrosococcus halophilus |
| Proteobacteria | Gammaproteobacteria | Chromatiales | Chromatiaceae | Nitrosococcus oceani |
| Proteobacteria | Gammaproteobacteria | Chromatiales | Chromatiaceae | Thiocystis violascens |
| Proteobacteria | Gammaproteobacteria | Chromatiales | Chromatiaceae | Thioflavicoccus mobilis |
| Proteobacteria | Gammaproteobacteria | Chromatiales | Ectothiorhodospiraceae | Alkalilimnicola ehrlichii |
| Proteobacteria | Gammaproteobacteria | Chromatiales | Ectothiorhodospiraceae | Halorhodospira halophila |
| Proteobacteria | Gammaproteobacteria | Chromatiales | Ectothiorhodospiraceae | Spiribacter salinus |
| Proteobacteria | Gammaproteobacteria | Chromatiales | Ectothiorhodospiraceae | Thioalkalivibrio nitratireducens |
| Proteobacteria | Gammaproteobacteria | Chromatiales | Ectothiorhodospiraceae | Thioalkalivibrio sulfidiphilus |
| Proteobacteria | Gammaproteobacteria | Chromatiales | Halothiobacillaceae | Halothiobacillus neapolitanus |
| Proteobacteria | Gammaproteobacteria | Enterobacteriales | Enterobacteriaceae | Buchnera aphidicola |
| Proteobacteria | Gammaproteobacteria | Enterobacteriales | Enterobacteriaceae | Citrobacter koseri |
| Proteobacteria | Gammaproteobacteria | Enterobacteriales | Enterobacteriaceae | Citrobacter rodentium |
| Proteobacteria | Gammaproteobacteria | Enterobacteriales | Enterobacteriaceae | Cronobacter sakazakii |
| Proteobacteria | Gammaproteobacteria | Enterobacteriales | Enterobacteriaceae | Cronobacter turicensis |
| Proteobacteria | Gammaproteobacteria | Enterobacteriales | Enterobacteriaceae | Dickeya dadantii |
| Proteobacteria | Gammaproteobacteria | Enterobacteriales | Enterobacteriaceae | Dickeya zeae |
| Proteobacteria | Gammaproteobacteria | Enterobacteriales | Enterobacteriaceae | Edwardsiella ictaluri |
| Proteobacteria | Gammaproteobacteria | Enterobacteriales | Enterobacteriaceae | Edwardsiella piscicida |
| Proteobacteria | Gammaproteobacteria | Enterobacteriales | Enterobacteriaceae | Edwardsiella tarda |
| Proteobacteria | Gammaproteobacteria | Enterobacteriales | Enterobacteriaceae | Enterobacter aerogenes |
| Proteobacteria | Gammaproteobacteria | Enterobacteriales | Enterobacteriaceae | Enterobacter asburiae |
| Proteobacteria | Gammaproteobacteria | Enterobacteriales | Enterobacteriaceae | Enterobacter cloacae |
| Proteobacteria | Gammaproteobacteria | Enterobacteriales | Enterobacteriaceae | Enterobacter lignolyticus |
| Proteobacteria | Gammaproteobacteria | Enterobacteriales | Enterobacteriaceae | Erwinia amylovora |
| Proteobacteria | Gammaproteobacteria | Enterobacteriales | Enterobacteriaceae | Erwinia billingiae |
| Proteobacteria | Gammaproteobacteria | Enterobacteriales | Enterobacteriaceae | Erwinia pyrifoliae |
| Proteobacteria | Gammaproteobacteria | Enterobacteriales | Enterobacteriaceae | Erwinia tasmaniensis |
| Proteobacteria | Gammaproteobacteria | Enterobacteriales | Enterobacteriaceae | Escherichia coli |
| Proteobacteria | Gammaproteobacteria | Enterobacteriales | Enterobacteriaceae | Escherichia fergusonii |
| Proteobacteria | Gammaproteobacteria | Enterobacteriales | Enterobacteriaceae | Klebsiella oxytoca |
| Proteobacteria | Gammaproteobacteria | Enterobacteriales | Enterobacteriaceae | Klebsiella pneumoniae |
| Proteobacteria | Gammaproteobacteria | Enterobacteriales | Enterobacteriaceae | Klebsiella variicola |
| Proteobacteria | Gammaproteobacteria | Enterobacteriales | Enterobacteriaceae | Morganella morganii |
| Proteobacteria | Gammaproteobacteria | Enterobacteriales | Enterobacteriaceae | Pantoea ananatis |
| Proteobacteria | Gammaproteobacteria | Enterobacteriales | Enterobacteriaceae | Pantoea vagans |
| Proteobacteria | Gammaproteobacteria | Enterobacteriales | Enterobacteriaceae | Pectobacterium atrosepticum |
| Proteobacteria | Gammaproteobacteria | Enterobacteriales | Enterobacteriaceae | Pectobacterium carotovorum |
| Proteobacteria | Gammaproteobacteria | Enterobacteriales | Enterobacteriaceae | Pectobacterium wasabiae |
| Proteobacteria | Gammaproteobacteria | Enterobacteriales | Enterobacteriaceae | Photorhabdus asymbiotica |
| Proteobacteria | Gammaproteobacteria | Enterobacteriales | Enterobacteriaceae | Photorhabdus luminescens |
| Proteobacteria | Gammaproteobacteria | Enterobacteriales | Enterobacteriaceae | Proteus mirabilis |
| Proteobacteria | Gammaproteobacteria | Enterobacteriales | Enterobacteriaceae | Providencia stuartii |
| Proteobacteria | Gammaproteobacteria | Enterobacteriales | Enterobacteriaceae | Rahnella aquatilis |
| Proteobacteria | Gammaproteobacteria | Enterobacteriales | Enterobacteriaceae | Raoultella ornithinolytica |
| Proteobacteria | Gammaproteobacteria | Enterobacteriales | Enterobacteriaceae | Salmonella bongori |
| Proteobacteria | Gammaproteobacteria | Enterobacteriales | Enterobacteriaceae | Salmonella enterica |
| Proteobacteria | Gammaproteobacteria | Enterobacteriales | Enterobacteriaceae | Serratia liquefaciens |
| Proteobacteria | Gammaproteobacteria | Enterobacteriales | Enterobacteriaceae | Serratia marcescens |
| Proteobacteria | Gammaproteobacteria | Enterobacteriales | Enterobacteriaceae | Serratia plymuthica |
| Proteobacteria | Gammaproteobacteria | Enterobacteriales | Enterobacteriaceae | Serratia proteamaculans |
| Proteobacteria | Gammaproteobacteria | Enterobacteriales | Enterobacteriaceae | Serratia symbiotica |
| Proteobacteria | Gammaproteobacteria | Enterobacteriales | Enterobacteriaceae | Shigella boydii |
| Proteobacteria | Gammaproteobacteria | Enterobacteriales | Enterobacteriaceae | Shigella dysenteriae |
| Proteobacteria | Gammaproteobacteria | Enterobacteriales | Enterobacteriaceae | Shigella flexneri |
| Proteobacteria | Gammaproteobacteria | Enterobacteriales | Enterobacteriaceae | Shigella sonnei |
| Proteobacteria | Gammaproteobacteria | Enterobacteriales | Enterobacteriaceae | Shimwellia blattae |
| Proteobacteria | Gammaproteobacteria | Enterobacteriales | Enterobacteriaceae | Sodalis glossinidius |
| Proteobacteria | Gammaproteobacteria | Enterobacteriales | Enterobacteriaceae | Wigglesworthia glossinidia |
| Proteobacteria | Gammaproteobacteria | Enterobacteriales | Enterobacteriaceae | Xenorhabdus bovienii |
| Proteobacteria | Gammaproteobacteria | Enterobacteriales | Enterobacteriaceae | Xenorhabdus nematophila |
| Proteobacteria | Gammaproteobacteria | Enterobacteriales | Enterobacteriaceae | Yersinia enterocolitica |
| Proteobacteria | Gammaproteobacteria | Enterobacteriales | Enterobacteriaceae | Yersinia pestis |
| Proteobacteria | Gammaproteobacteria | Legionellales | Coxiellaceae | Coxiella burnetii |
| Proteobacteria | Gammaproteobacteria | Legionellales | Legionellaceae | Legionella longbeachae |
| Proteobacteria | Gammaproteobacteria | Legionellales | Legionellaceae | Legionella pneumophila |
| Proteobacteria | Gammaproteobacteria | Methylococcales | Methylococcaceae | Methylococcus capsulatus |
| Proteobacteria | Gammaproteobacteria | Methylococcales | Methylococcaceae | Methylomicrobium alcaliphilum |
| Proteobacteria | Gammaproteobacteria | Methylococcales | Methylococcaceae | Methylomonas methanica |
| Proteobacteria | Gammaproteobacteria | Oceanospirillales | Alcanivoracaceae | Alcanivorax borkumensis |
| Proteobacteria | Gammaproteobacteria | Oceanospirillales | Alcanivoracaceae | Alcanivorax dieselolei |
| Proteobacteria | Gammaproteobacteria | Oceanospirillales | Alcanivoracaceae | Kangiella koreensis |
| Proteobacteria | Gammaproteobacteria | Oceanospirillales | Hahellaceae | Hahella chejuensis |
| Proteobacteria | Gammaproteobacteria | Oceanospirillales | Halomonadaceae | Chromohalobacter salexigens |
| Proteobacteria | Gammaproteobacteria | Oceanospirillales | Halomonadaceae | Halomonas elongata |
| Proteobacteria | Gammaproteobacteria | Oceanospirillales | Oceanospirillaceae | Marinomonas mediterranea |
| Proteobacteria | Gammaproteobacteria | Oceanospirillales | Oceanospirillaceae | Marinomonas posidonica |
| Proteobacteria | Gammaproteobacteria | Oceanospirillales | Oceanospirillaceae | Thalassolituus oleivorans |
| Proteobacteria | Gammaproteobacteria | Pasteurellales | Pasteurellaceae | Actinobacillus pleuropneumoniae |
| Proteobacteria | Gammaproteobacteria | Pasteurellales | Pasteurellaceae | Actinobacillus succinogenes |
| Proteobacteria | Gammaproteobacteria | Pasteurellales | Pasteurellaceae | Actinobacillus suis |
| Proteobacteria | Gammaproteobacteria | Pasteurellales | Pasteurellaceae | Aggregatibacter actinomycetemcomitans |
| Proteobacteria | Gammaproteobacteria | Pasteurellales | Pasteurellaceae | Aggregatibacter aphrophilus |
| Proteobacteria | Gammaproteobacteria | Pasteurellales | Pasteurellaceae | Bibersteinia trehalosi |
| Proteobacteria | Gammaproteobacteria | Pasteurellales | Pasteurellaceae | Gallibacterium anatis |
| Proteobacteria | Gammaproteobacteria | Pasteurellales | Pasteurellaceae | Haemophilus ducreyi |
| Proteobacteria | Gammaproteobacteria | Pasteurellales | Pasteurellaceae | Haemophilus influenzae |
| Proteobacteria | Gammaproteobacteria | Pasteurellales | Pasteurellaceae | Haemophilus parainfluenzae |
| Proteobacteria | Gammaproteobacteria | Pasteurellales | Pasteurellaceae | Haemophilus parasuis |
| Proteobacteria | Gammaproteobacteria | Pasteurellales | Pasteurellaceae | Histophilus somni |
| Proteobacteria | Gammaproteobacteria | Pasteurellales | Pasteurellaceae | Mannheimia haemolytica |
| Proteobacteria | Gammaproteobacteria | Pasteurellales | Pasteurellaceae | Pasteurella multocida |
| Proteobacteria | Gammaproteobacteria | Pseudomonadales | Moraxellaceae | Acinetobacter baumannii |
| Proteobacteria | Gammaproteobacteria | Pseudomonadales | Moraxellaceae | Acinetobacter calcoaceticus |
| Proteobacteria | Gammaproteobacteria | Pseudomonadales | Moraxellaceae | Acinetobacter oleivorans |
| Proteobacteria | Gammaproteobacteria | Pseudomonadales | Moraxellaceae | Moraxella catarrhalis |
| Proteobacteria | Gammaproteobacteria | Pseudomonadales | Moraxellaceae | Psychrobacter arcticus |
| Proteobacteria | Gammaproteobacteria | Pseudomonadales | Moraxellaceae | Psychrobacter cryohalolentis |
| Proteobacteria | Gammaproteobacteria | Pseudomonadales | Pseudomonadaceae | Azotobacter vinelandii |
| Proteobacteria | Gammaproteobacteria | Pseudomonadales | Pseudomonadaceae | Cellvibrio japonicus |
| Proteobacteria | Gammaproteobacteria | Pseudomonadales | Pseudomonadaceae | Pseudomonas aeruginosa |
| Proteobacteria | Gammaproteobacteria | Pseudomonadales | Pseudomonadaceae | Pseudomonas mendocina |
| Proteobacteria | Gammaproteobacteria | Pseudomonadales | Pseudomonadaceae | Pseudomonas resinovorans |
| Proteobacteria | Gammaproteobacteria | Pseudomonadales | Pseudomonadaceae | Pseudomonas brassicacearum |
| Proteobacteria | Gammaproteobacteria | Pseudomonadales | Pseudomonadaceae | Pseudomonas entomophila |
| Proteobacteria | Gammaproteobacteria | Pseudomonadales | Pseudomonadaceae | Pseudomonas fluorescens |
| Proteobacteria | Gammaproteobacteria | Pseudomonadales | Pseudomonadaceae | Pseudomonas poae |
| Proteobacteria | Gammaproteobacteria | Pseudomonadales | Pseudomonadaceae | Pseudomonas protegens |
| Proteobacteria | Gammaproteobacteria | Pseudomonadales | Pseudomonadaceae | Pseudomonas denitrificans |
| Proteobacteria | Gammaproteobacteria | Pseudomonadales | Pseudomonadaceae | Pseudomonas fulva |
| Proteobacteria | Gammaproteobacteria | Pseudomonadales | Pseudomonadaceae | Pseudomonas monteilii |
| Proteobacteria | Gammaproteobacteria | Pseudomonadales | Pseudomonadaceae | Pseudomonas putida |
| Proteobacteria | Gammaproteobacteria | Pseudomonadales | Pseudomonadaceae | Pseudomonas stutzeri |
| Proteobacteria | Gammaproteobacteria | Pseudomonadales | Pseudomonadaceae | Pseudomonas syringae |
| Proteobacteria | Gammaproteobacteria | Pseudomonadales | Pseudomonadaceae | Pseudomonas savastanoi |
| Proteobacteria | Gammaproteobacteria | Thiotrichales | Francisellaceae | Francisella tularensis |
| Proteobacteria | Gammaproteobacteria | Thiotrichales | Piscirickettsiaceae | Methylophaga frappieri |
| Proteobacteria | Gammaproteobacteria | Thiotrichales | Piscirickettsiaceae | Methylophaga nitratireducenticrescens |
| Proteobacteria | Gammaproteobacteria | Thiotrichales | Piscirickettsiaceae | Thioalkalimicrobium cyclicum |
| Proteobacteria | Gammaproteobacteria | Thiotrichales | Piscirickettsiaceae | Thiomicrospira crunogena |
| Proteobacteria | Gammaproteobacteria |  | Simiduia agarivorans |  |
| Proteobacteria | Gammaproteobacteria | Vibrionales | Vibrionaceae | Aliivibrio fischeri |
| Proteobacteria | Gammaproteobacteria | Vibrionales | Vibrionaceae | Aliivibrio salmonicida |
| Proteobacteria | Gammaproteobacteria | Vibrionales | Vibrionaceae | Photobacterium profundum |
| Proteobacteria | Gammaproteobacteria | Vibrionales | Vibrionaceae | Vibrio anguillarum |
| Proteobacteria | Gammaproteobacteria | Vibrionales | Vibrionaceae | Vibrio cholerae |
| Proteobacteria | Gammaproteobacteria | Vibrionales | Vibrionaceae | Vibrio furnissii |
| Proteobacteria | Gammaproteobacteria | Vibrionales | Vibrionaceae | Vibrio alginolyticus |
| Proteobacteria | Gammaproteobacteria | Vibrionales | Vibrionaceae | Vibrio campbellii |
| Proteobacteria | Gammaproteobacteria | Vibrionales | Vibrionaceae | Vibrio parahaemolyticus |
| Proteobacteria | Gammaproteobacteria | Vibrionales | Vibrionaceae | Vibrio nigripulchritudo |
| Proteobacteria | Gammaproteobacteria | Vibrionales | Vibrionaceae | Vibrio splendidus |
| Proteobacteria | Gammaproteobacteria | Vibrionales | Vibrionaceae | Vibrio vulnificus |
| Proteobacteria | Gammaproteobacteria | Xanthomonadales | Xanthomonadaceae | Frateuria aurantia |
| Proteobacteria | Gammaproteobacteria | Xanthomonadales | Xanthomonadaceae | Pseudoxanthomonas spadix |
| Proteobacteria | Gammaproteobacteria | Xanthomonadales | Xanthomonadaceae | Pseudoxanthomonas suwonensis |
| Proteobacteria | Gammaproteobacteria | Xanthomonadales | Xanthomonadaceae | Rhodanobacter denitrificans |
| Proteobacteria | Gammaproteobacteria | Xanthomonadales | Xanthomonadaceae | Stenotrophomonas maltophilia |
| Proteobacteria | Gammaproteobacteria | Xanthomonadales | Xanthomonadaceae | Xanthomonas albilineans |
| Proteobacteria | Gammaproteobacteria | Xanthomonadales | Xanthomonadaceae | Xanthomonas alfalfae |
| Proteobacteria | Gammaproteobacteria | Xanthomonadales | Xanthomonadaceae | Xanthomonas campestris |
| Proteobacteria | Gammaproteobacteria | Xanthomonadales | Xanthomonadaceae | Xanthomonas citri |
| Proteobacteria | Gammaproteobacteria | Xanthomonadales | Xanthomonadaceae | Xanthomonas fuscans |
| Proteobacteria | Gammaproteobacteria | Xanthomonadales | Xanthomonadaceae | Xanthomonas euvesicatoria |
| Proteobacteria | Gammaproteobacteria | Xanthomonadales | Xanthomonadaceae | Xanthomonas oryzae |
| Proteobacteria | Gammaproteobacteria | Xanthomonadales | Xanthomonadaceae | Xylella fastidiosa |
| Spirochaetes | Spirochaetia | Spirochaetales | Brachyspiraceae | Brachyspira hyodysenteriae |
| Spirochaetes | Spirochaetia | Spirochaetales | Brachyspiraceae | Brachyspira intermedia |
| Spirochaetes | Spirochaetia | Spirochaetales | Brachyspiraceae | Brachyspira murdochii |
| Spirochaetes | Spirochaetia | Spirochaetales | Brachyspiraceae | Brachyspira pilosicoli |
| Spirochaetes | Spirochaetia | Spirochaetales | Leptospiraceae | Leptospira borgpetersenii |
| Spirochaetes | Spirochaetia | Spirochaetales | Leptospiraceae | Leptospira interrogans |
| Spirochaetes | Spirochaetia | Spirochaetales | Spirochaetaceae | Sphaerochaeta globosa |
| Spirochaetes | Spirochaetia | Spirochaetales | Spirochaetaceae | Sphaerochaeta pleomorpha |
| Spirochaetes | Spirochaetia | Spirochaetales | Spirochaetaceae | Spirochaeta africana |
| Spirochaetes | Spirochaetia | Spirochaetales | Spirochaetaceae | Spirochaeta smaragdinae |
| Spirochaetes | Spirochaetia | Spirochaetales | Spirochaetaceae | Spirochaeta thermophila |
| Spirochaetes | Spirochaetia | Spirochaetales | Spirochaetaceae | Treponema azotonutricium |
| Spirochaetes | Spirochaetia | Spirochaetales | Spirochaetaceae | Treponema brennaborense |
| Spirochaetes | Spirochaetia | Spirochaetales | Spirochaetaceae | Treponema denticola |
| Spirochaetes | Spirochaetia | Spirochaetales | Spirochaetaceae | Treponema pedis |
| Spirochaetes | Spirochaetia | Spirochaetales | Spirochaetaceae | Treponema primitia |
| Spirochaetes | Spirochaetia | Spirochaetales | Spirochaetaceae | Treponema succinifaciens |
| Synergistetes | Synergistia | Synergistales | Synergistaceae | Aminobacterium colombiense |
| Synergistetes | Synergistia | Synergistales | Synergistaceae | Anaerobaculum mobile |
| Synergistetes | Synergistia | Synergistales | Synergistaceae | Fretibacterium fastidiosum |
| Synergistetes | Synergistia | Synergistales | Synergistaceae | Thermanaerovibrio acidaminovorans |
| Tenericutes | Mollicutes | Acholeplasmatales | Acholeplasmataceae | Acholeplasma brassicae |
| Tenericutes | Mollicutes | Acholeplasmatales | Acholeplasmataceae | Acholeplasma laidlawii |
| Tenericutes | Mollicutes | Acholeplasmatales | Acholeplasmataceae | Acholeplasma palmae |
| Tenericutes | Mollicutes | Entomoplasmatales | Entomoplasmataceae | Mesoplasma florum |
| Tenericutes | Mollicutes | Entomoplasmatales | Spiroplasmataceae | Spiroplasma apis |
| Tenericutes | Mollicutes | Entomoplasmatales | Spiroplasmataceae | Spiroplasma chrysopicola |
| Tenericutes | Mollicutes | Entomoplasmatales | Spiroplasmataceae | Spiroplasma diminutum |
| Tenericutes | Mollicutes | Entomoplasmatales | Spiroplasmataceae | Spiroplasma syrphidicola |
| Tenericutes | Mollicutes | Entomoplasmatales | Spiroplasmataceae | Spiroplasma taiwanense |
| Tenericutes | Mollicutes | Mycoplasmatales | Mycoplasmataceae | Mycoplasma agalactiae |
| Tenericutes | Mollicutes | Mycoplasmatales | Mycoplasmataceae | Mycoplasma bovis |
| Tenericutes | Mollicutes | Mycoplasmatales | Mycoplasmataceae | Mycoplasma conjunctivae |
| Tenericutes | Mollicutes | Mycoplasmatales | Mycoplasmataceae | Mycoplasma crocodyli |
| Tenericutes | Mollicutes | Mycoplasmatales | Mycoplasmataceae | Mycoplasma cynos |
| Tenericutes | Mollicutes | Mycoplasmatales | Mycoplasmataceae | Mycoplasma fermentans |
| Tenericutes | Mollicutes | Mycoplasmatales | Mycoplasmataceae | Mycoplasma gallisepticum |
| Tenericutes | Mollicutes | Mycoplasmatales | Mycoplasmataceae | Mycoplasma genitalium |
| Tenericutes | Mollicutes | Mycoplasmatales | Mycoplasmataceae | Mycoplasma hyopneumoniae |
| Tenericutes | Mollicutes | Mycoplasmatales | Mycoplasmataceae | Mycoplasma mobile |
| Tenericutes | Mollicutes | Mycoplasmatales | Mycoplasmataceae | Mycoplasma leachii |
| Tenericutes | Mollicutes | Mycoplasmatales | Mycoplasmataceae | Mycoplasma mycoides |
| Tenericutes | Mollicutes | Mycoplasmatales | Mycoplasmataceae | Mycoplasma penetrans |
| Tenericutes | Mollicutes | Mycoplasmatales | Mycoplasmataceae | Mycoplasma putrefaciens |
| Tenericutes | Mollicutes | Mycoplasmatales | Mycoplasmataceae | Mycoplasma synoviae |
| Tenericutes | Mollicutes | Mycoplasmatales | Mycoplasmataceae | Mycoplasma wenyonii |
| Tenericutes | Mollicutes | Mycoplasmatales | Mycoplasmataceae | Ureaplasma parvum |
| Tenericutes | Mollicutes | Mycoplasmatales | Mycoplasmataceae | Ureaplasma urealyticum |
| Thermodesulfobacteria | Thermodesulfobacteria | Thermodesulfobacteriales | Thermodesulfobacteriaceae | Thermodesulfobacterium geofontis |
| Thermotogae | Thermotogae | Thermotogales | Thermotogaceae | Fervidobacterium pennivorans |
| Thermotogae | Thermotogae | Thermotogales | Thermotogaceae | Kosmotoga olearia |
| Thermotogae | Thermotogae | Thermotogales | Thermotogaceae | Marinitoga piezophila |
| Thermotogae | Thermotogae | Thermotogales | Thermotogaceae | Petrotoga mobilis |
| Thermotogae | Thermotogae | Thermotogales | Thermotogaceae | Thermosipho africanus |
| Thermotogae | Thermotogae | Thermotogales | Thermotogaceae | Thermosipho melanesiensis |
| Thermotogae | Thermotogae | Thermotogales | Thermotogaceae | Thermotoga thermarum |
|  |  |  |  | Thermobaculum terrenum |

Figure S1.1 – S1.3 show how species diversity is distributed among genra (S1.1), families (S1.2) and orders (S1.3) for abundant skin taxa, all skin taxa, and within our world database. Tables S1.3 and S1.4 provide further taxonomic detail, listing each species within our set of abundant skin microbes (185 species) and within our full database (971 species).

*Supplementary Information II: Phylogenetically Corrected Abundant vs. Rare Analysis*

**Table S2.1** p-values for our phylogenetically corrected and naïve regression models for each binary trait. Traits shown in purple are significantly different between abundant and rare taxa, regardless of whether phylogeny is accounted for. Traits shown in red are only significantly different when phylogeny is accounted for. Traits shown in black are never significantly different.

| **Property** | **p-value**  **(phylogenetic correction)** | **p-value**  **(naive)** |
| --- | --- | --- |
| spore | 0.0018971 | 0.0000066 |
| urease | 0.0096407 | 0.0198115 |
| catalase | 0.0234165 | 0.0010955 |
| H2S.production | 0.0250826 | 0.0312478 |
| aesculin.hydrolysis | 0.0394342 | 0.003431 |
| nitrate.reduction | 0.0468589 | 0.1476493 |
| tellurite.reductase | 0.0996023 | 0.4986451 |
| acid.phosphatase | 0.11107 | 0.3427225 |
| indole.production | 0.1226892 | 0.3551695 |
| gelatinase | 0.1565554 | 0.0402519 |
| pyrazinamidase | 0.1603656 | 0.0062074 |
| pigment | 0.1662447 | 0.1081922 |
| alkaline.phosphatase | 0.2801919 | 0.0041473 |
| oxidase | 0.3169469 | 0.9043971 |
| alpha.galactosidase | 0.3705846 | 0.3880936 |
| arylsulfatase | 0.6665642 | 0.4942192 |
| methane.production | 0.9902427 | 0.7404402 |

**Table S2.2** p-values for our phylogenetically corrected and naïve regression models for each qualitative trait. Traits shown in purple are significantly different between abundant and rare taxa, regardless of whether phylogeny is accounted for. Traits shown in blue are only significantly different when phylogeny is not accounted for. Traits shown in black are never significantly different.

| **Property** | **p-value**  **(phylogenetic correction)** | **p-value**  **(naive)** |
| --- | --- | --- |
| oxygen | 0 | 0.0000038 |
| gram | 0.0000081 | 0.0000071 |
| motility | 0.0000206 | 0.0002365 |
| shape | 0.112307 | 0.0003253 |
| aggregation | 0.3888261 | 0.8166577 |

**Table S2.3** p-values for our phylogenetically corrected and naïve regression models for carbon substrate use. Uses shown in purple are significantly different between abundant and rare taxa, regardless of whether phylogeny is accounted for. Uses shown in blue are only significantly different when phylogeny is not accounted for. Uses shown in red are only significantly different between abundant and rare taxa when phylogeny is accounted for. All remaining carbon substrates showed no difference between abundant and rare taxa, regardless of whether or not phylogenetic correction was performed.

| **Property** | **p-value**  **(phylogenetic correction)** | **p-value**  **(naive)** |
| --- | --- | --- |
| succinate | 0.0023402 | 0.0002057 |
| xylose | 0.0625046 | 0.0002321 |
| acetate | 0.0071451 | 0.0015359 |
| malate | 0.0473865 | 0.0026159 |
| serine | 0.0124822 | 0.0064257 |
| proline | 0.0408959 | 0.0084473 |
| glutamate | 0.0231213 | 0.0097892 |
| gluconate | 0.0236054 | 0.0268427 |
| cellobiose | 0.2114483 | 0.0004844 |
| glucose | 0.0704883 | 0.0035059 |
| alanine | 0.1006266 | 0.0048108 |
| citrate | 0.1013365 | 0.0051717 |
| formate | 0.0830485 | 0.0138382 |
| malonate | 0.0808464 | 0.0149082 |
| valerate | 0.128235 | 0.0263087 |
| leucine | 0.5292001 | 0.0305671 |
| putrescine | 0.4977342 | 0.0309385 |
| aspartate | 0.3482498 | 0.0332374 |
| glycine | 0.9973798 | 0.0396153 |
| asparagine | 0.0929159 | 0.047413 |
| pyruvate | 0.2785002 | 0.0490681 |
| 2-ketogluconate | 0.0461109 | 0.1537924 |

*Supplementary Information III: Skin Bacteria vs. the World* (*All Taxa*)

In the main text, we considered comparisons between abundant skin taxa and the bacteria in the world in general. Here, we extend our analysis to consider comparisons between all skin taxa and the bacteria in the world.

**Figure S3.1** Qualitative trait comparison for all taxa (>0.001% of reads in at least one sample; see also Supplementary Information I). (A) Proportion of taxa with a specific, qualitative trait in skin microbial communities (x-axis) versus the world as a whole (y-axis). Filled symbols represent traits that are significantly different in skin environments; open circles represent traits that are not; marker size reflects significance. (B) Plots of trait proportions among skin bacteria (pink) and world bacteria (green). Open red circles denote traits that are overrepresented on skin; filled green circles denote traits that are overrepresented in the world (underrepresented on skin).

Figure S3.1 shows results for qualitative traits. In keeping with trends observed across abundant skin taxa (see main text, Figure 3A), we find that spore formation, acid and alkaline phosphatase, oxidase, pigment production and catalase activity are underrepresented among skin bacteria. In addition, however, analysis of the full skin microbiome shows that aesculin hydrolysis and α-galactosidase are also underrepresented on skin, while hydrogen sulfide and indole are slightly, but significantly overrepresented. For categorical traits, we again see broad similarity between results for abundant taxa and the skin community at large (see main text, Figure 3B). Skin species are less likely to be aerobic and more likely to be facultative as compared to bacteria from the entire world. In addition, however, when rarer skin species are included in the analysis, we find that skin species are also more likely to be anaerobic or microaerophilic. For the full community, skin bacteria are more likely to exhibit type iv pilli and flagella and, correspondingly, more likely to be motile (note that this is slightly different from results for abundant bactera) than world bacteria. As with abundant species, all skin taxa are more likely to be coccobacilli or cocci and less likely to be rods as compared to world bacteria. However, in contrast to abundant skin taxa, which were less likely to be spirilla, species from the broader skin community are more likely to be spirilla. Skin bacteria are more likely to be Gram-negative as compared to bacteria from other environments. Finally, skin bacteria are less likely to grow in chains, preferring to aggregate as clumps or grow singly, instead.

Figure S3.2 compares quantitative traits among world and all skin bacteria. Skin bacteria from the full community have more difficulty at high pH, a lower pH optimum and a smaller range of pH values over which growth can occur. They prefer warmer temperatures, can tolerate warmer temperatures, and have more difficulty at cold temperatures. Additionally, they have a smaller pH range. Finally, they are less resilient to hypotonic conditions and have a lower GC content (see Figure S3.3).

**Figure S3.2** Boxplots comparing quantitative traits among skin bacteria (pink) and bacteria from the world in general (green) for all skin microbes (>0.001% of reads in at least one sample; see also Supplemental Information I). Red stars are used to denote significant differences between a trait value in the world versus on skin. Box width indicates the relative number of microbes used for the comparison.

**Figure S3.3** Boxplots comparing GC percent among skin bacteria (pink) and bacteria from the world in general (green). Blue stars are used to denote significant differences between a trait value in the world versus on skin. Box width indicates the relative number of microbes used for the comparison. Analyses are shown for (A) all skin bacteria (>0.001% of reads in at least one sample) and (B) abundant skin taxa (>0.1% of reads in at least one sample).

*Supplementary Information IV: Skin Bacteria vs. the World* (*Accounting for Phylogeny*)

**Figure S4.1** Proportion of taxa with a specific, qualitative trait amongst abundant skin microbes (x-axis, >0.1% of reads in at least one sample) versus the world as a whole (y-axis) for (A) *Actinobacteria*, (B) *Firmicutes*. (C) *Proteobacteria* and (D) *Bacteroidetes*. Filled symbols represent traits that are significantly different in skin environments; open circles represent traits that are not; marker size reflects significance.

**Figure S4.2** Proportion of taxa with a specific, qualitative trait amongst all skin microbes (x-axis, >0.001% of reads in at least one sample) versus the world as a whole (y-axis) for (A) *Actinobacteria*, (B) *Firmicutes*. (C) *Proteobacteria* and (D) *Bacteroidetes*. Filled symbols represent traits that are significantly different in skin environments; open circles represent traits that are not; marker size reflects significance.

Figures S4.1 and S4.2 show a comparison of binary traits amongst skin and world *Actinobacteria*, *Firmicutes*, *Proteobacteria*, and *Bacteroidetes* for abundant skin taxa (>0.1% of reads in at least one sample) and all skin taxa (>0.001% of reads in at least one sample) respectively. Tables S4.1, S4.2 and S4.3 summarize these results, along with the results in Figure S3.1 and in Figure 3 and Table 2 from the main text.

**Table S4.1** Significant differences between skin microbes and microbes from the world for binary traits. ‘W’ indicates that a trait that is signficantly overrepresented among world microbes; ‘S’ indicates that it is significantly overrepresented among skin microbes; ‘n/a’ indicates that a trait was not reported from a particular environment. Outcomes for abundant skin taxa (>0.1% of reads in at least one sample) are stated first (black); outcomes for the full skin community (>0.001% of reads in at least one sample) are stated second (grey). One star indicates that $0.67<{\rho_{w}}/{\rho_{s}} \mathrm{or} {\rho_{w}}/{\rho_{s}}>1.5$ and $\rho_{w}>0.05$, where $\rho_{w}$ and $\rho_{s}$ are the proportions of taxa exhibiting a particular trait in the world and on skin respectively. Two stars indicate that $0.5<{\rho_{w}}/{\rho_{s}} \mathrm{or} {\rho_{w}}/{\rho_{s}}>2$ and $\rho_{w}>0.05$.

|  | **Bacteria** | | **Actinobacteria** | | **Firmicutes** | | **Proteobacteria** | | **Bacteroidetes** | |
| --- | --- | --- | --- | --- | --- | --- | --- | --- | --- | --- |
| general | | | | | | | | | | |
| spore | W** | W | W** | W* | W** | W* | W |  |  |  |
| pigment | W | W |  |  |  | W** | W | W* |  | W |
| nitrate reduction |  |  |  |  |  |  |  |  |  |  |
| enzyme activity | | | | | | | | | | |
| acid phosphatase | W** | W** |  |  |  | W** | W** | W** | W** | W* |
| alkaline phosphatase | W* | W** |  |  |  |  | W** | W** |  |  |
| aesulin hydrolysis |  | W |  |  |  |  |  | W* |  |  |
| α-galactosidase |  | W* |  |  |  |  | W | W |  |  |
| arylsulfatase |  |  | W |  |  | |  |  | n/a | |
| catalase | W | W* | S |  | W** | W** |  | W |  | W |
| oxidase | W* | W* |  |  | W** | W** |  | W* | W** | W* |
| urease |  |  |  |  |  |  |  |  | W** |  |
| gelatinase |  |  |  |  |  |  |  |  |  |  |
| pyrazinamidase |  |  | S** | S** |  | |  |  | n/a | |
| tellurite reductase |  |  |  |  |  | | n/a | | n/a | |
| gas production | | | | | | | | | | |
| H_2_S production |  | S* |  |  |  |  |  | S** | W |  |
| indole production |  |  | W | W | W |  |  | S |  |  |
| methane production |  |  | n/a | |  | |  |  | n/a | |

**Table S4.2** The proportion of taxa exhibiting a particular binary trait in the world ($\rho_{w}$), among abundant (>0.1% reads) skin organisms ($\rho_{s}$), and the ratio of the two (${\rho_{w}}/{\rho_{s}}$).

|  | Bacteria | | | Actinobacteria | | | Firmicutes | | | Proteobacteria | | | Bacteroides | | |
| --- | --- | --- | --- | --- | --- | --- | --- | --- | --- | --- | --- | --- | --- | --- | --- |
|  | *ρ_s_* | *ρ_w_* | *ρ_w_/ρ_s_* | *ρ_s_* | *ρ_w_* | *ρ_w_/ρ_s_* | *ρ_s_* | *ρ_w_* | *ρ_w_/ρ_s_* | *ρ_s_* | *ρ_w_* | *ρ_w_/ρ_s_* | *ρ_s_* | *ρ_w_* | *ρ_w_/ρ_s_* |
| general | | | | | | | | | | | | | | | |
| spore | 0.039 | 0.289 | 7.445 | 0.000 | 0.486 | --- | 0.119 | 0.632 | 5.305 | 0.000 | 0.018 | --- | 0.000 | 0.005 | --- |
| pigment | 0.646 | 0.797 | 1.234 | 0.750 | 0.781 | 1.041 | 0.571 | 0.798 | 1.396 | 0.615 | 0.803 | 1.305 | 0.625 | 0.923 | 1.476 |
| nitrate.reduction | 0.306 | 0.290 | 0.948 | 0.378 | 0.341 | 0.901 | 0.208 | 0.222 | 1.068 | 0.370 | 0.339 | 0.918 | 0.083 | 0.163 | 1.952 |
| enzymes | | | | | | | | | | | | | | | |
| acid.phosphatase | 0.050 | 0.231 | 4.615 | 0.081 | 0.183 | 2.251 | 0.075 | 0.103 | 1.365 | 0.014 | 0.232 | 16.910 | 0.083 | 0.417 | 5.005 |
| alkaline.phosphatase | 0.178 | 0.276 | 1.550 | 0.162 | 0.184 | 1.132 | 0.226 | 0.106 | 0.468 | 0.096 | 0.295 | 3.076 | 0.500 | 0.549 | 1.098 |
| aesculin.hydrolysis | 0.294 | 0.308 | 1.044 | 0.270 | 0.328 | 1.213 | 0.396 | 0.333 | 0.841 | 0.205 | 0.233 | 1.134 | 0.583 | 0.462 | 0.792 |
| alpha.galactosidase | 0.067 | 0.081 | 1.209 | 0.054 | 0.074 | 1.375 | 0.094 | 0.060 | 0.637 | 0.000 | 0.042 | --- | 0.417 | 0.196 | 0.471 |
| arylsulfatase | 0.006 | 0.004 | 0.727 | 0.000 | 0.017 | --- | 0.000 | 0.001 | --- | 0.014 | 0.000 | 0.000 | --- | --- | --- |
| catalase | 0.511 | 0.622 | 1.217 | 0.757 | 0.577 | 0.763 | 0.226 | 0.468 | 2.066 | 0.603 | 0.692 | 1.147 | 0.417 | 0.733 | 1.758 |
| oxidase | 0.244 | 0.460 | 1.882 | 0.054 | 0.133 | 2.467 | 0.019 | 0.295 | 15.619 | 0.521 | 0.653 | 1.255 | 0.083 | 0.689 | 8.271 |
| urease | 0.189 | 0.147 | 0.778 | 0.324 | 0.195 | 0.600 | 0.170 | 0.069 | 0.404 | 0.178 | 0.182 | 1.025 | 0.000 | 0.095 | --- |
| gelatinase | 0.228 | 0.197 | 0.866 | 0.297 | 0.221 | 0.743 | 0.151 | 0.157 | 1.043 | 0.233 | 0.142 | 0.610 | 0.417 | 0.361 | 0.866 |
| pyrazinamidase | 0.050 | 0.016 | 0.323 | 0.243 | 0.055 | 0.225 | 0.000 | 0.003 | --- | 0.000 | 0.001 | --- | --- | --- | --- |
| tellurite.reductase | 0.011 | 0.003 | 0.249 | 0.027 | 0.008 | 0.283 | 0.019 | 0.001 | 0.076 | --- | --- | --- | --- | --- | --- |
| gas production | | | | | | | | | | | | | | | |
| H2S.production | 0.056 | 0.055 | 0.984 | 0.054 | 0.062 | 1.152 | 0.038 | 0.063 | 1.668 | 0.082 | 0.053 | 0.639 | 0.000 | 0.037 | --- |
| indole.production | 0.072 | 0.038 | 0.527 | 0.000 | 0.010 | --- | 0.000 | 0.044 | --- | 0.123 | 0.038 | 0.306 | 0.333 | 0.071 | 0.213 |
| methane.production | 0.006 | 0.001 | 0.230 | --- | --- | --- | 0.000 | 0.001 | --- | 0.014 | 0.003 | 0.246 | --- | --- | --- |

**Table S4.3** The proportion of taxa exhibiting a particular binary trait in the world ($\rho_{w}$), among all (>0.001% reads) skin organisms ($\rho_{s}$), and the ratio of the two (${\rho_{w}}/{\rho_{s}}$).

|  | Bacteria | | | Actinobacteria | | | Firmicutes | | | Proteobacteria | | | Bacteroides | | |
| --- | --- | --- | --- | --- | --- | --- | --- | --- | --- | --- | --- | --- | --- | --- | --- |
|  | *ρ_s_* | *ρ_w_* | *ρ_w_/ρ_s_* | *ρ_s_* | *ρ_w_* | *ρ_w_/ρ_s_* | *ρ_s_* | *ρ_w_* | *ρ_w_/ρ_s_* | *ρ_s_* | *ρ_w_* | *ρ_w_/ρ_s_* | *ρ_s_* | *ρ_w_* | *ρ_w_/ρ_s_* |
| general | | | | | | | | | | | | | | | |
| spore | 0.204 | 0.289 | 1.416 | 0.302 | 0.486 | 1.610 | 0.420 | 0.632 | 1.504 | 0.039 | 0.018 | 0.453 | 0.000 | 0.005 | --- |
| pigment | 0.565 | 0.797 | 1.410 | 0.740 | 0.781 | 1.056 | 0.339 | 0.798 | 2.354 | 0.500 | 0.803 | 1.606 | 0.684 | 0.923 | 1.348 |
| nitrate.reduction | 0.263 | 0.290 | 1.103 | 0.338 | 0.341 | 1.008 | 0.171 | 0.222 | 1.300 | 0.365 | 0.339 | 0.931 | 0.109 | 0.163 | 1.487 |
| enzymes | | | | | | | | | | | | | | | |
| acid.phosphatase | 0.066 | 0.231 | 3.501 | 0.115 | 0.183 | 1.586 | 0.043 | 0.103 | 2.415 | 0.042 | 0.232 | 5.532 | 0.219 | 0.417 | 1.907 |
| alkaline.phosphatase | 0.115 | 0.276 | 2.390 | 0.122 | 0.184 | 1.501 | 0.095 | 0.106 | 1.117 | 0.101 | 0.295 | 2.921 | 0.422 | 0.549 | 1.302 |
| aesculin.hydrolysis | 0.213 | 0.308 | 1.443 | 0.295 | 0.328 | 1.112 | 0.360 | 0.333 | 0.925 | 0.126 | 0.233 | 1.855 | 0.422 | 0.462 | 1.095 |
| alpha.galactosidase | 0.054 | 0.081 | 1.505 | 0.079 | 0.074 | 0.939 | 0.071 | 0.060 | 0.845 | 0.010 | 0.042 | 4.306 | 0.219 | 0.196 | 0.898 |
| arylsulfatase | 0.010 | 0.004 | 0.392 | 0.065 | 0.017 | 0.270 | 0.000 | 0.001 | --- | 0.002 | 0.000 | 0.000 | --- | --- | --- |
| catalase | 0.403 | 0.622 | 1.545 | 0.626 | 0.577 | 0.922 | 0.218 | 0.468 | 2.146 | 0.480 | 0.692 | 1.440 | 0.516 | 0.733 | 1.421 |
| oxidase | 0.241 | 0.460 | 1.909 | 0.072 | 0.133 | 1.853 | 0.062 | 0.295 | 4.783 | 0.409 | 0.653 | 1.598 | 0.422 | 0.689 | 1.634 |
| urease | 0.135 | 0.147 | 1.089 | 0.273 | 0.195 | 0.712 | 0.066 | 0.069 | 1.035 | 0.160 | 0.182 | 1.140 | 0.109 | 0.095 | 0.869 |
| gelatinase | 0.175 | 0.197 | 1.126 | 0.259 | 0.221 | 0.852 | 0.147 | 0.157 | 1.071 | 0.165 | 0.142 | 0.861 | 0.438 | 0.361 | 0.824 |
| pyrazinamidase | 0.022 | 0.016 | 0.747 | 0.151 | 0.055 | 0.362 | 0.000 | 0.003 | --- | 0.000 | 0.001 | --- | --- | --- | --- |
| tellurite.reductase | 0.007 | 0.003 | 0.384 | 0.036 | 0.008 | 0.213 | 0.009 | 0.001 | 0.151 | --- | --- | --- | --- | --- | --- |
| gas production | | | | | | | | | | | | | | | |
| H2S.production | 0.100 | 0.055 | 0.547 | 0.065 | 0.062 | 0.962 | 0.081 | 0.063 | 0.781 | 0.138 | 0.053 | 0.381 | 0.063 | 0.037 | 0.593 |
| indole.production | 0.058 | 0.038 | 0.660 | 0.000 | 0.010 | --- | 0.033 | 0.044 | 1.337 | 0.081 | 0.038 | 0.464 | 0.188 | 0.071 | 0.378 |
| methane.production | 0.004 | 0.001 | 0.310 | --- | --- | --- | 0.005 | 0.001 | 0.302 | 0.007 | 0.003 | 0.456 | --- | --- | --- |

As in the main text, we again see that, even within phyla, many traits are differentially observed amongst skin microbes. Some of these traits are unique to a particular phylum. For example, *Actinobacteria* appear to have an over-abundance of representatives that are positive for pyrazinamidase; however this is not observed amongst *Firmicutes, Proteobacteria* or *Bacteroidetes*. Other traits appear to exhibit more consistent differential representation across phyla. Spore formation, for example, is under-represented in skin microbiomes for *Actinobacteria*, *Firmicutes* and abundant *Proteobacteria*. Meanwhile, acid phosphatase is under-represented for all phyla except *Actinobacteria* and abundant *Firmicutes*. Very few trends exhibit changes across phyla (but see H_2_S and indole production and catalase), hinting that differential trait representation depends more on environmental selection than on niche partitioning amongst microbes.

**Figure S4.3** Plots of trait proportions among skin bacteria (pink) and world bacteria (green) for abundant skin taxa (>0.1% of reads in at least one sample). Open red circles denote traits that are overrepresented on skin; filled green circles denote traits that are overrepresented in the world (underrepresented on skin).

**Figure S4.4** Plots of trait proportions among skin bacteria (pink) and world bacteria (green) for abundant skin taxa (>0.001% of reads in at least one sample). Open red circles denote traits that are overrepresented on skin; filled green circles denote traits that are overrepresented in the world (underrepresented on skin).

In Figures S4.3 and S4.4, we show a comparison of categorical traits amongst skin and world *Actinobacteria*, *Firmicutes*, *Proteobacteria*, and *Bacteroidetes* for abundant skin taxa (>0.1% of reads in at least one sample) and all skin taxa (>0.001% of reads in at least one sample) respectively. Tables S4.4, S4.5 and S4.6 summarize these results, along with the results in Figure S3.2 and Figure 3 and Table 3 from the main text.

**Table S4.4** Significant differences between skin microbes and microbes from the world for categorical traits. ‘W’ indicates that a trait that is signficantly overrepresented among world microbes; ‘S’ indicates that it is significantly overrepresented among skin microbes; ‘n/a’ indicates that a trait was not reported from a particular environment. Outcomes for abundant skin taxa (>0.1% of reads in at least one sample) are stated first (black); outcomes for the full skin community (>0.001% of reads in at least one sample) are stated second (grey). One star indicates that $0.67<{\rho_{w}}/{\rho_{s}}<1.5$ and $\rho_{w}>0.05$, where $\rho_{w}$ and $\rho_{s}$ are the proportions of taxa exhibiting a particular trait in the world and on skin respectively. Two stars indicate that $0.5<{\rho_{w}}/{\rho_{s}}<2$ and $\rho_{w}>0.05$.

|  | **Bacteria** | | **Actinobacteria** | | **Firmicutes** | | **Proteobacteria** | | **Bacteroidetes** | |
| --- | --- | --- | --- | --- | --- | --- | --- | --- | --- | --- |
| oxygen use | | | | | | | | | | |
| aerobic | W* | W** | W* | W* | W** | W** | W | W* | W** | W* |
| anaerobic |  | S** |  | S |  | S* |  |  | S** | S** |
| facultative | S** | S** | S** | S** | S** | S* | S* | S* |  |  |
| microaerophilic |  | S |  | S | W |  |  | S |  |  |
| motility | | | | | | | | | | |
| non-motile |  | W |  |  | S* |  | W* | W* |  |  |
| axial filament | W |  |  |  | n/a | |  | | n/a | |
| flagella |  | S |  |  | W** |  | S | S |  |  |
| gliding | W |  |  |  |  |  | W |  |  |  |
| type IV pili |  | S | n/a | | n/a | | S | | n/a | |
| shape | | | | | | | | | | |
| cocci | S** | S* |  |  | S** |  |  |  | W | W |
| ovoid/coccobacillus | S** | S |  |  | S | S |  |  |  |  |
| rod | W | W |  |  | W** | W |  |  |  |  |
| spirillum | W | S | W |  | n/a | | W | |  |  |
| gram | | | | | | | | | | |
| Gram negative |  | S |  |  |  | S** | S |  | S | S |
| Gram positive |  | W |  | W | S |  | W |  | W | W |
| Gram variable |  |  |  |  | W** W** | |  |  | n/a | |
| aggregation | | | | | | | | | | |
| chain | W* | W** | W** | W* |  | W* | W** | W** | W | W** |
| clump | S* | S* | S** | S** |  | S** | S* | S* |  |  |
| single |  | S* |  |  |  |  |  | S** |  | S |

**Table S4.5** The proportion of taxa exhibiting a particular qualitative trait in the world ($\rho_{w}$), among abundant (>0.1% reads) skin organisms ($\rho_{s}$), and the ratio of the two (${\rho_{w}}/{\rho_{s}}$).

|  | Bacteria | | | Actinobacteria | | | Firmicutes | | | Proteobacteria | | | Bacteroides | | |
| --- | --- | --- | --- | --- | --- | --- | --- | --- | --- | --- | --- | --- | --- | --- | --- |
|  | *ρ_s_* | *ρ_w_* | *ρ_w_/ρ_s_* | *ρ_s_* | *ρ_w_* | *ρ_w_/ρ_s_* | *ρ_s_* | *ρ_w_* | *ρ_w_/ρ_s_* | *ρ_s_* | *ρ_w_* | *ρ_w_/ρ_s_* | *ρ_s_* | *ρ_w_* | *ρ_w_/ρ_s_* |
| oxygen use | | | | | | | | | | | | | | | |
| aerobic | 0.360 | 0.710 | 1.971 | 0.559 | 0.904 | 1.618 | 0.019 | 0.420 | 21.862 | 0.549 | 0.718 | 1.307 | 0.083 | 0.826 | 9.914 |
| anaerobic | 0.151 | 0.122 | 0.808 | 0.088 | 0.032 | 0.363 | 0.192 | 0.278 | 1.444 | 0.042 | 0.087 | 2.059 | 0.833 | 0.103 | 0.124 |
| facultative | 0.453 | 0.157 | 0.346 | 0.294 | 0.055 | 0.187 | 0.788 | 0.293 | 0.371 | 0.352 | 0.182 | 0.516 | 0.083 | 0.071 | 0.847 |
| microaerophilic | 0.035 | 0.011 | 0.307 | 0.059 | 0.009 | 0.152 | 0.000 | 0.009 | --- | 0.056 | 0.014 | 0.242 | --- | --- | --- |
| motility | | | | | | | | | | | | | | | |
| non-motile | 0.648 | 0.642 | 0.989 | 0.943 | 0.952 | 1.010 | 0.894 | 0.579 | 0.648 | 0.235 | 0.431 | 1.830 | 0.917 | 0.762 | 0.831 |
| axial filament | 0.000 | 0.002 | --- | 0.000 | 0.003 | --- | --- | --- | --- | 0.000 | 0.001 | --- | --- | --- | --- |
| flagella | 0.333 | 0.308 | 0.925 | 0.057 | 0.040 | 0.699 | 0.106 | 0.419 | 3.936 | 0.735 | 0.564 | 0.767 | 0.000 | 0.005 | --- |
| gliding | 0.006 | 0.048 | 7.955 | 0.000 | 0.005 | --- | 0.000 | 0.002 | --- | 0.000 | 0.005 | --- | 0.083 | 0.233 | 2.798 |
| type IV pili | 0.012 | 0.000 | 0.000 | --- | --- | --- | --- | --- | --- | 0.029 | 0.000 | 0.000 | --- | --- | --- |
| shape | | | | | | | | | | | | | | | |
| cocci | 0.200 | 0.085 | 0.424 | 0.226 | 0.150 | 0.665 | 0.423 | 0.159 | 0.375 | 0.048 | 0.040 | 0.831 | 0.000 | 0.013 | --- |
| ovoid/coccobacillus | 0.144 | 0.066 | 0.462 | 0.032 | 0.098 | 3.040 | 0.231 | 0.037 | 0.161 | 0.095 | 0.072 | 0.758 | 0.167 | 0.020 | 0.119 |
| rod | 0.656 | 0.837 | 1.276 | 0.742 | 0.740 | 0.997 | 0.346 | 0.804 | 2.323 | 0.857 | 0.873 | 1.018 | 0.833 | 0.964 | 1.156 |
| spirillum | 0.000 | 0.012 | --- | 0.000 | 0.012 | --- | --- | --- | --- | 0.000 | 0.015 | --- | 0.000 | 0.003 | --- |
| gram | | | | | | | | | | | | | | | |
| Gram negative | 0.533 | 0.611 | 1.145 | 0.000 | 0.004 | --- | 0.068 | 0.114 | 1.679 | 1.000 | 0.996 | 0.996 | 1.000 | 0.994 | 0.994 |
| Gram positive | 0.461 | 0.374 | 0.812 | 0.971 | 0.992 | 1.022 | 0.932 | 0.813 | 0.873 | 0.000 | 0.003 | --- | 0.000 | 0.006 | --- |
| Gram variable | 0.006 | 0.015 | 2.524 | 0.029 | 0.005 | 0.162 | 0.000 | 0.072 | --- | 0.000 | 0.001 | --- | --- | --- | --- |
| aggregation | | | | | | | | | | | | | | | |
| chain | 0.300 | 0.571 | 1.904 | 0.077 | 0.672 | 8.739 | 0.652 | 0.618 | 0.947 | 0.111 | 0.548 | 4.936 | 0.000 | 0.526 | --- |
| clump | 0.550 | 0.330 | 0.600 | 0.769 | 0.258 | 0.336 | 0.304 | 0.261 | 0.859 | 0.667 | 0.365 | 0.548 | 0.500 | 0.427 | 0.854 |
| single | 0.150 | 0.098 | 0.657 | 0.154 | 0.069 | 0.451 | 0.043 | 0.121 | 2.776 | 0.222 | 0.087 | 0.389 | 0.500 | 0.047 | 0.095 |

**Table S4.6** The proportion of taxa exhibiting a particular qualitative trait in the world ($\rho_{w}$), among all (>0.001% reads) skin organisms ($\rho_{s}$), and the ratio of the two (${\rho_{w}}/{\rho_{s}}$).

|  | Bacteria | | | Actinobacteria | | | Firmicutes | | | Proteobacteria | | | Bacteroides | | |
| --- | --- | --- | --- | --- | --- | --- | --- | --- | --- | --- | --- | --- | --- | --- | --- |
|  | *ρ_s_* | *ρ_w_* | *ρ_w_/ρ_s_* | *ρ_s_* | *ρ_w_* | *ρ_w_/ρ_s_* | *ρ_s_* | *ρ_w_* | *ρ_w_/ρ_s_* | *ρ_s_* | *ρ_w_* | *ρ_w_/ρ_s_* | *ρ_s_* | *ρ_w_* | *ρ_w_/ρ_s_* |
| oxygen use | | | | | | | | | | | | | | | |
| aerobic | 0.350 | 0.710 | 2.031 | 0.567 | 0.904 | 1.595 | 0.090 | 0.420 | 4.671 | 0.425 | 0.718 | 1.688 | 0.472 | 0.826 | 1.751 |
| anaerobic | 0.251 | 0.122 | 0.486 | 0.126 | 0.032 | 0.254 | 0.440 | 0.278 | 0.631 | 0.115 | 0.087 | 0.753 | 0.377 | 0.103 | 0.274 |
| facultative | 0.319 | 0.157 | 0.492 | 0.220 | 0.055 | 0.250 | 0.455 | 0.293 | 0.644 | 0.335 | 0.182 | 0.542 | 0.094 | 0.071 | 0.748 |
| microaerophilic | 0.081 | 0.011 | 0.133 | 0.087 | 0.009 | 0.103 | 0.015 | 0.009 | 0.601 | 0.124 | 0.014 | 0.110 | 0.057 | 0.000 | 0.000 |
| motility | | | | | | | | | | | | | | | |
| non-motile | 0.518 | 0.642 | 1.239 | 0.924 | 0.952 | 1.031 | 0.598 | 0.579 | 0.969 | 0.250 | 0.431 | 1.722 | 0.623 | 0.762 | 1.223 |
| axial filament | 0.001 | 0.002 | 1.606 | 0.000 | 0.003 | --- | --- | --- | --- | 0.003 | 0.001 | 0.307 | --- | --- | --- |
| flagella | 0.431 | 0.308 | 0.715 | 0.076 | 0.040 | 0.524 | 0.402 | 0.419 | 1.041 | 0.726 | 0.564 | 0.777 | 0.016 | 0.005 | 0.316 |
| gliding | 0.042 | 0.048 | 1.147 | 0.000 | 0.005 | --- | 0.000 | 0.002 | --- | 0.009 | 0.005 | 0.512 | 0.361 | 0.233 | 0.646 |
| type IV pili | 0.007 | 0.000 | 0.000 | --- | --- | --- | --- | --- | --- | 0.012 | 0.000 | 0.000 | --- | --- | --- |
| shape | | | | | | | | | | | | | | | |
| cocci | 0.137 | 0.085 | 0.619 | 0.180 | 0.150 | 0.833 | 0.220 | 0.159 | 0.722 | 0.042 | 0.040 | 0.950 | 0.000 | 0.013 | --- |
| ovoid/coccobacillus | 0.095 | 0.066 | 0.696 | 0.045 | 0.098 | 2.177 | 0.120 | 0.037 | 0.309 | 0.089 | 0.072 | 0.809 | 0.049 | 0.020 | 0.407 |
| rod | 0.741 | 0.837 | 1.129 | 0.766 | 0.740 | 0.966 | 0.660 | 0.804 | 1.218 | 0.854 | 0.873 | 1.022 | 0.902 | 0.964 | 1.068 |
| spirillum | 0.026 | 0.012 | 0.438 | 0.009 | 0.012 | 1.319 | --- | --- | --- | 0.015 | 0.015 | 1.027 | 0.049 | 0.003 | 0.068 |
| gram | | | | | | | | | | | | | | | |
| Gram negative | 0.687 | 0.611 | 0.889 | 0.016 | 0.004 | 0.220 | 0.254 | 0.114 | 0.450 | 0.995 | 0.996 | 1.001 | 1.000 | 0.994 | 0.994 |
| Gram positive | 0.301 | 0.374 | 1.242 | 0.951 | 0.992 | 1.043 | 0.734 | 0.813 | 1.108 | 0.003 | 0.003 | 1.320 | 0.000 | 0.006 | --- |
| Gram variable | 0.012 | 0.015 | 1.285 | 0.033 | 0.005 | 0.146 | 0.012 | 0.072 | 6.268 | 0.003 | 0.001 | 0.264 | --- | --- | --- |
| aggregation | | | | | | | | | | | | | | | |
| chain | 0.267 | 0.571 | 2.139 | 0.341 | 0.672 | 1.969 | 0.379 | 0.618 | 1.630 | 0.190 | 0.548 | 2.879 | 0.190 | 0.526 | 2.759 |
| clump | 0.577 | 0.330 | 0.573 | 0.585 | 0.258 | 0.441 | 0.526 | 0.261 | 0.497 | 0.629 | 0.365 | 0.581 | 0.476 | 0.427 | 0.897 |
| single | 0.156 | 0.098 | 0.630 | 0.073 | 0.069 | 0.949 | 0.095 | 0.121 | 1.274 | 0.181 | 0.087 | 0.478 | 0.333 | 0.047 | 0.142 |

Again, we find that traits are over-/under-represented even within phyla, suggesting that trait selection is not occurring primarily via broad preference for specific bacterial linneages. Some traits, for example oxygen use and aggregation, show striking patterns of conservation across phyla. Other traits, for example shape, show more variability. The latter might indicate niche partitioning, whereas the former hints at forces in the environment governing which microbes can/cannot colonize the skin surface.

**Table S4.7** Significant differences between skin microbes and microbes from the world. ‘L’ indicates that a trait is signficantly lower among skin taxa; ‘H’ indicates that it is significantly higher. Outcomes for abundant skin taxa (>0.1% of reads in at least one sample) are stated first (black); outcomes for the full skin community (>0.001% of reads in at least one sample) are stated second (grey). One star indicates that $\left| \frac{v_{w}-v_{s}}{v_{w}} \right|>0.25$, where $v_{w}$ and $v_{s}$ are the mean values of the trait in the world and on skin respectively. Two stars indicate that $\left| \frac{v_{w}-v_{s}}{v_{w}} \right|>1$.

|  | **Bacteria** | | **Actinobacteria** | | **Firmicutes** | | **Proteobacteria** | | **Bacteroidetes** | |
| --- | --- | --- | --- | --- | --- | --- | --- | --- | --- | --- |
| GC content | L | L | L | L | L | L |  | L |  |  |
| pH | | | | | | | | | | |
| pH optimum |  | L |  | L |  |  |  |  |  |  |
| pH lower bound |  |  |  |  |  |  |  |  |  |  |
| pH upper bound | L | L | L |  | L | L |  | L |  |  |
| pH range | L* | L | L* | L |  | L* | L | L |  |  |
| temperature | | | | | | | | | | |
| temp. optimum | H | H | H | H | H | | H | H |  |  |
| temp. lower bound | H* | H* |  | H |  | H |  | H | H** | H* |
| temp. upper bound | H | H | H | H |  | H |  |  | H |  |
| temp. range |  | L |  |  |  |  |  | L | L |  |
| NaCl | | | | | | | | | | |
| NaCl optimum |  |  | H** | H** |  |  |  |  |  |  |
| NaCl lower bound | H** | H** |  |  | H** H** | |  |  |  |  |
| NaCl upper bound |  |  | H* |  |  | |  |  |  | |
| NaCl range |  |  |  |  |  |  |  |  | L** |  |

**Table S4.8** Mean values of quantitative traits across world organisms ($v_{w}$), abundant (>0.1% reads) skin organisms ($v_{s}$), and the relative difference between the two $\boldsymbol{\Delta}v=\left| \frac{v_{w}-v_{s}}{v_{w}} \right|$.

|  | Bacteria | | | Actinobacteria | | | Firmicutes | | | Proteobacteria | | | Bacteroides | | |
| --- | --- | --- | --- | --- | --- | --- | --- | --- | --- | --- | --- | --- | --- | --- | --- |
|  | *v_s_* | *v_w_* | Δ*v* | *v_s_* | *v_w_* | Δ*v* | *v_s_* | *v_w_* | Δ*v* | *v_s_* | *v_w_* | Δ*v* | *v_s_* | *v_w_* | Δ*v* |
| GC content | 51.74 | 54.79 | 0.06 | 62.28 | 69.35 | 0.10 | 37.42 | 43.22 | 0.13 | 57.82 | 58.74 | 0.02 | 44.48 | 41.02 | -0.08 |
| pH | | | | | | | | | | | | | | | |
| pH optimum | 7.06 | 7.22 | 0.02 | 6.93 | 7.23 | 0.04 | 6.76 | 7.30 | 0.07 | 7.11 | 7.22 | 0.01 | 7.93 | 7.22 | -0.10 |
| pH lower bound | 5.72 | 5.65 | -0.01 | 6.00 | 5.56 | -0.08 | 5.19 | 5.78 | 0.10 | 5.94 | 5.65 | -0.05 | 6.50 | 5.72 | -0.14 |
| pH upper bound | 7.97 | 9.03 | 0.12 | 8.43 | 9.39 | 0.10 | 6.95 | 9.02 | 0.23 | 8.32 | 8.94 | 0.07 | --- | --- | --- |
| pH range | 2.41 | 3.38 | 0.29 | 2.10 | 3.95 | 0.47 | 2.28 | 3.16 | 0.28 | 2.59 | 3.32 | 0.22 | --- | --- | --- |
| temperature | | | | | | | | | | | | | | | |
| temp. optimum | 33.23 | 30.82 | -0.08 | 32.30 | 29.41 | -0.10 | 35.60 | 36.44 | 0.02 | 31.25 | 29.42 | -0.06 | 34.80 | 28.02 | -0.24 |
| temp. lower bound | 18.84 | 15.07 | -0.25 | 17.12 | 16.09 | -0.06 | 18.20 | 19.86 | 0.08 | 17.42 | 13.52 | -0.29 | 25.83 | 10.44 | -1.47 |
| temp. upper bound | 42.29 | 39.49 | -0.07 | 39.85 | 37.84 | -0.05 | 46.26 | 46.40 | 0.00 | 39.50 | 37.98 | -0.04 | 44.00 | 35.90 | -0.23 |
| temp. range | 25.26 | 24.72 | -0.02 | 22.76 | 21.98 | -0.04 | 28.94 | 26.75 | -0.08 | 24.95 | 24.81 | -0.01 | 20.20 | 25.87 | 0.22 |
| NaCl | | | | | | | | | | | | | | | |
| NaCl optimum | 1.23 | 0.77 | -0.59 | 2.07 | 0.84 | -1.45 | 1.71 | 1.10 | -0.56 | 0.29 | 0.72 | 0.59 | --- | --- | --- |
| NaCl lower bound | 1.09 | 0.02 | -64.36 | 0.62 | 0.68 | 0.10 | 1.94 | 0.44 | -3.45 | -0.25 | -0.16 | -0.59 | -0.80 | -0.16 | -3.97 |
| NaCl upper bound | 1.68 | 1.55 | -0.09 | 2.27 | 1.61 | -0.42 | 2.25 | 1.91 | -0.18 | 1.21 | 1.51 | 0.20 | 0.37 | 1.26 | 0.70 |
| NaCl range | 1.14 | 1.49 | 0.23 | 2.53 | 1.54 | -0.64 | 1.70 | 1.81 | 0.06 | 0.74 | 1.45 | 0.49 | -1.41 | 1.21 | 2.17 |

**Table S4.9** Mean values of quantitative traits across world organisms ($v_{w}$), abundant (>0.1% reads) skin organisms ($v_{s}$), and the relative difference between the two $\boldsymbol{\Delta}v=\left| \frac{v_{w}-v_{s}}{v_{w}} \right|$.

|  | Bacteria | | | Actinobacteria | | | Firmicutes | | | Proteobacteria | | | Bacteroides | | |
| --- | --- | --- | --- | --- | --- | --- | --- | --- | --- | --- | --- | --- | --- | --- | --- |
|  | *v_s_* | *v_w_* | Δ*v* | *v_s_* | *v_w_* | Δ*v* | *v_s_* | *v_w_* | Δ*v* | *v_s_* | *v_w_* | Δ*v* | *v_s_* | *v_w_* | Δ*v* |
| GC content | 50.58 | 54.79 | 0.08 | 65.00 | 69.35 | 0.06 | 40.35 | 43.22 | 0.07 | 54.52 | 58.74 | 0.07 | 42.33 | 41.02 | -0.03 |
| pH | | | | | | | | | | | | | | | |
| pH optimum | 7.04 | 7.22 | 0.03 | 6.85 | 7.23 | 0.05 | 7.15 | 7.30 | 0.02 | 7.09 | 7.22 | 0.02 | 7.37 | 7.22 | -0.02 |
| pH lower bound | 5.60 | 5.65 | 0.01 | 5.84 | 5.56 | -0.05 | 5.61 | 5.78 | 0.03 | 5.52 | 5.65 | 0.02 | 5.57 | 5.72 | 0.03 |
| pH upper bound | 8.30 | 9.03 | 0.08 | 9.04 | 9.39 | 0.04 | 7.98 | 9.02 | 0.12 | 8.55 | 8.94 | 0.04 | 8.31 | 8.96 | 0.07 |
| pH range | 2.74 | 3.38 | 0.19 | 3.22 | 3.95 | 0.18 | 2.34 | 3.16 | 0.26 | 3.03 | 3.32 | 0.09 | 3.24 | 3.12 | -0.04 |
| temperature | | | | | | | | | | | | | | | |
| temp. optimum | 34.96 | 30.82 | -0.13 | 33.14 | 29.41 | -0.13 | 40.57 | 36.44 | -0.11 | 30.66 | 29.42 | -0.04 | 28.63 | 28.02 | -0.02 |
| temp. lower bound | 20.27 | 15.07 | -0.34 | 19.45 | 16.09 | -0.21 | 22.95 | 19.86 | -0.16 | 16.55 | 13.52 | -0.22 | 15.66 | 10.44 | -0.50 |
| temp. upper bound | 42.70 | 39.49 | -0.08 | 40.95 | 37.84 | -0.08 | 49.81 | 46.40 | -0.07 | 37.57 | 37.98 | 0.01 | 38.51 | 35.90 | -0.07 |
| temp. range | 23.53 | 24.72 | 0.05 | 21.82 | 21.98 | 0.01 | 27.46 | 26.75 | -0.03 | 22.51 | 24.81 | 0.09 | 24.83 | 25.87 | 0.04 |
| NaCl | | | | | | | | | | | | | | | |
| NaCl optimum | 0.89 | 0.77 | -0.15 | 2.04 | 0.84 | -1.43 | 1.08 | 1.10 | 0.02 | 0.83 | 0.72 | -0.16 | 1.09 | 0.52 | -1.12 |
| NaCl lower bound | 0.35 | 0.02 | -19.87 | 0.50 | 0.68 | 0.26 | 1.09 | 0.44 | -1.51 | 0.13 | -0.16 | 1.80 | -0.08 | -0.16 | 0.48 |
| NaCl upper bound | 1.52 | 1.55 | 0.02 | 1.74 | 1.61 | -0.09 | 1.70 | 1.91 | 0.11 | 1.54 | 1.51 | -0.02 | 1.08 | 1.26 | 0.14 |
| NaCl range | 1.40 | 1.49 | 0.06 | 1.47 | 1.54 | 0.04 | 1.47 | 1.81 | 0.19 | 1.55 | 1.45 | -0.07 | 0.97 | 1.21 | 0.20 |

Tables S4.7, S4.8 and S4.9 summarize results for quantitative traits across phyla (see also Figure 4 and Table 4 in the main text). Again, we see conservation across phyla for GC content, pH upper bound and range, and temperature optimum, lower and upper bound and, to some extent, range. By contrast, NaCl requirements are more specific to individual phyla, with overall trends driven by *Firmicutes*.

**Table S4.10** p-values for our phylogenetically corrected and naïve regression models for each binary trait. Traits shown in purple are significantly different between skin and world taxa, regardless of whether phylogeny is accounted for. Traits shown in red are only significantly different when phylogeny is accounted for. Traits shown in black are never significantly different.

| **Property** | **p-value**  **(phylogenetic correction)** | **p-value**  **(naive)** |
| --- | --- | --- |
| pigment | 0 | 0 |
| acid.phosphatase | 0 | 0 |
| alkaline.phosphatase | 0 | 0 |
| aesculin.hydrolysis | 0 | 0 |
| catalase | 0 | 0 |
| oxidase | 0 | 0 |
| H2S.production | 0 | 0.0000001 |
| gram | 0 | 0.0000664 |
| alpha.galactosidase | 0 | 0.0034644 |
| indole.production | 0.0000001 | 0.0046263 |
| arylsulfatase | 0.0223193 | 0.015741 |
| tellurite.reductase | 0.0224166 | 0.0354029 |
| gelatinase | 0.0000079 | 0.0831803 |
| nitrate.reduction | 0.0000002 | 0.0853473 |
| pyrazinamidase | 0.000415 | 0.2793105 |
| urease | 0.0000259 | 0.2929794 |
| methane.production | 0.1911093 | 0.079127 |

**Table S4.11** p-values for our phylogenetically corrected and naïve regression models for each qualitative trait. Traits shown in purple are significantly different between skin and world taxa, regardless of whether phylogeny is accounted for.

| **Property** | **p-value**  **(phylogenetic correction)** | **p-value**  **(naive)** |
| --- | --- | --- |
| oxygen | 0 | 0 |
| specific_motility | 0 | 0 |
| shape | 0 | 0 |
| aggregation | 0 | 0 |
| spore | 0.0000002 | 0.0000095 |

*Supplementary Information V: Trait Comparison Across Skin Environments*

Below, we show figures comparing qualitative and quantitative traits among the three different skin environments. Almost no trends are significant (see main text).


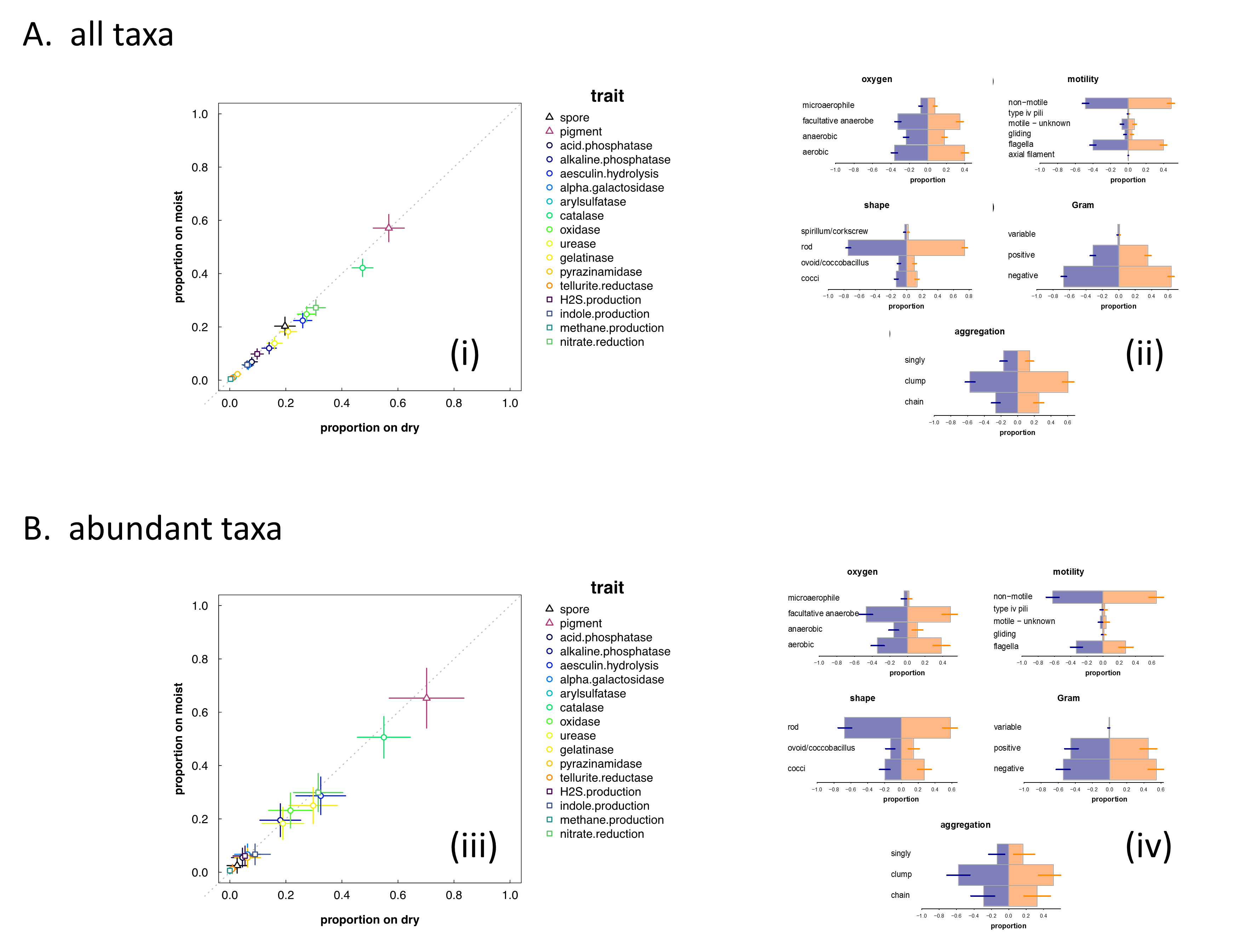


**Figure S5.1** Trait comparisons for (A) all skin taxa (>0.001% of reads in at least one sample) and (B) abundant skin taxa (>0.1% of reads in at least one sample). Panels (i,iii), illustrate the proportion of taxa with a specific, qualitative trait at dry sites (x-axis) versus at moist sites (y-axis). Filled symbols represent traits that are significantly different between the two sites; open circles represent traits that are not. Panels (ii,iv), show plots of trait proportions among moist skin bacteria (blue) and dry skin bacteria (peach). No differences are significant.


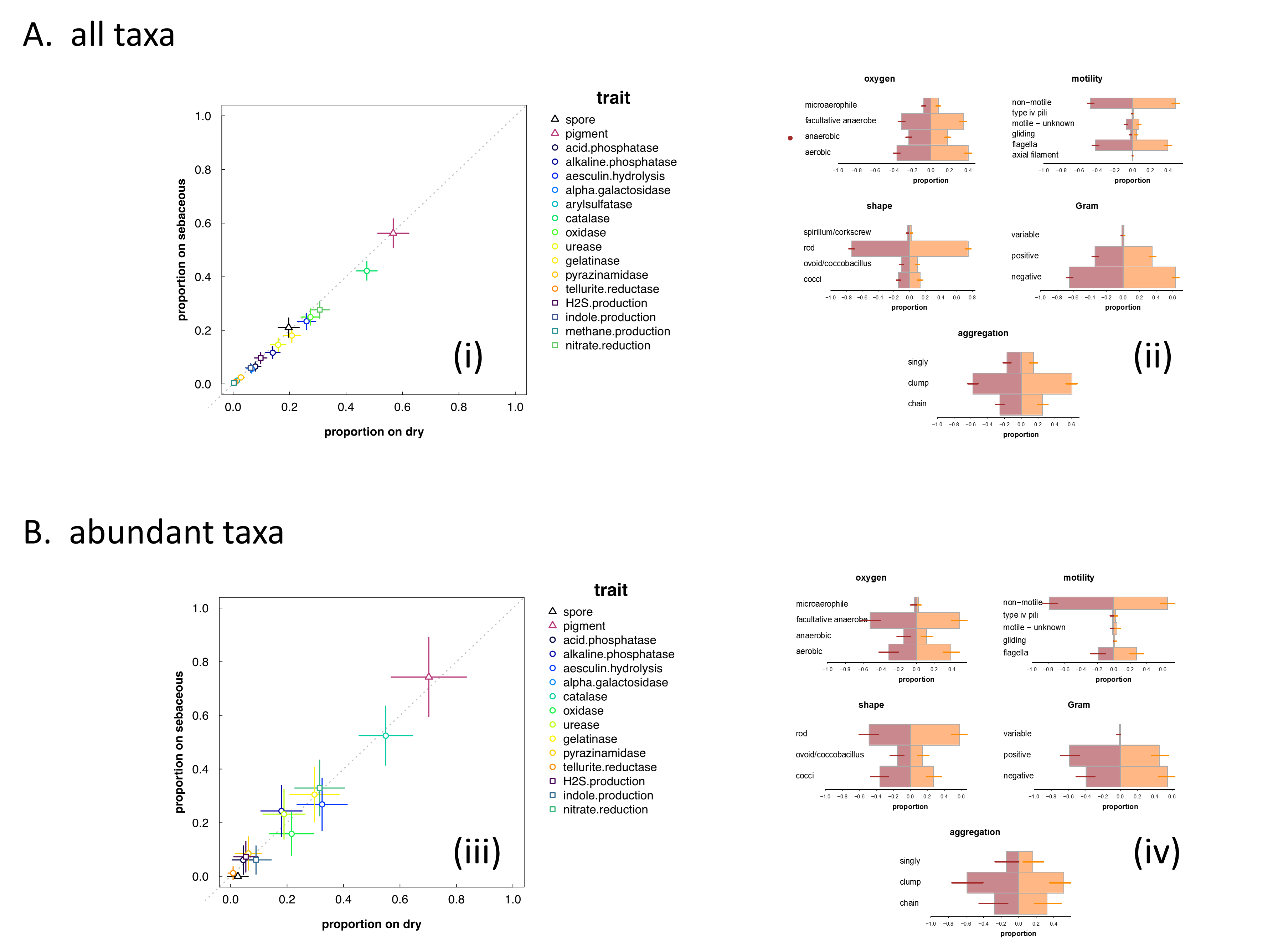


**Figure S5.2** Trait comparisons for (A) all skin taxa (>0.001% of reads in at least one sample) and (B) abundant skin taxa (>0.1% of reads in at least one sample). Panels (i,iii), illustrate the proportion of taxa with a specific, qualitative trait at dry sites (x-axis) versus at sebaceous sites (y-axis). Filled symbols represent traits that are significantly different between the two sites; open circles represent traits that are not; symbol size reflects significance. Panels (ii,iv), show plots of trait proportions among dry skin bacteria (peach) and sebaceous skin bacteria (red). The filled red circle denotes the one trait that is overrepresented on sebaceous skin.


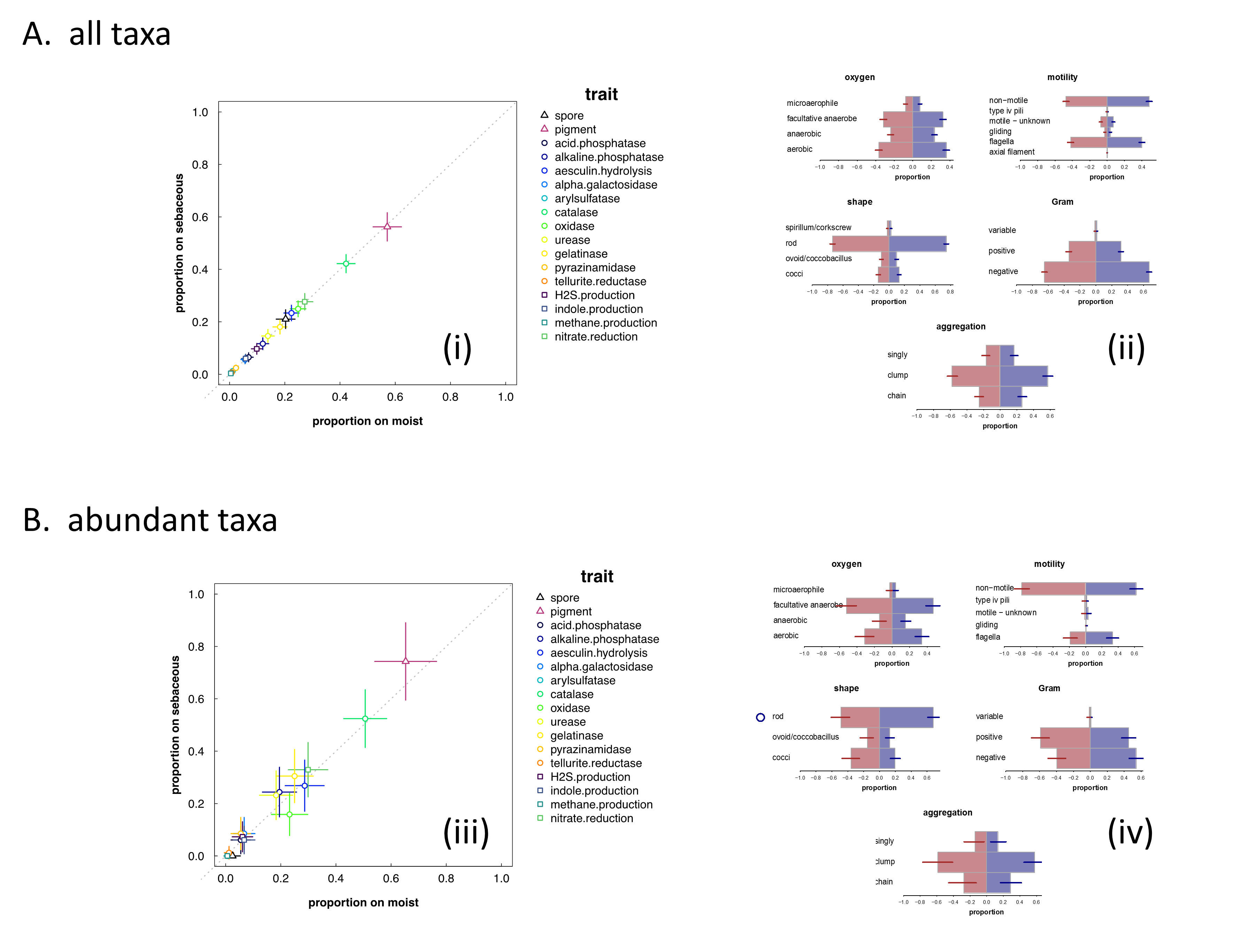


**Figure S5.3** Trait comparisons for (A) all skin taxa (>0.001% of reads in at least one sample) and (B) abundant skin taxa (>0.1% of reads in at least one sample). Panels (i,iii), illustrate the proportion of taxa with a specific, qualitative trait at moist sites (x-axis) versus at sebaceous sites (y-axis). Filled symbols represent traits that are significantly different between the two sites; open circles represent traits that are not; symbol size reflects significance. Panels (ii,iv), show plots of trait proportions among moist skin bacteria (blue) and sebaceous skin bacteria (red). The open blue circle denotes the one trait that was overrepresented on moist skin.


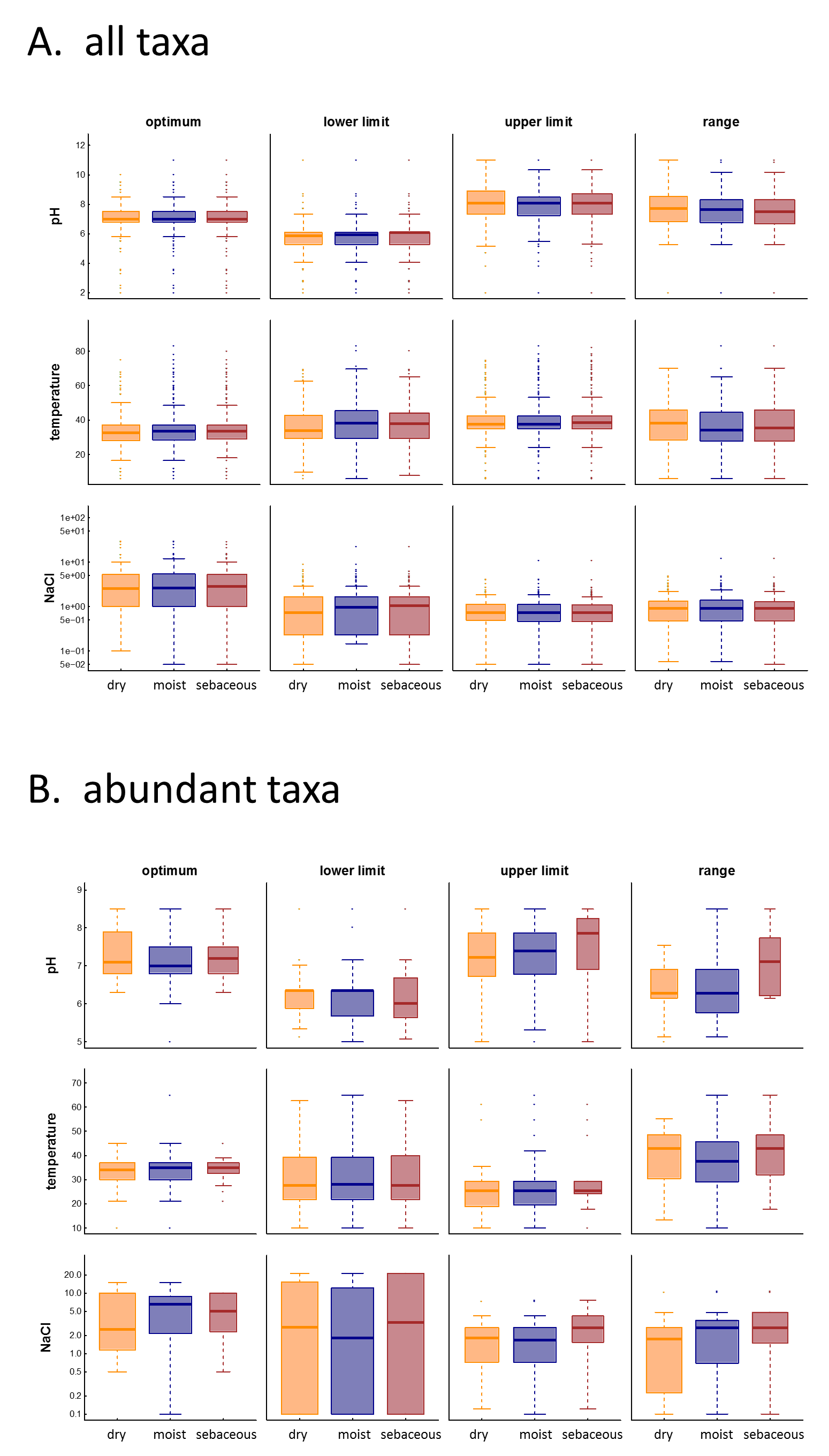


**Figure S5.4** Boxplots comparing quantitative traits among skin bacteria from dry (peach), moist (blue) and sebaceous (red) sites for (A) all skin bacteria (>0.001% of reads in at least one sample) and (B) abundant skin bacteria (>0.1% of reads in at least one sample). No differences are signficant.


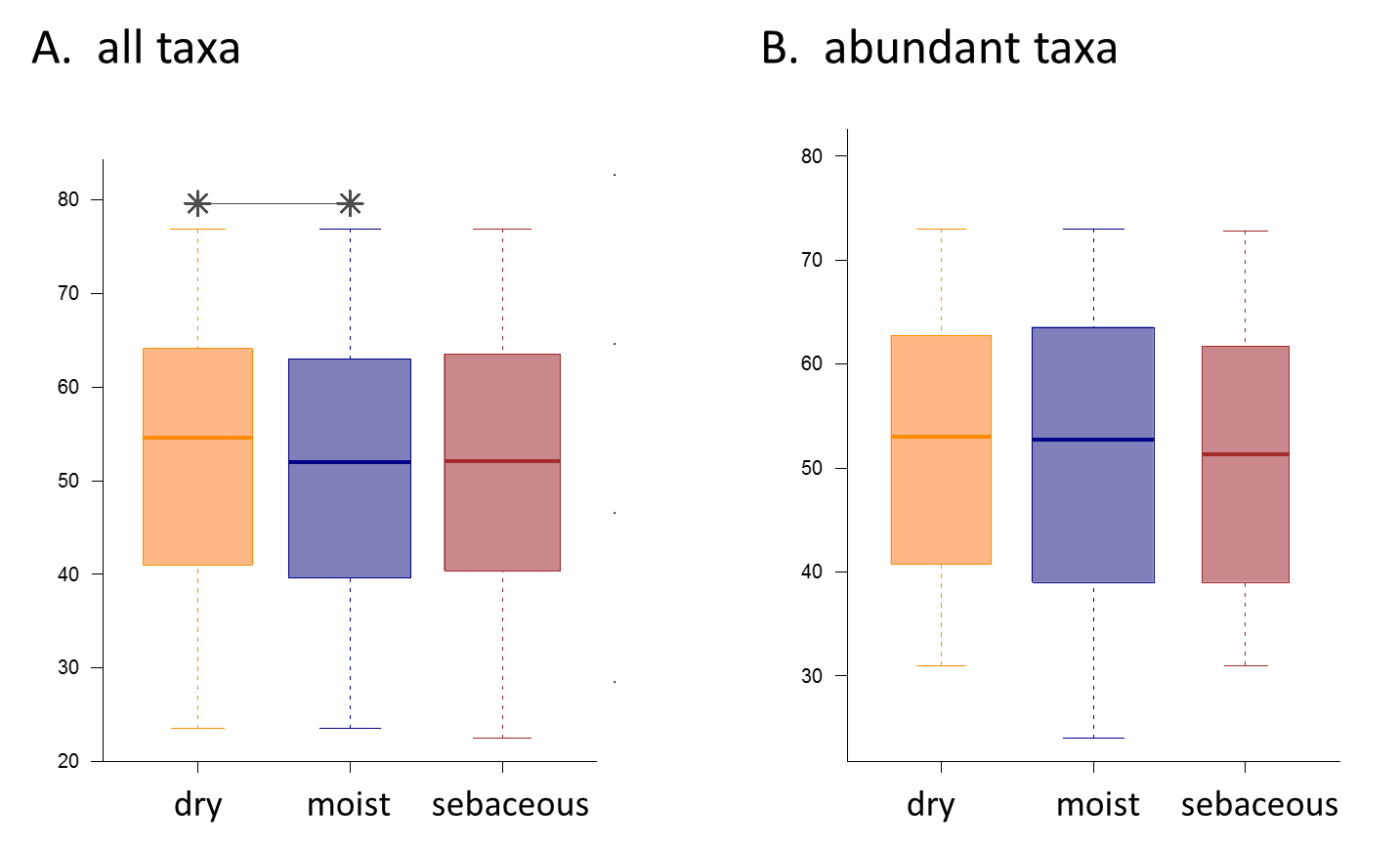


**Figure S5.5** Boxplots comparing GC percent among skin bacteria from dry (peach), moist (blue), and sebaceous (red) skin environments. Stars are used to denote significant differences between a trait value in the world versus on skin. Box width indicates the relative number of microbes used for the comparison. Analyses are shown for (A) all skin bacteria (>0.001% of reads in at least one sample) and (B) abundant skin taxa (>0.1% of reads in at least one sample).

In Figures S4.6 – S4.11, we show comparisons for substrate use across the three different skin environments. Again, there is little difference, although there are a few differences between moist and sebaceous sites. Specifically, bacterial use of three organic acids – quinate, malonate, and caprate – as well as glucosamine (a monosaccharide) is overrepresented at sebaceous sites. By contrast, bacterial use of three saccharides –rhamnose, xylose, and cellobiose – as well as glycine (an amino acid) and urea are overrepresented at moist sites.

*
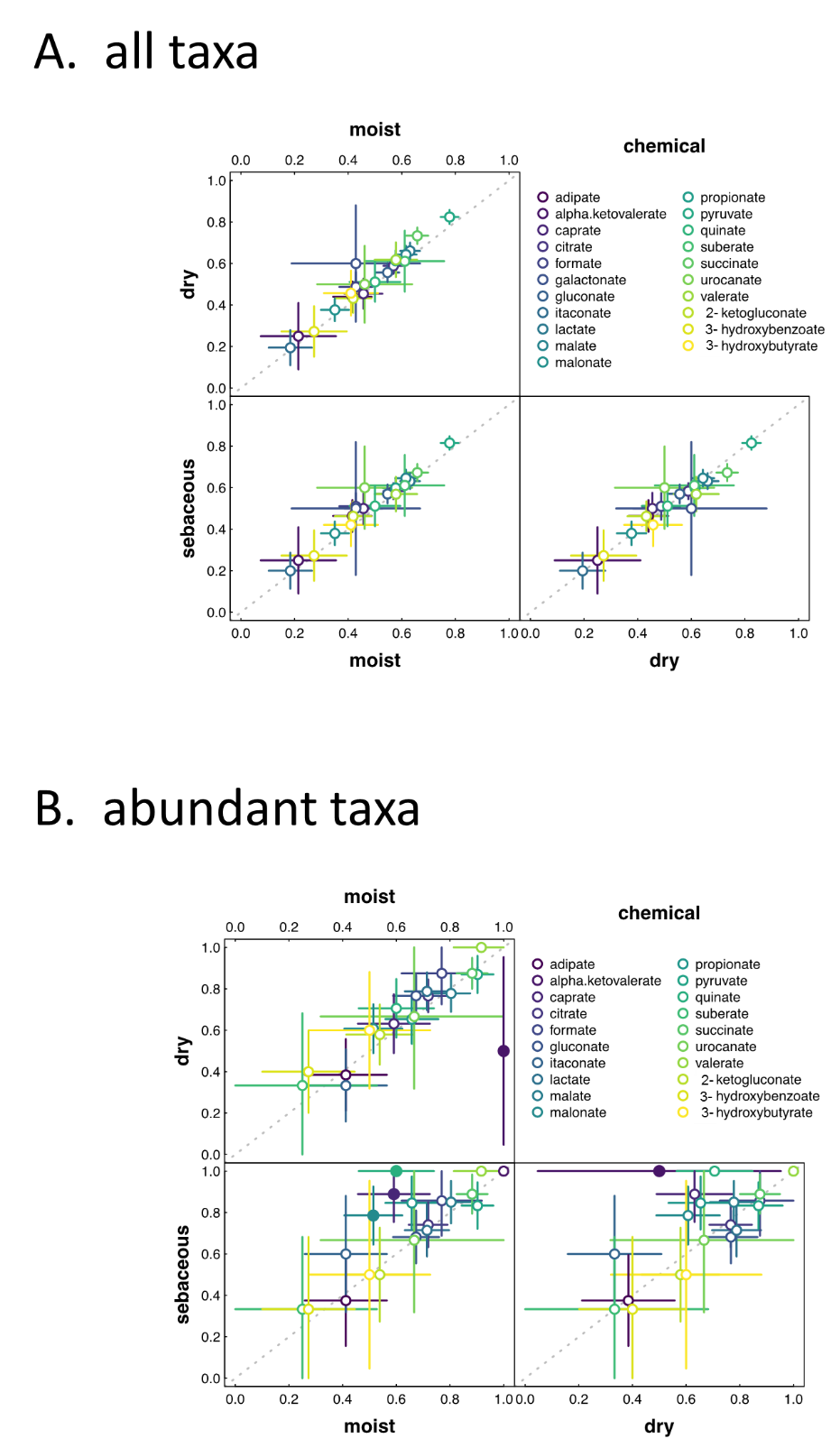
*

**Figure S5.6** Pairwise comparison (dry vs. moist, dry vs. sebaceous, moist vs. sebaceous) of organic acid use. Analyses are shown for (A) all skin bacteria (>0.001% of reads in at least one sample) and (B) abundant skin taxa (>0.1% of reads in at least one sample). Filled circles indicate a significant difference between sites; open circles indicate the opposite. Note that in the main text, we do not assume that results for α-ketovalerate are signficant, because there were too few taxa for which this trait was reported.


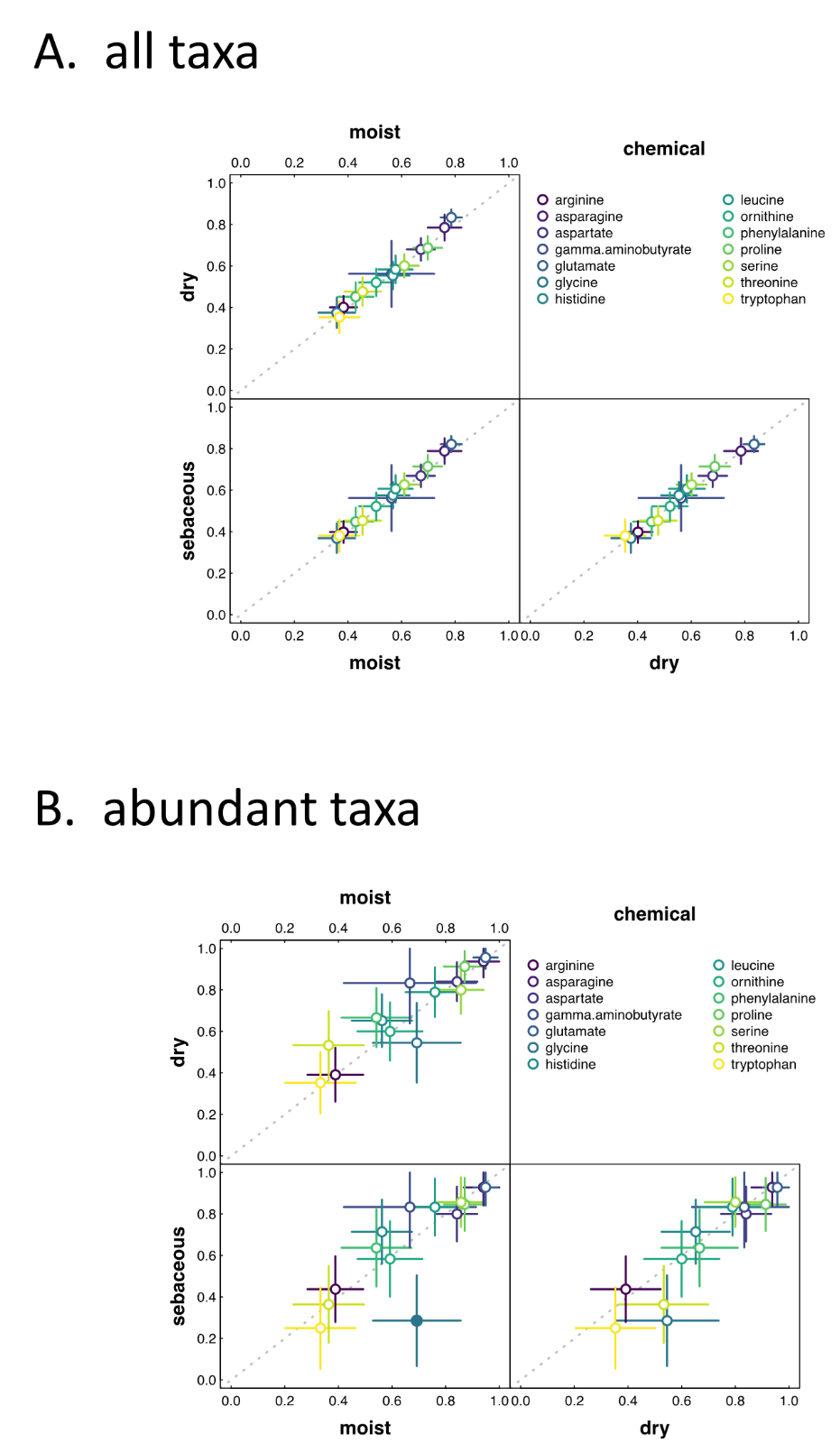


**Figure S5.7** Pairwise comparison (dry vs. moist, dry vs. sebaceous, moist vs. sebaceous) of amino acid use. Analyses are shown for (A) all skin bacteria (>0.001% of reads in at least one sample) and (B) abundant skin taxa (>0.1% of reads in at least one sample). Filled circles indicate a significant difference between sites; open circles indicate the opposite.

*
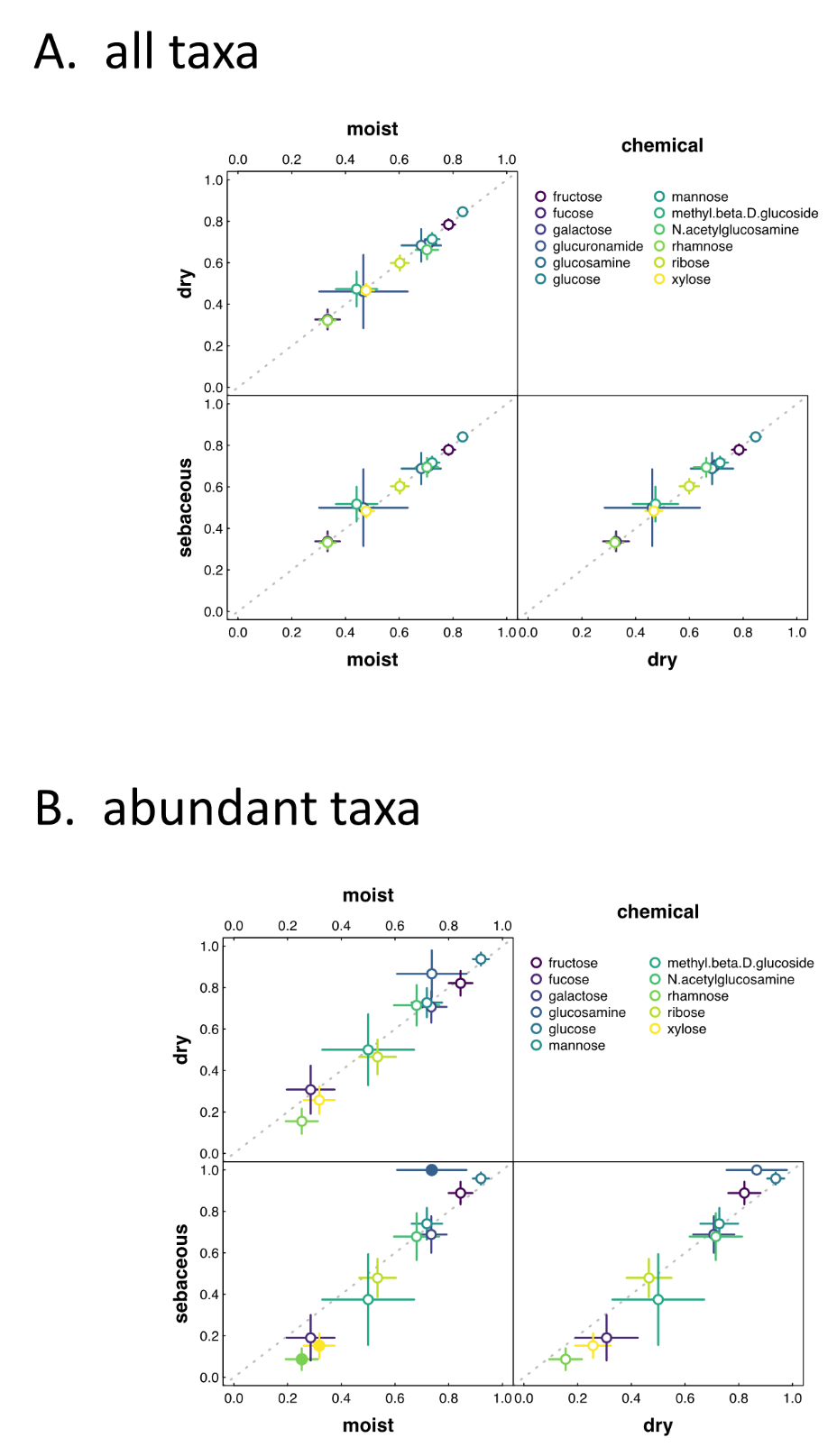
*

**Figure S5.8** Pairwise comparison (dry vs. moist, dry vs. sebaceous, moist vs. sebaceous) of monosaccharide use. Analyses are shown for (A) all skin bacteria (>0.001% of reads in at least one sample) and (B) abundant skin taxa (>0.1% of reads in at least one sample). Filled circles indicate a significant difference between sites; open circles indicate the opposite.

*
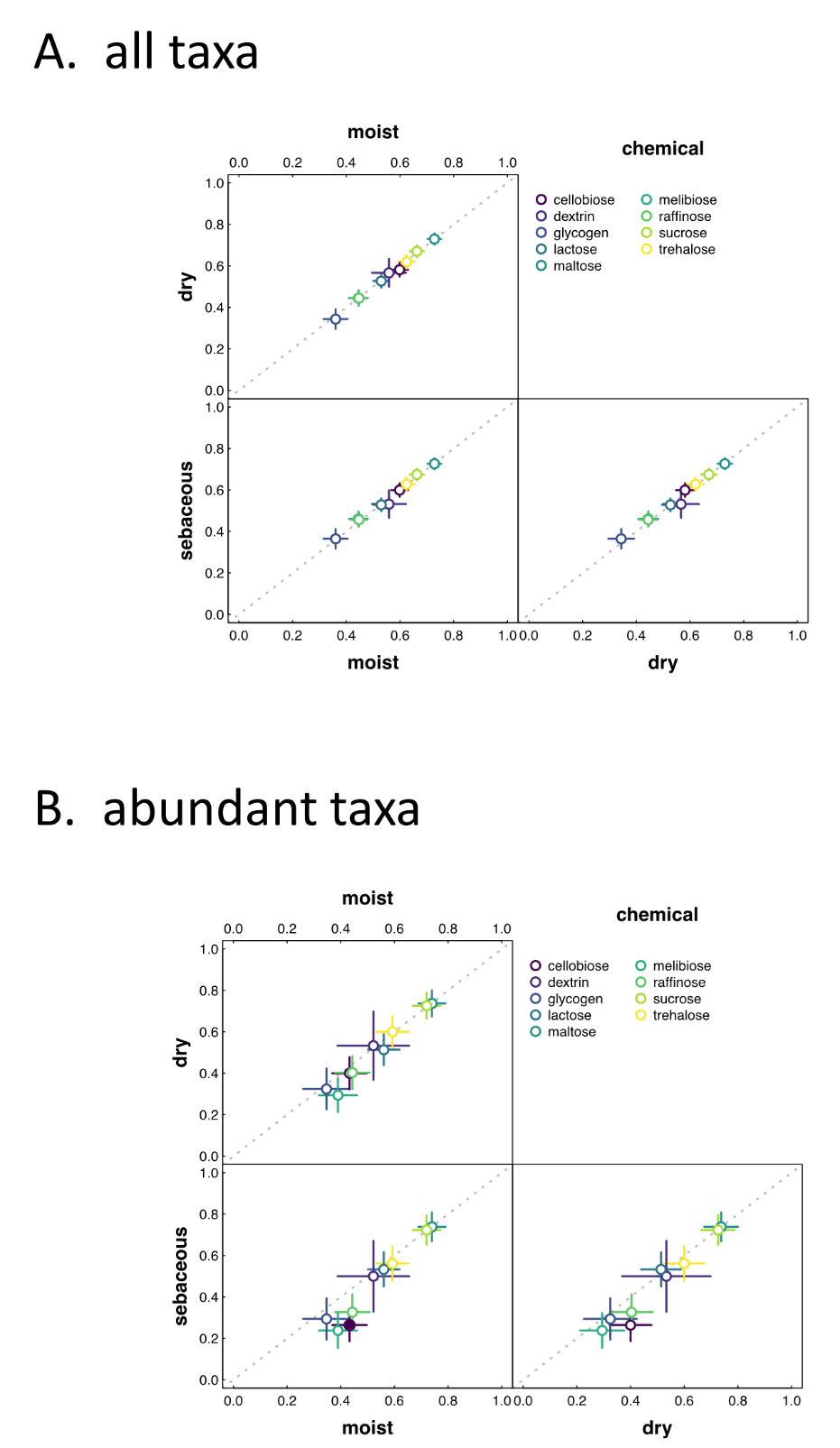
*

**Figure S5.9** Pairwise comparison (dry vs. moist, dry vs. sebaceous, moist vs. sebaceous) of oligo/polysaccharide use. Analyses are shown for (A) all skin bacteria (>0.001% of reads in at least one sample) and (B) abundant skin taxa (>0.1% of reads in at least one sample). Filled circles indicate a significant difference between sites; open circles indicate the opposite.


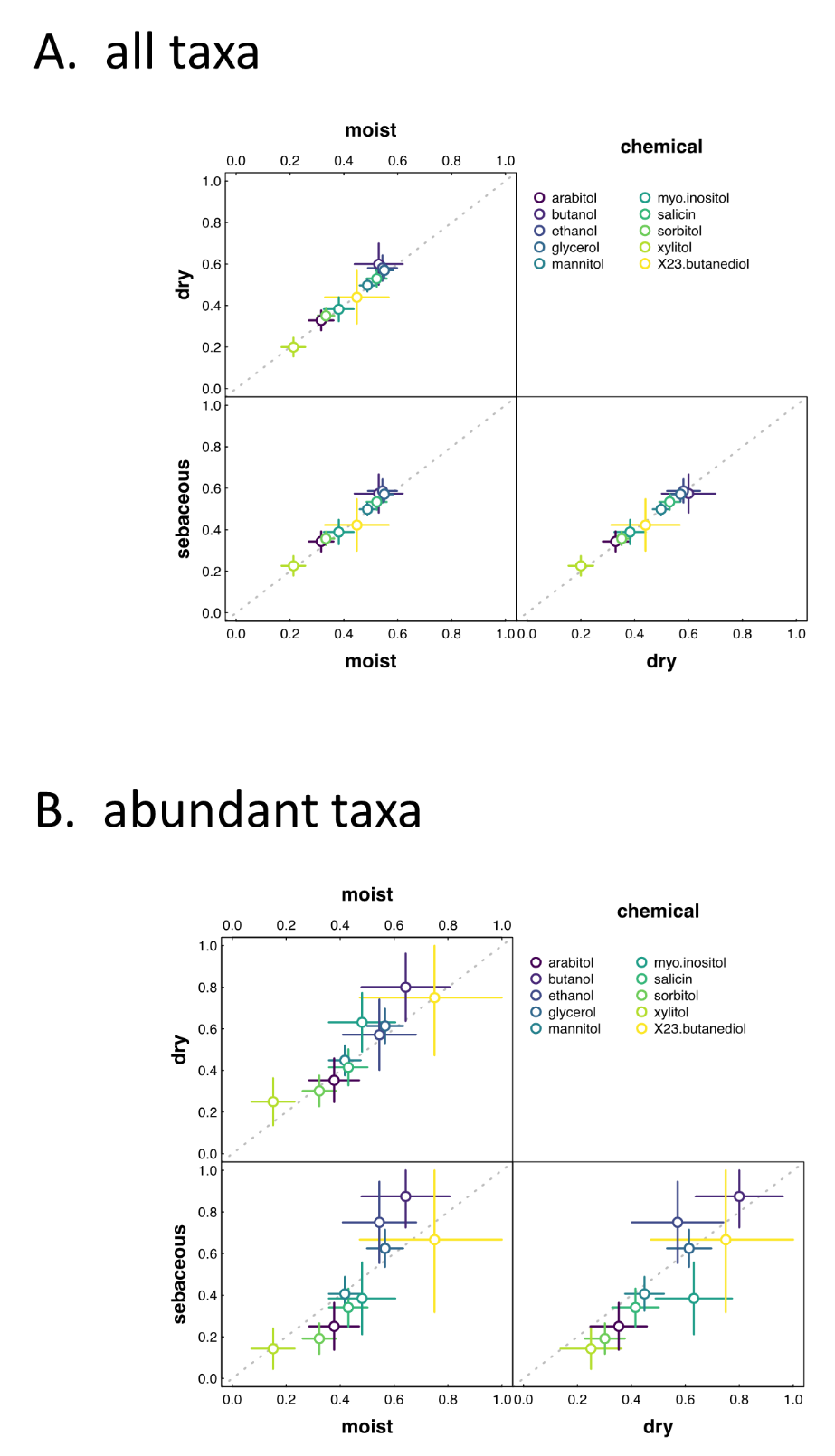


**Figure S5.10** Pairwise comparison (dry vs. moist, dry vs. sebaceous, moist vs. sebaceous) of alcohol use. Analyses are shown for (A) all skin bacteria (>0.001% of reads in at least one sample) and (B) abundant skin taxa (>0.1% of reads in at least one sample). Open circles indicate no significant difference between sites.


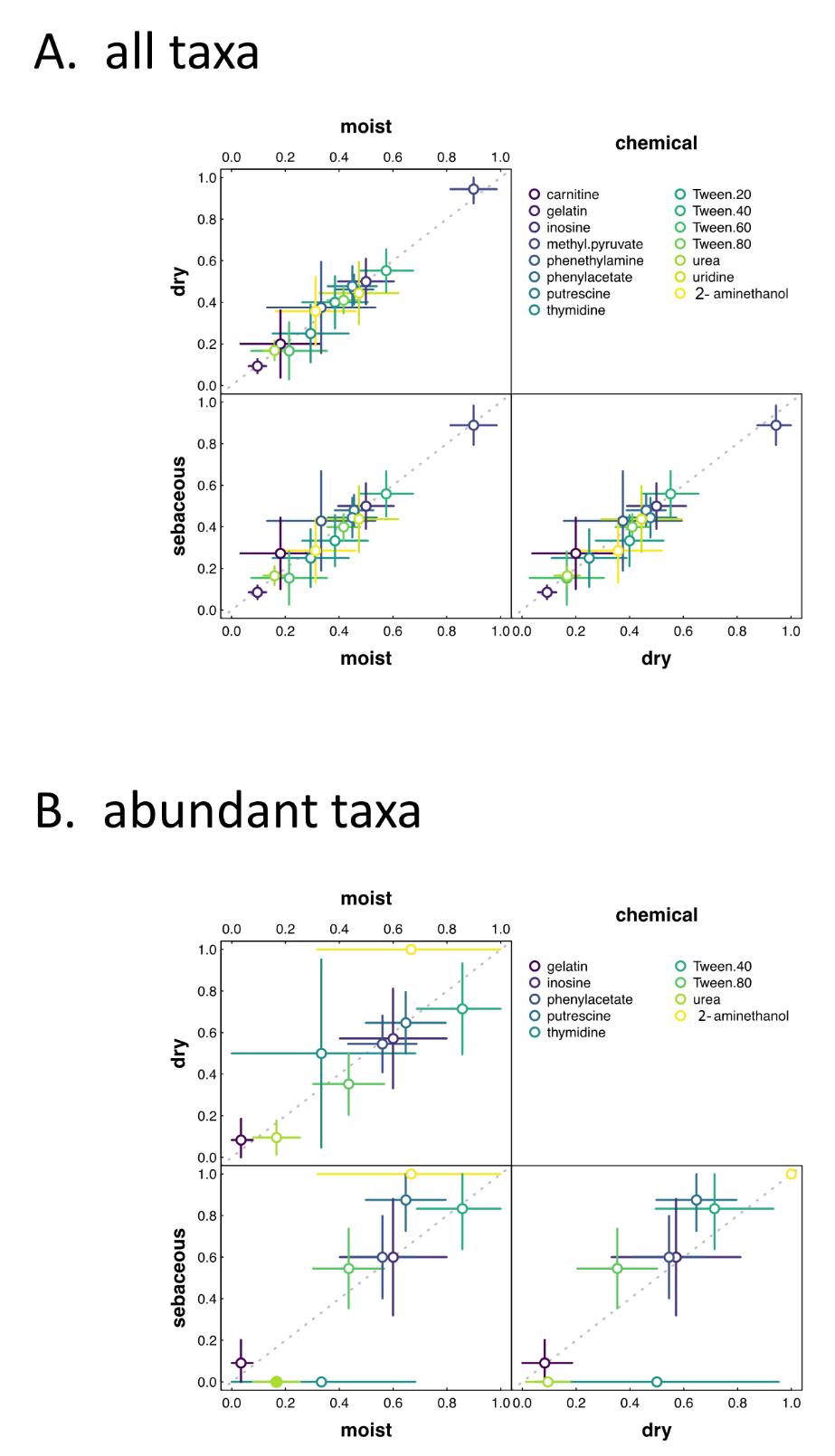


**Figure S5.11** Pairwise comparison (dry vs. moist, dry vs. sebaceous, moist vs. sebaceous) of use of specialized chemicals. Analyses are shown for (A) all skin bacteria (>0.001% of reads in at least one sample) and (B) abundant skin taxa (>0.1% of reads in at least one sample). Open circles indicate no significant difference between sites.

*Supplementary Information VI: Example Comparisons Between Our Database and the ProTrait Database*

In what follows, we compare our database to the ProTrait database using bacterial oxygen use and glucose use as examples. For the ProTrait database, we assume binarized predictions at a precision of ≥ 0.9.

**Oxygen Use**

Information about oxygen use was simultaneously missing from both databases for 86 taxa (8.8%). Oxygen use was missing from our database, but not from the ProTrait database for 51 taxa (5.2%). Oxygen use was missing from the ProTrait database but not from our database for 347 taxa (35.7%). Finally, oxygen use was simultaneously reported in both databases for 462 taxa (47.6%) . Of the 462 taxa that contained information on oxygen use in both databases, 27 taxa (5.8%) exhibited discrepancies. By far the largest type of discrepancy was facultative anaerobes being recorded as strict aerobes or anaerobes or strict aerobes or anaerobes being recorded as facultative anaerobes in one versus the other database. Indeed, there were only 5 blatant discrepancies (an aerobe being classified as an anaerobe or vice versa). Our database classified two organisms as anaerobic that were actually aerobes and one organism as aerobic that was actually an anaerobe. Meanwhile, the ProTrait database classified two organisms as anaerobic that were actually microaerophilic. Thus, while accuracy seems comparable between the two databases, at least for oxygen use, our database has much greater coverage.

**Glucose Use**

Information about the ability to use glucose as a substrate was simultaneously missing from both databases for 308 taxa (31.7%). Information on glucose use was missing from our database, but not from the ProTrait database for 20 taxa (2.0%). Information on glucose use was missing from the ProTrait database, but not from our database for 496 taxa (51.1%). Finally, information about glucose use was simultaneously reported in both databases for 122 taxa (12.5%). Of the 122 taxa that contained information on glucose use in both databases, 4 taxa (3.3%) exhibited discrepancies. One species was *Arthrobacter phenanthrenivorans*, which we reported as negative for glucose use. Although the only reference to glucose in our literature reported that acid was not produced from this substrate, we cannot rule out other findings in more recent papers. The other three species with discrepancies were all within the genus *Acinetobacter*. For all three of these, we reported positive glucose use, whereas ProTrait reported negative use. In our literature sources, we found evidence that all three strains can oxidize glucose, which we classify as positive use. Despite these minor differences, again we find good agreement between our database and the ProTrait database, suggesting that trait coverage, rather than accuracy, is the main benefit of manual curation.
